# Rab2A regulates the progression of nonalcoholic fatty liver disease downstream of AMPK-TBC1D1 axis by stabilizing PPARγ

**DOI:** 10.1371/journal.pbio.3001522

**Published:** 2022-01-21

**Authors:** Zi-Yue Chen, Ya-Ting Sun, Zi-Ming Wang, Jie Hong, Min Xu, Fu-Ting Zhang, Xian-Qing Zhou, Ping Rong, Qi Wang, Hong Yu Wang, Hua Wang, Shuai Chen, Liang Chen

**Affiliations:** 1 College of Life Science, Anhui Medical University, Hefei, China; 2 Department of Oncology, The First Affiliated Hospital of Anhui Medical University, Hefei, China; 3 College of Life Sciences, Wuhan University, Wuhan, China; 4 MOE Key Laboratory of Model Animal for Disease Study, Department of Endocrinology, Nanjing Drum Tower Hospital, Model Animal Research Center, School of Medicine, Nanjing University, Nanjing, China; 5 Inflammation and Immune Mediated Diseases Laboratory of Anhui Province, Hefei, China; Duke University, UNITED STATES

## Abstract

Nonalcoholic fatty liver disease (NAFLD) affects approximately a quarter of the population worldwide, and persistent overnutrition is one of the major causes. However, the underlying molecular basis has not been fully elucidated, and no specific drug has been approved for this disease. Here, we identify a regulatory mechanism that reveals a novel function of Rab2A in the progression of NAFLD based on energy status and PPARγ. The mechanistic analysis shows that nutrition repletion suppresses the phosphorylation of AMPK-TBC1D1 signaling, augments the level of GTP-bound Rab2A, and then increases the protein stability of PPARγ, which ultimately promotes the hepatic accumulation of lipids in vitro and in vivo. Furthermore, we found that blocking the AMPK-TBC1D1 pathway in TBC1D1^S231A^-knock-in (KI) mice led to a markedly increased GTP-bound Rab2A and subsequent fatty liver in aged mice. Our studies also showed that inhibition of Rab2A expression alleviated hepatic lipid deposition in western diet-induced obesity (DIO) mice by reducing the protein level of PPARγ and the expression of PPARγ target genes. Our findings not only reveal a new molecular mechanism regulating the progression of NAFLD during persistent overnutrition but also have potential implications for drug discovery to combat this disease.

## Abstract 2

Non-alcoholic fatty liver disease (NAFLD) affects approximately a quarter of the global population; persistent overnutrition is one of the major causes, but the molecular mechanism remains unclear. This study shows that overnutrition suppresses the phosphorylation of AMPK and TBC1D1, augmenting the level of GTP-bound Rab2A and increasing the stability of PPARγ, which ultimately promotes the hepatic accumulation of lipids.

## Introduction

Nonalcoholic fatty liver disease (NAFLD) is the main cause of chronic liver disease worldwide and has a global prevalence of 25.2% [1]. Clinically, patients with NAFLD often somehow suffer from other metabolic syndromes, such as obesity, type 2 diabetes, and insulin resistance [2]. Many studies have shown that genetic mutations can regulate the progression of NAFLD, including patatin-like phospholipase domain–containing 3 (PNPLA3), transmembrane 6 superfamily member 2 (TM6SF2), and membrane-bound O-acyltransferase domain–containing 7 (MBOAT7) [3]. However, prior to this study, these genes can explain only a few characteristics of NAFLD, and no effective therapies have been found for this disease. Therefore, there is an urgent need to understand the pathogenesis of NAFLD, especially that caused by persistent overnutrition, and to develop new therapeutic approaches to combat this disease.

Adenosine monophosphate (AMP)–activated protein kinase (AMPK) is a key metabolism-related regulator that senses energy/nutrient status and controls catabolism [4]. AMPK is activated via a canonical adenine nucleotide–sensing mechanism and noncanonical pathways by various stimuli, such as glucose starvation [5]. In general, AMPK is activated during nutrition depletion, and its activity is decreased under nutrition repletion. Furthermore, the functions of AMPK are primarily mediated via various downstream targets [6]. For example, AMPK is a critical regulator of glucose uptake in skeletal muscle at least in part through the phosphorylation of TBC1D1 (Tre-2/USP6, BUB2, cdc16 domain family member 1) [7]. TBC1D1 is a Rab-GTPase activating protein (RabGAP) that can be phosphorylated by AMPK at its serine-231 site in mice (a site homologous to serine 237 in human TBC1D1) [8]. Disruption of the AMPK-TBC1D1 signaling nexus in a mouse model enhances lipogenesis in adipose tissue and subsequently causes obesity and type 2 diabetes by promoting insulin-like growth factor 1 (IGF1) secretion [9].

PPARγ consists of PPARγ1 and PPARγ2, which are transcribed from the same gene under the control of different promoters and are master transcription factors in adipogenesis involved in lipogenesis, triglyceride (TG) synthesis, and lipid droplet formation [10]. Studies performed using various genetic mouse models demonstrate the importance of PPARγ in the regulation of fatty acid homeostasis and insulin sensitivity [11–13]. As a nuclear receptor, PPARγ can be regulated by natural ligands, such as unsaturated fatty acids [14]. Multiple regulatory mechanisms are involved in the control of PPARγ at both the transcriptional and posttranscriptional levels. The transcriptional regulation of PPARγ depends on multiprotein complexes, including coactivators such as PGC1α, CBP/p300, the SRC family, and TRAP220 and corepressors such as SMRT, RIP140, and NCoR [15]. In addition, the activity of PPARγ is modulated by various posttranslational modifications, such as phosphorylation at serine-112 and serine-273 [16,17] and protein cleavage [18–20]. Accumulating evidence shows that PPARγ is an important regulator of energy balance [21]. However, whether and how energy/nutrient-sensing AMPK regulates PPARγ remains unclear, and the molecular mechanisms of the degradation of PPARγ have not been fully elucidated to date.

Rab proteins, a major family of small GTPases, mainly regulate intracellular membrane trafficking and have been implicated in many human diseases, such as Parkinson disease and Carpenter syndrome [22]. However, until now, there has been no explicit evidence that relates Rab proteins to the progression of NAFLD. In this study, we identified a regulatory mechanism that reveals Rab2A in the progression of NAFLD through PPARγ protein in response to energy/nutrient status. Through genetic and cellular studies, we demonstrated that nutrition repletion inactivates AMPK-TBC1D1 signaling, augments the level of GTP-bound Rab2A, and then increases the protein stability of PPARγ and the expression of PPARγ target genes, and ultimately contributes to the development of NAFLD.

## Results

### Blocking the AMPK-TBC1D1 axis leads to hepatic lipid deposition in aged mice

**Fig 1:**
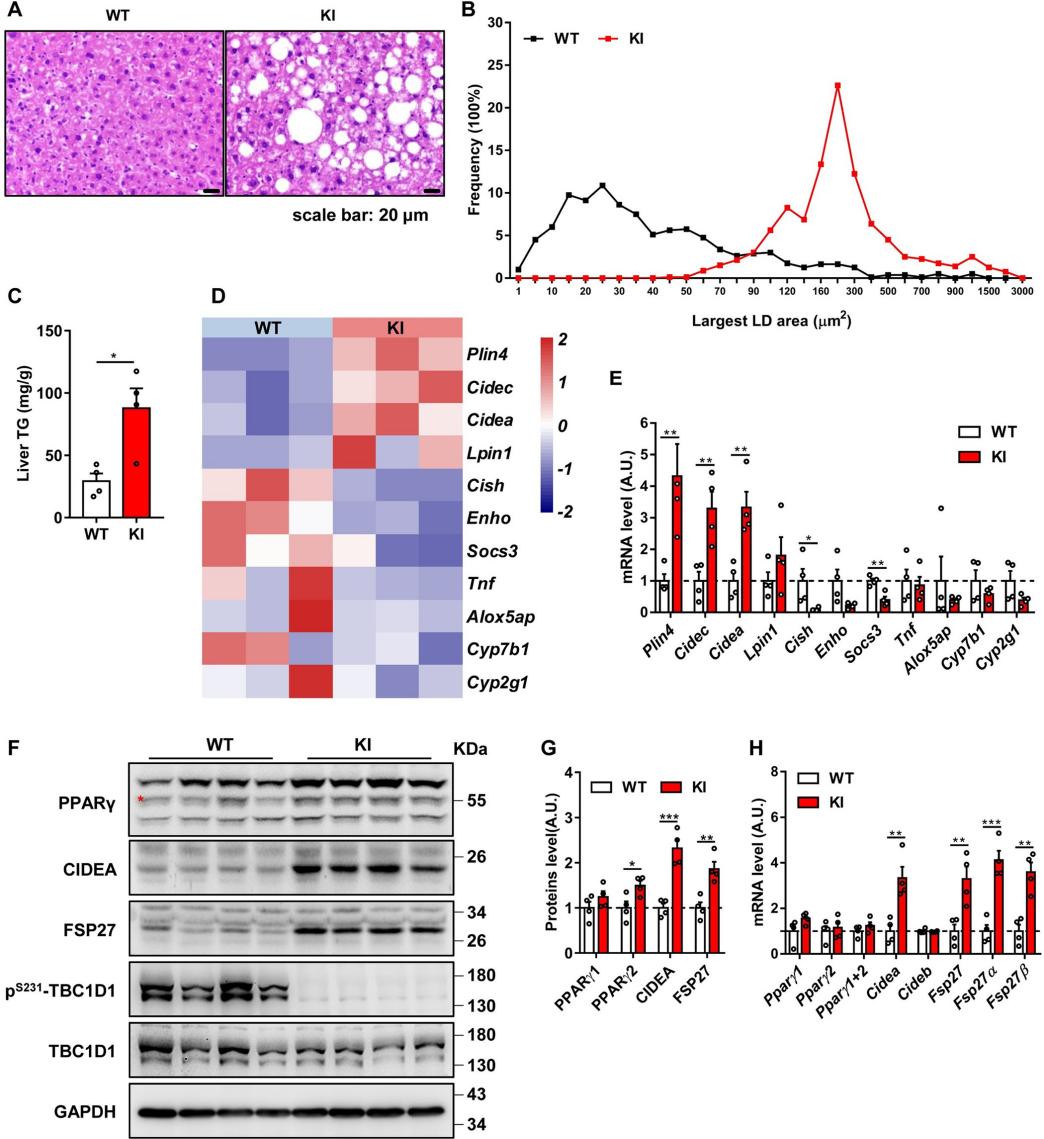
Blocking the AMPK-TBC1D1 axis in aged mice leads to hepatic lipid accumulation. **(A)** Hematoxylin–eosin staining of liver sections from WT and TBC1D1 KI male mice aged 18 months (random feed, *n* = 4 per group). Representative images are shown. **(B)** Size profiles of lipid droplets in liver sections (A). At least 200 droplets from each mouse were assessed. The percentages of lipid droplets of various sizes are shown. **(C)** TG levels in the livers of WT and TBC1D1 KI male mice aged 18 months (random feed, *n* = 4 per group). **(D)** Heatmap shows the genes with significant differences in expression between the livers of WT and TBC1D1-KI male mice at 18 months of age based on RNA sequencing data (random feed, *n* = 3 per group). **(E)** mRNA expression levels of genes in the heatmap were confirmed by Q-PCR (random feed, *n* = 4 per group). **(F)** Increased protein level of PPARγ in the liver of TBC1D1-KI mice aged 18 months. The data were obtained by immunoblotting (random feed, *n* = 4 per group). The red asterisk indicates nonspecific signal. **(G)** Statistical analysis of protein levels shown in F. **(H)** mRNA expression levels of PPARγ-related genes were confirmed by Q-PCR (random feed, *n* = 4 per group). The data were analyzed with unpaired 2-tailed Student *t* test and are presented as the mean ± s.e.m.s. “*” indicates *p* < 0.05, “**” indicates *p* < 0.01, and “***” indicates *p* < 0.001. Raw data are given in S1 Excel spreadsheet with raw data from all figures. AMPK, adenosine monophosphate–activated protein kinase; KI, knock-in; TG, triglyceride; WT, wild-type.

Our previous study demonstrated that disruption of the AMPK-TBC1D1 axis increases lipogenesis in the adipose tissue and causes obesity and type 2 diabetes by promoting IGF1 vesicle secretion in a TBC1D1^S231A^-knock-in (KI) mouse model from 2 to 10 months [9]. Here, to further explore the underlying role of AMPK-TBC1D1 signaling in hepatic steatosis, we detected NAFLD-related phenotypes in TBC1D1-KI mice at 4 to 6 months, 12 months, and 18 months of age. The observations of 4- to 6-month-old KI mice revealed normal lipid droplets in liver sections (S1A Fig), consistent with normal levels of TGs (S1B Fig). However, 12- and 18-month-old KI mice showed markedly increased accumulation of larger lipid droplets in liver sections (Fig 1A and 1B, S1C Fig) accompanied by severely increased TG levels (Fig 1C, S1D Fig). These abovedescribed data indicate that blocking the AMPK-TBC1D1 axis leads to hepatic lipid accumulation in aged mice.

To investigate the underlying molecular mechanisms that mediate the storage of lipids in the liver of aged TBC1D1-KI mice, we first detected the mRNA levels of general fatty acid metabolism-related genes, such as genes involved in lipolysis and fatty acid oxidation (S1E Fig), genes involved in fatty acid uptake, secretion and storage (S1F Fig), genes encoding transcription factors (S1G Fig), and genes involved in fatty acid synthesis (S1H Fig). The results showed no significant differences between the 2 genotypes. Our previous studies revealed increased activities of IGF1-AKT/PKB-mTOR-SREBP-1c signaling and lipogenesis in the adipose tissue of TBC1D1-KI mice at 4 to 6 months [9], and we thus also detected the protein levels of core lipogenic proteins such as fatty acid synthase (FASN) and cytoplasmic acetyl-CoA synthase (ACS1). All of these proteins showed normal expression levels in the livers of 18-month-old (S1I and S1J Fig) and 12-month-old mice (S1K and S1L Fig).

We then performed RNA sequencing of the livers of 18-month-old mice, and the KEGG analysis showed the marked enrichment of genes related to the PPARγ signaling pathway (S2A Fig). Furthermore, a heatmap analysis revealed substantial increases in lipid storage genes, such as *Cidea*, *Cidec*, and *Plin4*, which are targets of PPARγ (Fig 1D), and the Q-PCR results confirmed these findings in the livers of 18-month-old (Fig 1E) and 12-month-old mice (S2B Fig). Then, we found significantly increased PPARγ protein levels with no obvious differences in the PPARγ mRNA levels in the 12-month-old (S2C–S2E Fig) and 18-month-old TBC1D1-KI mice (Fig 1F–1H), and these effects were followed by increased PPARγ activity. Together, the abovementioned data suggest that blocking the AMPK-TBC1D1 axis leads to NAFLD in aged mice, possibly through the activation of PPARγ signaling.

### The AMPK-TBC1D1 axis regulates the protein stability and functions of PPARγ

To clearly elucidate the relationship between the AMPK-TBC1D1 axis and PPARγ, we constructed a relevant system in human hepatoma cell line (HepG2), human embryonic kidney cell line (HEK293T), and primary hepatocytes. Primarily, we found that transient overexpression of TBC1D1 in HepG2 cells markedly increased the endogenous protein level of PPARγ (S3A Fig). Because PPARγ consists of 2 isoforms, PPARγ1 and PPARγ2, we then explored the regulation of PPARγ1 and PPARγ2 individually. The results showed that overexpression of TBC1D1 augmented the exogenous protein level of PPARγ1 in HepG2 cells (S3B Fig) and HEK293T cells (S3C and S3D Fig), and similar regulation of PPARγ2 was also detected in both HepG2 cells (S3E Fig) and HEK293T cells (S3F and S3G Fig). The above data suggest that TBC1D1 regulates the protein level of PPARγ.

**Fig 2:**
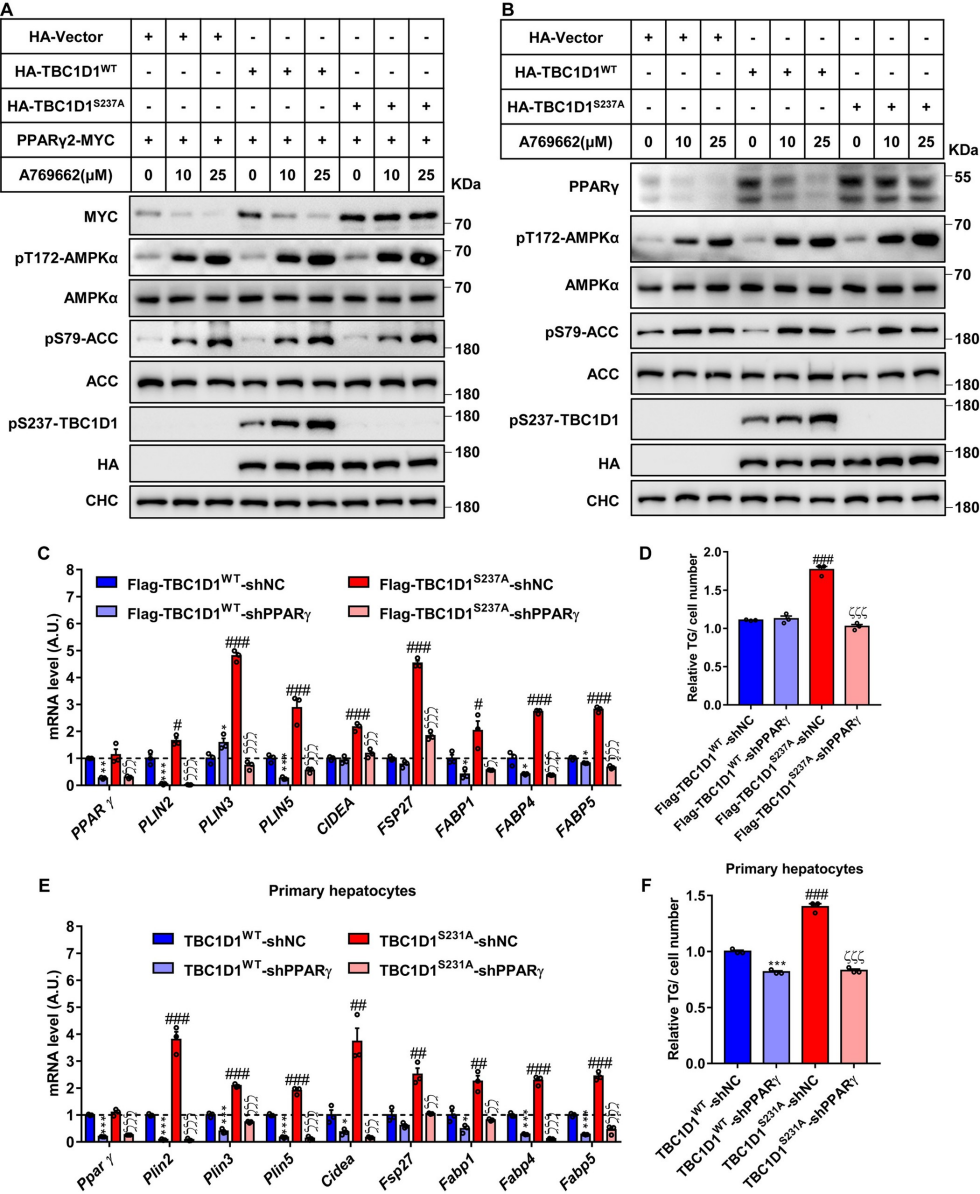
The AMPK-TBC1D1 axis regulates the protein stability and functions of PPARγ. **(A, B)** Phosphorylation of TBC1D1 at serine 237 site attenuates the protein levels of exogenous PPARγ2 (A) and endogenous PPARγ (B). HEK293T (A) or HepG2 (B) cells were cultured, transfected with the indicated plasmids, and then stimulated with different concentrations of A769662 for 16 hours. The cells were harvested and analyzed by immunoblotting. **(C, D)** Knockdown of PPARγ rescues the effects of lipid storage in HepG2 cells overexpression the TBC1D1-S237A protein. HepG2 cells were cultured and transfected with the indicated lentivirus-expressing plasmids, and then the positive cells were chosen and harvested for Q-PCR (C) and TG testing (D). **(E, F)** Knockdown of PPARγ rescues the effects of lipid storage in TBC1D1-S231A primary hepatocytes. Primary hepatocytes were isolated, cultured, and transfected with the indicated lentivirus-expressing plasmids. Then the cells were chosen and harvested for Q-PCR (E) and TG testing (F). The TG level of control cells was normalized to 1, and all the above statistical data were analyzed with unpaired 2-tailed Student *t* test (*n* = 3 per group) and are presented as the means ± s.e.m.s. shNC versus shPPARγ in Flag-TBC1D1^WT^-overexpressing HepG2 cells or WT primary hepatocytes (“*” indicates *p* < 0.05, “**” indicates *p* < 0.01, and “***” indicates *p* < 0.001). shNC versus shPPARγ in Flag-TBC1D1^S237A^-overexpressing HepG2 cells or TBC1D1-S231A primary hepatocytes (“ζ” indicates *p* < 0.05, “ζζ” indicates *p* < 0.01, and “ζζζ” indicates *p* < 0.001). Flag-TBC1D1^WT^ versus Flag-TBC1D1^S237A^ in HepG2 cells or WT versus TBC1D1-S231A primary hepatocytes (“#” indicates *p* < 0.05, “##” indicates *p* < 0.01, and “###” indicates *p* < 0.001). Raw data are given in S1 Excel spreadsheet with raw data from all figures. All experiments were performed at least 3 times with similar results. AMPK, adenosine monophosphate–activated protein kinase; WT, wild-type.

To uncover the underlying mechanism in detail, we selected PPARγ2, which is the longer form, as the protein of interest in the following studies. TBC1D1^S237A^, which refers to TBC1D1 with an alanine substitution at the S-237 site, is not phosphorylatable by AMPK at this site. Overexpression of TBC1D1^S237A^ markedly up-regulated the protein levels of exogenous PPARγ2 (S3H Fig) and endogenous PPARγ (S3I Fig) as compared to wild-type (WT) TBC1D1. To investigate a possible causal relationship between the AMPK-TBC1D1 axis and PPARγ protein, we utilized an AMPK activator A769662 to treat cells expressing an empty vector, or WT TBC1D1, or TBC1D1^S237A^ mutant. A769662 treatment expectedly increased the phosphorylation of AMPK and its bona fide substrate acetyl-CoA carboxylase (ACC) in a dose-dependent manner, and the activation states of AMPK were comparable among the cells expressing the empty vector, or WT TBC1D1, or TBC1D1^S237A^ mutant (Fig 2A and 2B). Notably, activation of AMPK by A769662 caused a gradual decrease of exogenous PPARγ2 (Fig 2A) and endogenous PPARγ (Fig 2B), which correlates with the dosage of A769662. Expression of WT TBC1D1 elevated both exogenous PPARγ2 and endogenous PPARγ, which were still decreased upon AMPK activation by A769662 treatment (Fig 2A and 2B). Importantly, the A769662-induced diminution of exogenous PPARγ2 and endogenous PPARγ was prevented when the AMPK-insensitive TBC1D1^S237A^ mutant was expressed in cells (Fig 2A and 2B).

We next examined whether TBC1D1 KI mutation–induced expression of lipid storage genes was indeed mediated by PPARγ. To this end, we employed 2 types of cell models, namely HepG2 cells expressing TBC1D1^WT^ or TBC1D1^S237A^ proteins and primary hepatocytes from WT and TBC1D1^S231A^-KI mice. Expression of the TBC1D1^S237A^ mutant protein significantly increased PPARγ-targeted genes, including *PLIN3*, *PLIN5*, *CIDEA*, *FSP27/CIDEC*, *FABP1*, *FABP4*, and *FABP5* in HepG2 cells (Fig 2C, S3J Fig) and elevated cellular TG contents (Fig 2D), which were prevented by down-regulation of PPARγ via short hairpin RNA (shRNA). Similarly, knockdown of PPARγ rescued the expression of lipid storage genes such as *Plin3*, *Plin5*, *Cidea*, *Fsp27*/*Cidec*, *Fabp1*, *Fabp4*, and *Fabp5* (Fig 2E) and prevented TG accumulation in primary hepatocytes from TBC1D1^S231A^-KI mice (Fig 2F).

Taken together, these data firmly establish a causal role of the AMPK-TBC1D1 axis in the regulation of PPARγ protein, and disruption of the AMPK-TBC1D1 nexus increases PPARγ protein to promote TG accumulation by elevating the expression of lipid storage genes in hepatocytes.

### Rab2A, as a downstream protein of the AMPK-TBC1D1 axis, regulates the protein level of PPARγ

**Fig 3:**
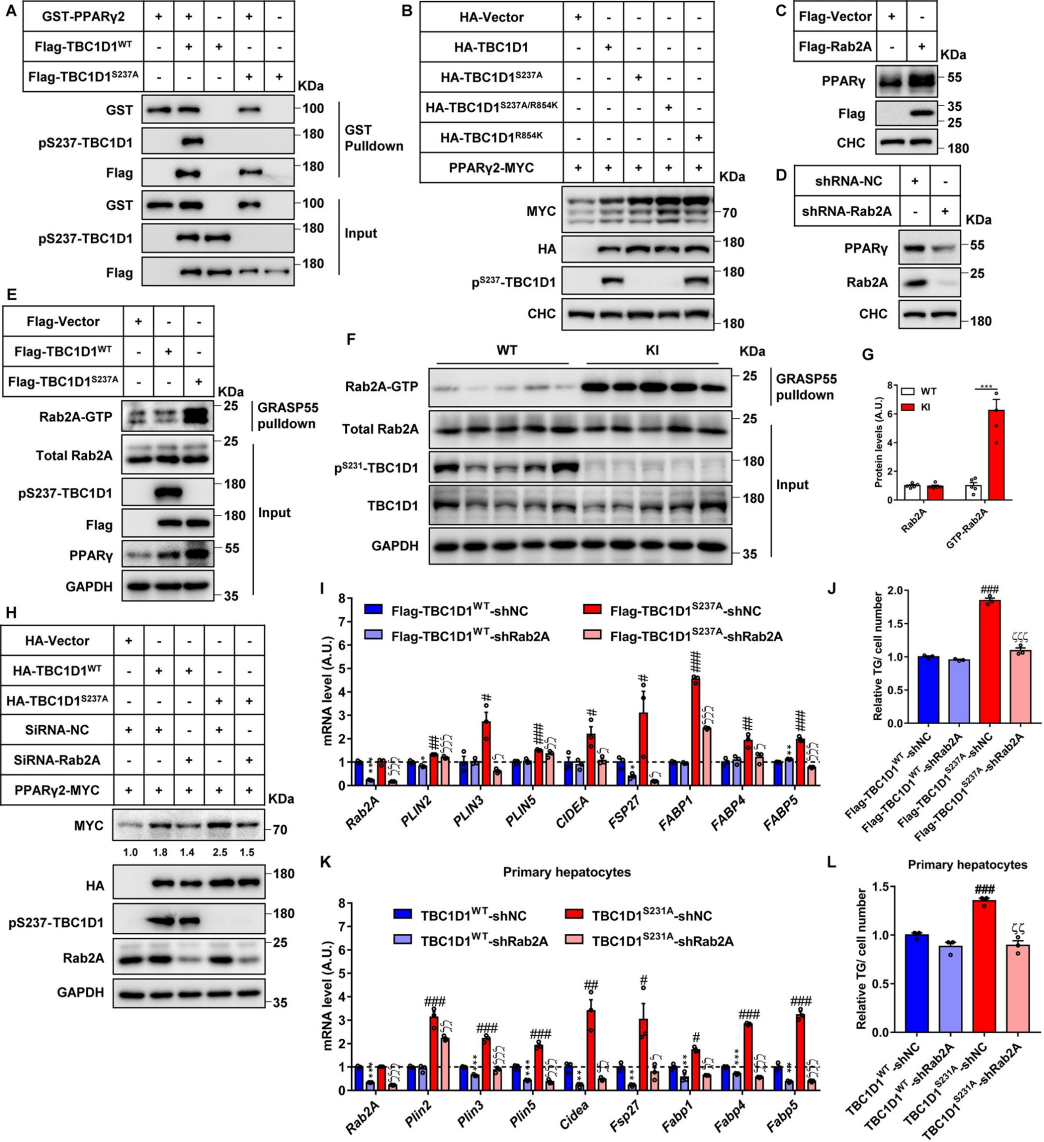
Rab2A regulates the protein level and functions of PPARγ as the downstream protein of the AMPK-TBC1D1 axis. **(A)** Protein binding between TBC1D1 and PPARγ2 is independent of the phosphorylation at the serine 237 site of TBC1D1. GST-PPARγ2 was purified via a prokaryotic expression system, and Flag-TBC1D1 was pulled down with anti-Flag beads in HepG2 cells. Then an in vitro binding assay was performed in tubes as described in the methods, and the results were analyzed by immunoblotting. **(B)** GTPase activity of TBC1D1 mediates the regulation of PPARγ2. HEK293T cells were cultured, transfected with the indicated plasmids for 2 days, harvested and analyzed by immunoblotting. **(C)** Overexpression of Rab2A increases the protein stability of PPARγ. HepG2 cells were cultured and transfected with lentivirus-expressing plasmids, and the positive cells were then selected, collected, and analyzed by immunoblotting. **(D)** Knockdown of Rab2A decreases the protein stability of PPARγ. HepG2 cells were cultured and transfected with lentivirus-expressing shRNA, and the positive cells were then selected, collected, and analyzed by immunoblotting. **(E)** Blocking phosphorylation at the serine 237 site increases the level of GTP-bound form of Rab2A. HepG2 cell lines stably expressing TBC1D1-WT or TBC1D1-S237A proteins were harvested, and the data were obtained by GST-GRASP55 pulldown and immunoblotting assays. **(F, G)** Significantly increased levels of GTP-bound form of Rab2A in the livers of TBC1D1-KI male mice aged 18 months. The data are shown by GST-GRASP55 pulldown and immunoblotting assays (F), and the percentage of GTP-Rab2A was quantified and is shown (G) (*n* = 5 per group, “***” indicates *p* < 0.001). **(H)** Knockdown of Rab2A attenuates the function of TBC1D1 in the regulation of exogenous PPARγ2 stability. HEK293T cells were cultured and transfected with the indicated plasmids and siRNAs for 2 days, and the cells were then harvested and analyzed by immunoblotting. The level of MYC was quantified and normalized to lane 1. **(I, J)** Knockdown of Rab2A rescues the effects of lipid storage in HepG2 cells with TBC1D1-S237A protein overexpression. HepG2 cells were cultured and transfected with the indicated lentivirus-expressing plasmids, and then the positive cells were chosen and harvested for Q-PCR (I) and TG testing (J). **(K, L)** Knockdown of Rab2A rescues the effects of lipid storage in TBC1D1-S231A primary hepatocytes. Primary hepatocytes were isolated, cultured, and transfected with the indicated lentivirus-expressing plasmids. Then, the cells were chosen and harvested for Q-PCR (K) and TG testing (L). The TG level of control cells was normalized to 1, and all the above statistical data were analyzed with unpaired 2-tailed Student *t* test (*n* = 3 per group) and are presented as the means ± s.e.m.s. shNC versus shRab2A in Flag-TBC1D1^WT^-overexpressing HepG2 cells or WT primary hepatocytes (“*” indicates *p* < 0.05, “**” indicates *p* < 0.01, and “***” indicates *p* < 0.001). shNC versus shRab2A in Flag-TBC1D1^S237A^-overexpressing HepG2 cells or TBC1D1-S231A primary hepatocytes (“ζ” indicates *p* < 0.05, “ζζ” indicates *p* < 0.01, and “ζζζ” indicates *p* < 0.001). Flag-TBC1D1^WT^ versus Flag-TBC1D1^S237A^ in HepG2 cells or WT versus TBC1D1-S231A primary hepatocytes (“#” indicates *p* < 0.05, “##” indicates *p* < 0.01, and “###” indicates *p* < 0.001). Raw data are given in S1 Excel spreadsheet with raw data from all figures. All the above cellular experiments were performed at least twice with similar results. AMPK, adenosine monophosphate–activated protein kinase; KI, knock-in; siRNA, small interfering RNA; TG, triglyceride; WT, wild-type.

We next sought to find out how TBC1D1 regulates PPARγ protein levels. Interestingly, we found that Flag-TBC1D1 could interact with exogenous PPARγ2-MYC protein (S3K Fig) as well as endogenous PPARγ protein (S3L Fig). Moreover, the in vitro binding assay also demonstrated a direct interaction between TBC1D1 and PPARγ2 (Fig 3A). Notably, overexpression of TBC1D1 attenuated the degradation of PPARγ2 when cells were treated with cycloheximide (CHX) (S3M and S3N Fig), suggesting that TBC1D1 may regulate the protein stability of PPARγ.

The in vitro binding assay showed that TBC1D1^S237A^ mutant protein still possessed the ability to interact with PPARγ in a manner similar to WT TBC1D1 (Fig 3A), suggesting that TBC1D1-S237 phosphorylation does not affect the TBC1D1 interaction with PPARγ. TBC1D1 is a GTPase-activating protein (GAP) and exhibits significant GAP activity toward Rab2A, Rab8A, Rab8B, Rab10, and Rab14 in an in vitro assay [23]. Overexpression of a GAP-inactive TBC1D1^R854K^ mutant increased PPARγ2 protein to an extent similar to TBC1D1^S237A^ mutant protein (Fig 3B). It is currently not clear whether the TBC1D1^R854K^ mutant exerts its effect on PPARγ2 through a GAP-independent mechanism or via a dominant-negative mechanism. To further study the possible mechanisms by which TBC1D1 regulates the PPARγ protein, we then screened for Rabs downstream of TBC1D1, which might regulate the PPARγ protein. A total of 23 Rabs were examined through their overexpression, including Rab2A (S4A Fig), Rab2B (S4B Fig), Rab8A (S4C Fig), Rab8B (S4D Fig), Rab10 (S4E Fig), Rab14 (S4F Fig), Rab1A (S4G Fig), Rab24 (S4H Fig), Rab35 (S4I Fig), Rab7A (S4J Fig), Rab15 (S4K Fig), Rab40A (S4L Fig), Rab1B (S5A Fig), Rab5A (S5B Fig), Rab9A (S5C Fig), Rab9B (S5D Fig), Rab11B (S5E Fig), Rab22B (S5F Fig), Rab32 (S5G Fig), Rab4B (S5H Fig), Rab39A (S5I Fig), Rab13 (S5J Fig), and Rab23 (S5K Fig). The screening results showed that only Rab2A overexpression up-regulated the protein level of PPARγ2-MYC (S4A Fig). In agreement, endogenous PPARγ was increased in Rab2A-overexpressing cells (Fig 3C) but decreased in Rab2A-knockdown cells (Fig 3D). Notably, overexpression of Rab2A augmented both the cytoplasmic and nuclear protein levels of PPARγ2-MYC (S5L Fig).

We then investigated how the AMPK-TBC1D1 axis regulates Rab2A via a pulldown assay utilizing the bait protein GST-GRASP55, which binds to the GTP-bound form of Rab2A [24]. As expected, GST-GRASP55 preferentially interacted with Rab2A-Q65L, the GTP-bound form, over Rab2A-S20N, the GDP-bound form (S6A Fig). In HepG2 cells, overexpression of the TBC1D1^S237A^ protein but not WT TBC1D1 significantly increased the GTP-bound form of Rab2A (Fig 3E). Moreover, the GTP-bound form of Rab2A was markedly increased, while total Rab2A levels were unaltered, in the livers of TBC1D1-KI mice (18 months old) (Fig 3F and 3G). These data show that the TBC1D1-S231A mutation causes activation of Rab2A in cells, although Rab2A might not be a physiological substrate of TBC1D1.

Coexpression of Rab2A with TBC1D1 exhibited a synergistic effect on PPARγ2-MYC protein (S6B Fig). The increases of exogenous PPARγ2 (Fig 3H, S6D and S6E Fig) and endogenous PPARγ (S6C Fig) induced by TBC1D1 WT or S237A proteins were attenuated by knockdown of Rab2A via shRNA. Importantly, knockdown of Rab2A in HepG2 cells prevented the induction of lipid storage genes elicited by the TBC1D1^S237A^ mutant protein, including *PLIN3*, *CIDEA*, *FSP27/CIDEC*, *FABP1*, and *FABP5* (Fig 3I) and blocked TG accumulation (Fig 3J). Similarly, knockdown of Rab2A also restored the expression of lipid storage genes such as *Plin2*, *Plin3*, *Plin5*, *Cidea*, *Fsp27*/*Cidec*, *Fabp1*, *Fabp4*, and *Fabp5* (Fig 3K) and prevented TG accumulation in primary hepatocytes from TBC1D1^S231A^-KI mice (Fig 3L). These data demonstrate that Rab2A functions genetically downstream of TBC1D1 to regulate PPARγ for hepatic lipid storage.

### GTP-bound form of Rab2A binds and inhibits the proteasomal degradation of PPARγ

**Fig 4:**
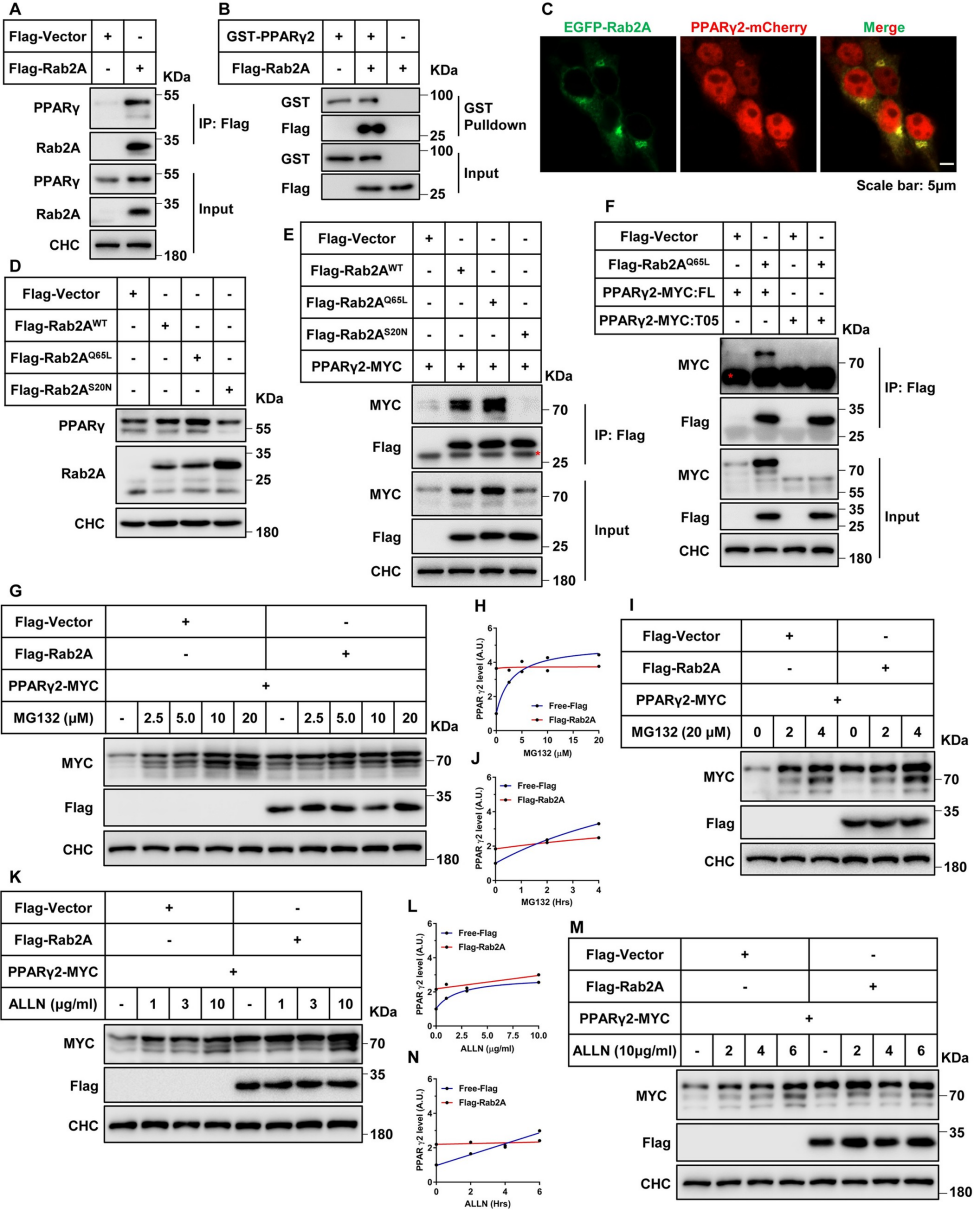
GTP-bound Rab2A binds with PPARγ and increases its stability. **(A)** Binding between Rab2A and endogenous PPARγ. HepG2 cells stably expressing Flag-Rab2A were harvested, immunoprecipitated with anti-Flag beads, and analyzed by immunoblotting. **(B)** In vitro binding assay between Rab2A and PPARγ2. GST-PPARγ2 was purified via a prokaryotic expression system, and Flag-Rab2A was pulled down with anti-Flag beads in HepG2 cells. Then, an in vitro binding assay was performed in tubes as described in the methods, and the results were analyzed by immunoblotting. The experiment was performed twice with similar results. **(C)** Colocalization of Rab2A and PPARγ2 in HEK293T cells. Representative images are shown. **(D)** GTP-bound form of Rab2A increases the protein stability of endogenous PPARγ. HepG2 cell lines with stable Rab2A expression were collected and analyzed by immunoblotting. **(E)** GTP-bound form of Rab2A binds with PPARγ2. HEK293T cells were cultured, transfected with the indicated plasmids for 2 days, lysed, immunoprecipitated with anti-Flag beads, and analyzed by immunoblotting. The red asterisk indicates a nonspecific signal. **(F)** Binding assay between Rab2A^Q65L^ and PPARγ2-Truncation 5. HEK293T cells were cultured and transfected with the indicated plasmids for 2 days, and the cells were then lysed, immunoprecipitated with anti-Flag beads, and analyzed by immunoblotting. The red asterisk indicates a nonspecific signal. **(G–N)** Rab2A regulates the proteasomal degradation of PPARγ2. HEK293T cells were cultured and transfected with the indicated plasmids for 2 days, and the cells were then stimulated with MG132 (G) or ALLN (K) for various concentrations and with various times of MG132 (I) or ALLN (M). The cells were harvested and analyzed by immunoblotting. (H) Quantification of PPARγ2 level in G. (J) Quantification of PPARγ2 level in I. (L) Quantification of PPARγ2 level in K. (N) Quantification of PPARγ2 levels in M. The ratio in lane 1 was defined as 1. Raw data are given in S1 Excel spreadsheet with raw data from all figures. All the above cellular experiments were performed at least twice with similar results. WT, wild-type.

We next sought to delineate the mechanism how Rab2A regulates PPARγ protein. In a co-immunoprecipitation assay, endogenous PPARγ was found in the immunoprecipitates of Flag-Rab2A (Fig 4A). In the in vitro GST-pulldown assay, GST-PPARγ2 could also bind to Flag-Rab2A (Fig 4B). Furthermore, imaging studies revealed that a portion of PPARγ2-mCherry existed in the cytosol and colocalized with EGFP-Rab2A that is mainly found in the cytosol (Fig 4C).

As a small G protein, Rab2A can switch between the GTP-bound active form and the GDP-bound inactive form [25]. The GTP-bound form, but not GDP-bound form, of Rab2A markedly augmented protein levels of exogenous PPARγ2-MYC (S7A Fig) and endogenous PPARγ (Fig 4D). Moreover, the GTP-bound form, but not GDP-bound form, of Rab2A interacted with PPARγ2-MYC in the co-immunoprecipitation assay (Fig 4E). PPARγ contains several domains, namely N terminal region (NTR), DNA-binding domain (DBD), ligand-binding domain (LBD), and a special region at the carboxyl terminus known as activation function 2 (AF-2) [15]. Besides these domains commonly found in both PPARγ isoforms, PPARγ2 has an additional 30 amino acids at its N-terminus as compared to PPARγ1. Interestingly, analyses with internal deletion mutants revealed that PPARγ2 with deletion of the AF-2 domain, but not other domains, lost its ability to respond to Rab2A (Fig 4F, S7B and S7C Fig). Notably, deficiency of the AF-2 domain prevented the binding of PPARγ2 to Rab2A in the co-immunoprecipitation assay (Fig 4F). These data suggest that the GTP-bound active form of Rab2A might interact with the AF-2 domain of PPARγ to increase the protein level of PPARγ.

We next examined whether Rab2A might regulate PPARγ stability to control its protein level. To this end, we first determined the effect of Rab2A overexpression on the degradation rate of PPARγ2-MYC. Addition of CHX triggered a rapid decrease of PPARγ2-MYC protein, and overexpression of Rab2A markedly slowed down the CHX-induced decrease of PPARγ2-MYC (S7D and S7E Fig). These data demonstrate that Rab2A indeed regulates PPARγ stability. We next examined whether Rab2A regulates PPARγ stability through the lysosome or proteasome pathway. Treatments with the inhibitors of proteasome, MG132 and ALLN (Ac-LLnL-CHO), both caused accumulation of PPARγ2-MYC in a concentration- and time-dependent manner (Fig 4G–4N). Notably, overexpression of Rab2A markedly attenuated the effects of these proteasome inhibitors on the accumulation rate of PPARγ2-MYC (Fig 4G–4N). By contrast, overexpression of Rab2A did not alleviate the accumulation of PPARγ2-MYC induced by the lysosomal inhibitors, NH_4_Cl and bafilomycin A1 (S7F–S7M Fig).

Taken together, these data demonstrate that the GTP-bound form of Rab2A binds to the AF-2 domain of PPARγ and inhibits the proteasomal degradation of PPARγ.

### Nutrition status regulates the activity of the AMPK-TBC1D1-Rab2A axis and subsequently the protein level of PPARγ

**Fig 5:**
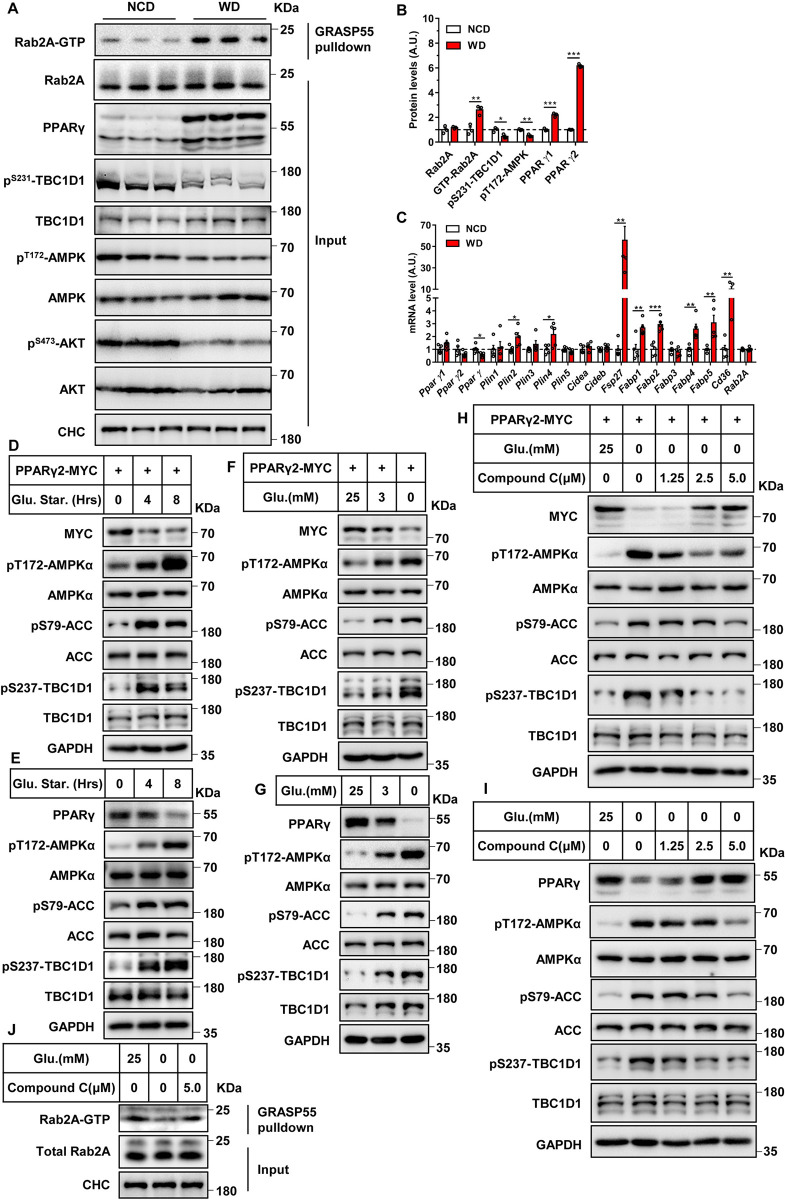
Nutrition levels regulate the activity of AMPK-TBC1D1- Rab2A axis and the subsequent the protein level of PPARγ. **(A)** Q-PCR analysis of liver samples. Male mice were fed western diet for 3 months, and liver samples were then collected and analyzed by Q-PCR (random feed, *n* = 5 per group). **(B)** Immunoblot analysis of liver samples. Male mice were fed western diet for 3 months, and the liver samples were collected and analyzed by immunoblotting (random feed, *n* = 3 per group). **(C)** Quantification of the protein levels shown in B. The data were analyzed with unpaired 2-tailed Student *t* test and are presented as the means ± s.e.m.s. “*” indicates *p* < 0.05, “**” indicates *p* < 0.01, and “***” indicates *p* < 0.001. Raw data are given in S1 Excel spreadsheet with raw data from all figures. **(D–G)** Glucose starvation attenuates the protein level of PPARγ. HEK293T cells (D and F) and HepG2 (E and G) cells were cultured and handled with various hours of glucose depletion (D and E) or various concentrations of glucose (F and G) as methods described. The results were analyzed via immunoblotting. **(H, I)** Inhibition of AMPK signaling with compound C increases the protein level of PPARγ. As described in the methods, HepG2 cells (H) and HEK293T cells (I), were pretreated with various concentrations of inhibitor for 30 minutes and then incubated with the indicated culture medium for 8 hours. The results were analyzed by immunoblotting. **(J)** Glucose starvation decreases the GTP-bound form of Rab2A via the AMPK pathway. HepG2 cells were cultured, stimulated, and harvested for the GST-GRASP55 pulldown assay. The above cellular experiments were performed at least twice with similar results. AMPK, adenosine monophosphate–activated protein kinase.

AMPK is a well-known kinase that senses the energy/nutrient status including cellular glucose levels [26]. Therefore, we investigated how the AMPK-TBC1D1-Rab2A axis regulates PPARγ protein in response to nutrition status. We first utilized a mouse model of western diet-induced obesity (DIO), which develops fatty liver due to overnutrition. Overnutrition in the DIO mice expectedly inactivated AMPK as evidenced by decreased phosphorylation of AMPK, which consequently resulted in lower TBC1D1 phosphorylation in the liver (Fig 5A and 5B). Notably, Rab2A became GTP-loaded active form in the liver of DIO mice (Fig 5A and 5B). Both *Pparγ1* and *Pparγ2* mRNA levels did not change in the liver of DIO mice (Fig 5C), while their protein levels were significantly increased (Fig 5A and 5B). Moreover, PPARγ target genes were also significantly increased in the liver of DIO mice (Fig 5C). These data show that the AMPK-TBC1D1-Rab2A axis responds to overnutrition to increase PPARγ protein in mouse livers.

We next employed cell models of glucose starvation to further investigate how the AMPK-TBC1D1-Rab2A axis regulates PPARγ protein in response to nutrition status. As expected, glucose starvation activated the AMPK pathway in both HEK293T and HepG2 cells as evidenced by the increased phosphorylation of AMPK and ACC, which further phosphorylated TBC1D1 at serine 237 site (Fig 5D–5G). Notably, glucose starvation decreased the protein levels of exogenous PPARγ2-MYC in HEK293T cells (Fig 5D and 5F) and also endogenous PPARγ in HepG2 cells (Fig 5E and 5G). We then used an AMPK inhibitor, compound C, to treat cells that were subjected to glucose starvation. Compound C dose dependently inhibited the AMPK activation induced by glucose starvation, which further led to inhibition of glucose starvation-induced TBC1D1 phosphorylation in both cell types (Fig 5H and 5I). Importantly, Compound C dose dependently restored the protein levels of exogenous PPARγ2-MYC in glucose-starved HEK293T cells (Fig 5H) as well as endogenous PPARγ in glucose-starved HepG2 cells (Fig 5I). Furthermore, glucose depletion lowered the GTP-bound form of Rab2A in HepG2 cells, and compound C reversed this effect (Fig 5J).

Taken together, these data demonstrate that the AMPK-TCB1D1-Rab2A axis regulates PPARγ protein level in response to nutrition status.

### Rab2A regulates cellular lipid accumulation

**Fig 6:**
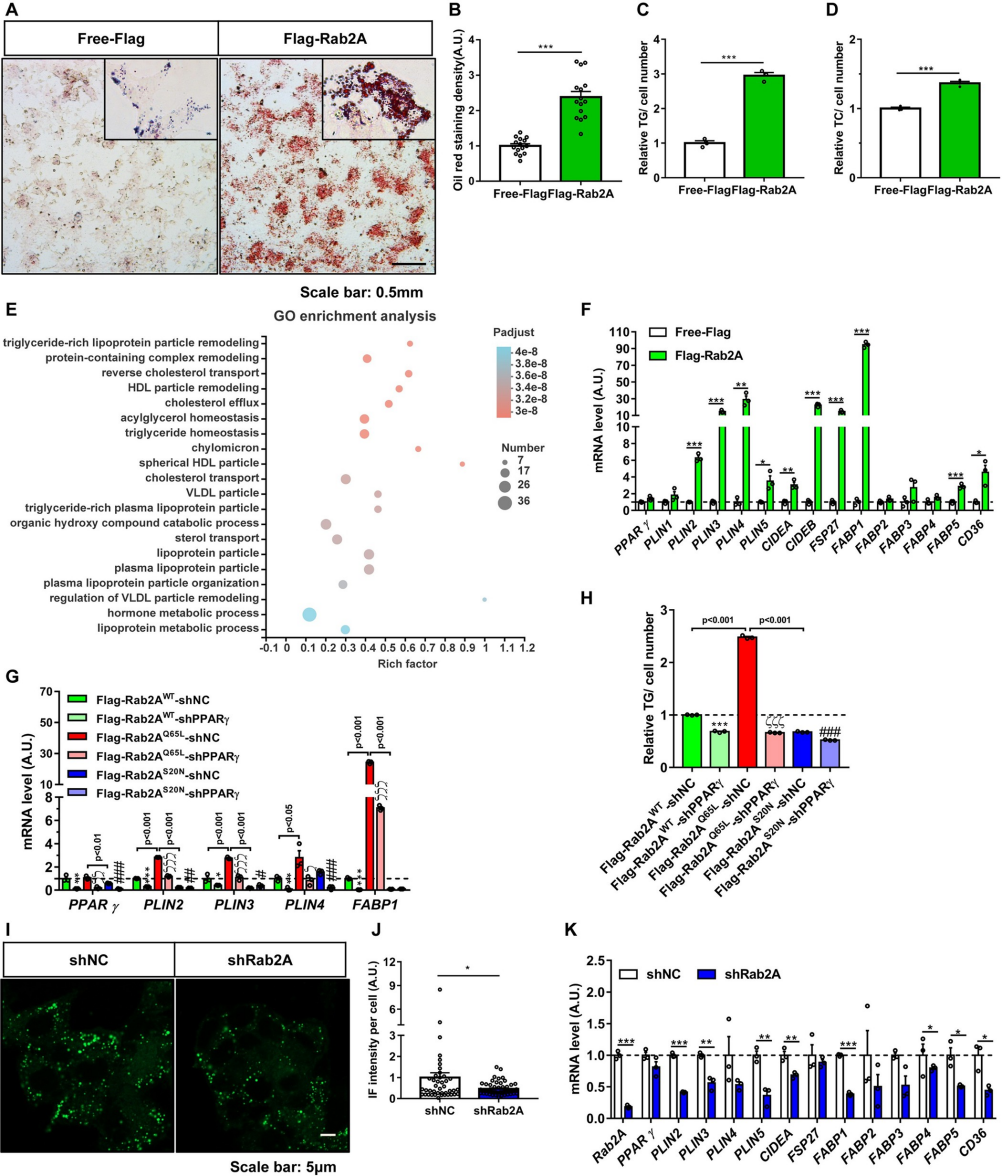
Rab2A regulates cellular lipid accumulation. **(A)** Oil Red O staining of Rab2A-overexpressing and control HepG2 cells. The stable cell lines were fixed and stained with Oil Red O. Representative images are shown. **(B)** Quantification of the staining density in A. The data were analyzed with unpaired 2-tailed Student *t* test and are presented as the means ± s.e.m.s. “***” indicates *p* < 0.001. **(C, D)** Cellular TG levels (C) and TC levels (D) in the Rab2A-overexpressing and control HepG2 cells (*n* = 3 per group). TG and TC levels of control cells were normalized to 1. These data were analyzed with unpaired 2-tailed Student *t* test and are presented as the means ± s.e.m.s. “***” indicates *p* < 0.001. **(E)** GO enrichment analysis based on RNA sequencing data show the major roles of Rab2A in lipid metabolism. Free-Flag versus Flag-Rab2A (*n* = 3 per group). **(F)** Marked increases in PPARγ-targeted genes were observed in Rab2A-overexpressing HepG2 cells. Cell lines with stable Rab2A overexpression were harvested and analyzed by Q-PCR (*n* = 3 per group). The data were analyzed with unpaired 2-tailed Student *t* test and are presented as the means ± s.e.m.s. “*” indicates *p* < 0.05, “**” indicates *p* < 0.01, and “***” indicates *p* < 0.001. **(G, H)** Knockdown of PPARγ rescues the effects of lipogenesis in HepG2 cells overexpressing Rab2A. HepG2 cells were cultured and transfected with the indicated lentivirus-expressing plasmids, and then the positive cells were chosen and harvested for Q-PCR (G) and TG testing (H). The TG level of control cells was normalized to 1, and the statistical data were analyzed with unpaired 2-tailed Student *t* test (*n* = 3 per group) and are presented as the means ± s.e.m.s. shNC versus shPPARγ in Flag-Rab2A^WT^ overexpressing HepG2 cells (“*” indicates *p* < 0.05, “**” indicates *p* < 0.01, and “***” indicates *p* < 0.001). shNC versus shPPARγ in Flag-Rab2A^Q65L^-overexpressing HepG2 cells (“ζ” indicates *p* < 0.05, “ζζ” indicates *p* < 0.01, and “ζζζ” indicates *p* < 0.001). shNC versus shPPARγ in Flag-Rab2A^S20N^-overexpressing HepG2 cells (“#” indicates *p* < 0.05, “##” indicates *p* < 0.01, and “###” indicates *p* < 0.001). **(I)** The BODIPY staining of Rab2A-knockdown and control HepG2 cells. The cells were incubated with BODIPY for 30 minutes and then fixed for imaging. Representative images are shown. **(J)** Quantification of the IF intensity in I. The data were analyzed with unpaired 2-tailed Student *t* test and are presented as the means ± s.e.m.s. “*” indicates *p* < 0.05. **(K)** Marked decreases in PPARγ-targeted genes were found in Rab2A-knockdown HepG2 cells. Cell lines with stable Rab2A knockdown were harvested and analyzed by Q-PCR (*n* = 3 per group). The data were analyzed with unpaired 2-tailed Student *t* test and are presented as the means ± s.e.m.s. “*” indicates *p* < 0.05, “**” indicates *p* < 0.01, and “***” indicates *p* < 0.001. Raw data are given in S1 Excel spreadsheet with raw data from all figures. All the above experiments were performed at least 3 times with similar results except for assay in E (once). GO, Gene Ontology; HDL, high-density lipoprotein; TC, total cholesterol; TG, triglyceride; VLDL, very low-density lipoprotein.

We next investigated whether Rab2A regulates cellular TG storage through PPARγ. Stable overexpression of Rab2A resulted in accumulation of lipid droplets in HepG2 cells as revealed by Oil Red O staining (Fig 6A and 6B). In agreement, cellular TG and total cholesterol (TC) levels were significantly increased in HepG2 cells overexpressing Rab2A as compared to those in control cells (Fig 6C and 6D). To define the functions of Rab2A in an unbiased manner, we then performed an RNA sequencing analysis of HepG2 cells stably overexpressing Rab2A. The Gene Ontology (GO) enrichment analysis revealed that the differentially expressed genes were mainly involved in TG homeostasis and lipoprotein particle remodeling and transport (Fig 6E). Moreover, we found markedly increased transcription levels of PPARγ target genes in HepG2 cells stably overexpressing Rab2A, such as *PLIN4*, *CIDEA*, and *FSP27/CIDEC* (Fig 6F), as similarly seen in the livers of TBC1D1-KI mice (Fig 1E, S2B Fig). The effects of Rab2A on the expression of PPARγ target genes and the cellular TG accumulation were dependent on its guanine nucleotide binding states. The GTP-bound Rab2A^Q65L^, but not the GDP-bound Rab2A^S20N^, promoted the expression of PPARγ target genes (Fig 6G) and caused cellular TG accumulation (Fig 6H). Importantly, knockdown of PPARγ suppressed Rab2A^Q65L^-induced expression of PPARγ target genes (Fig 6G) and prevented Rab2A^Q65L^-elicited cellular TG accumulation (Fig 6H). In contrast to Rab2A overexpression, stable knockdown of Rab2A decreased the amounts of cellular lipid droplets (Fig 6I and 6J) and suppressed mRNA expression of PPARγ target genes (Fig 6K) in HepG2 cells. Together, these data demonstrate that Rab2A regulates lipid storage gene expression and cellular TG accumulation through PPARγ.

### Suppression of Rab2A alleviates diet-induced hepatic lipid accumulation

**Fig 7:**
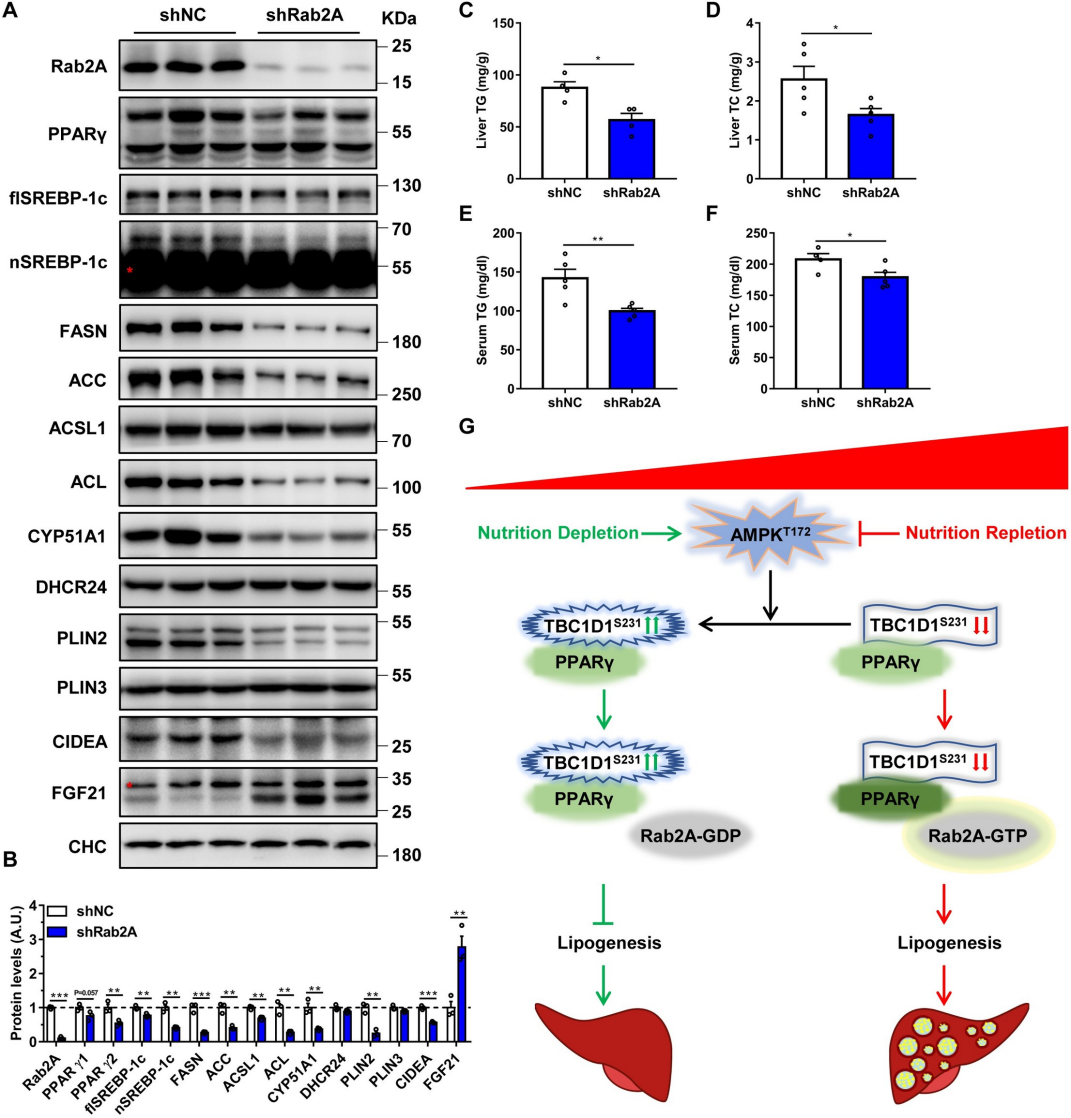
Knockdown of Rab2A improves diet-induced hepatic lipid accumulation. **(A)** Marked decreased protein levels of PPARγ were found in Rab2A-knockdown liver samples. Male mice were intravenously injected with AAV8-Rab2A-shRNA virus and then fed a western diet for 2 months. Liver samples were collected and analyzed by immunoblotting (random feed, *n* = 3 per group). The red asterisk indicates nonspecific signal. **(B)** Quantification of the protein levels shown in A. **(C)** TG levels in the liver of AAV8-Rab2A-shRNA and control mice (random feed, *n* = 5 per group). **(D)** Cholesterol levels in the livers of AAV8-Rab2A-shRNA and control mice (random feed, *n* = 5 per group). **(E)** TG levels in the serum of AAV8-Rab2A-shRNA and control mice (random feed, *n* = 5 per group). **(F)** Cholesterol levels in the serum of AAV8-Rab2A-shRNA and control mice (random feed, *n* = 5 per group). **(G)** Simplified model depicting the regulatory mechanism of PPARγ based on the energy status and the AMPK-TBC1D1-Rab2A axis. Nutrition repletion inactivates the AMPK-TBC1D1 pathway via dephosphorylation, augments the level of GTP-bound Rab2A and then increases the protein stability of PPARγ, which ultimately promotes the progression of NAFLD. Meanwhile, TBC1D1 can also mildly promote the protein stability of PPARγ via constitutive binding independent of phosphorylation at the serine-231 site. These data were analyzed with unpaired 2-tailed Student *t* test and are presented as the means ± s.e.m.s. “*” indicates *p* < 0.05, “**” indicates *p* < 0.01, and “***” indicates *p* < 0.001. Raw data are given in S1 Excel spreadsheet with raw data from all figures. NAFLD, nonalcoholic fatty liver disease; shRNA, short hairpin RNA; TG, triglyceride.

We next sought to find out whether suppression of Rab2A might help to alleviate diet-induced hepatic lipid accumulation in vivo. To address this question, we down-regulated Rab2A expression in the livers of DIO mice through adeno-associated virus serotype 8 (AAV8) mediated expression of shRNA (shRab2A). After the delivery of shRNA-expressing AAV8 for 2 months, the mice were subjected to molecular and physiological analyses. The molecular analysis of mouse livers confirmed a pronounced decrease of Rab2A at both mRNA and protein levels (Fig 7A and 7B, S8A Fig). Notably, endogenous PPARγ2 protein was significantly decreased in the livers of shRab2A mice, and a less pronounced reduction was also observed for endogenous PPARγ1 protein (*p* = 0.057) in these mice, although *Pparγ* mRNA remained normal (Fig 7A and 7B, S8A Fig). These data indicated that the knockdown of Rab2A attenuated the protein stability of PPARγ, which is consistent with the cellular results (Fig 3D, S6C Fig). Besides the decrease of PPARγ protein, we also observed lower expression of genes involved in lipid metabolism, such as SREBP-1c, FASN, ACL, and ACC, at the protein level (Fig 7A and 7B) and mRNA level (S8A and S8B Fig). In addition, the genes involved in the cholesterol synthesis pathway were also inhibited (Fig 7A and 7B, S8C Fig), and this inhibition might be a secondary effect of the suppression of PPARγ. We also detected other genes that are related to fatty acid metabolism or the functions of PPARγ, such as transcription factors (S8D Fig), fatty acid secretion and uptake (S8E Fig), and fatty acid oxidation and lipolysis (S8F Fig), and the results showed that the mRNA levels of most of these genes were normal. Surprisingly, FGF21, a well-known hepatokine, was significantly increased in the livers of shRab2A mice (Fig 7A and 7B, S8D Fig).

We subsequently studied the phenotypes of shRab2A mice, particularly those related to lipid metabolism. The liver weight of the shRab2A mice was significantly lower than that of the control mice (shNC) (S8H Fig), whereas their body weight remained normal (S8G Fig). Given the impacts of Rab2A on PPARγ and lipid metabolism, the change in liver weight might owe to decreases in hepatic TG and cholesterol storage. Indeed, the TG contents were significantly decreased in the livers of shRab2A mice (Fig 7C), which was in agreement with the reduced lipid droplets detected in liver sections (S8I Fig). We also observed a decreased TC level in the livers of shRab2A mice (Fig 7D). Interestingly, both serum TG and TC levels were significantly decreased in the shRab2A mice (Fig 7E and 7F), while the nonesterified fatty acid (NEFA) level was normal in these mice (S8J Fig). In contrast to lipid metabolism, glucose metabolism was not affected in the shRab2A mice, as evidenced by unaltered basal glucose levels (S8K Fig), insulin levels (S8L Fig), glucose tolerance (S8M Fig), and insulin sensitivity (S8N Fig).

Taken together, these data demonstrate that suppression of Rab2A lowers PPARγ protein and thereby alleviates diet-induced hepatic lipid accumulation in vivo.

## Discussion

Our findings reveal a previously unrecognized role of Rab2A in regulation of hepatic TG storage, which functions genetically at the downstream of the AMPK-TBC1D1 axis to regulate PPARγ protein stability and thereby the expression of PPARγ target genes. Our results are consistent with a composite model in which overnutrition attenuates the phosphorylation levels of AMPK-TBC1D1 signaling and augments the GTP-bound active Rab2A, which consequently promotes hepatic steatosis through increasing the protein stability of PPARγ and the expression of PPARγ target genes (Fig 7G).

AMPK has been implicated in whole-body energy/nutrient metabolism via various downstream targets [4,6,27]. Many studies have indicated that the activation of AMPK in the liver partially attenuates high-fat diet-induced fatty liver, but it is not clear whether, and if so, how a reduction in AMPK activity contributes to the development of this disease [28–30]. Here, we show that phosphorylation of AMPK and its downstream target, TBC1D1, is attenuated in the liver of western diet-induced obese mice, which promotes the GTP-bound form of Rab2A and PPARγ protein levels. Our data reveal a previously unknown molecular link between AMPK and PPARγ and strongly demonstrate that genetically blocking the AMPK-TBC1D1 axis aggravates the progression of NAFLD in aged mice. A previous study shows that a choline-deficient high-fat diet (CD-HFD) can also reduce the phosphorylation level of AMPK and demonstrates the important role of the AMPK-Caspase 6 axis in nonalcoholic steatohepatitis [31]. Therefore, AMPK functions as an energy/nutrient sensor, whose inhibition in response to overnutrition contributes to the development of NAFLD at multiple disease stages through distinct downstream targets.

Genetic studies have implicated that TBC1D1 is an important candidate gene of obesity. For example, a human epidemiologic study shows that an R125W coding variant of TBC1D1 confers a risk for familial obesity; however, the underlying molecular mechanism is not clear [32,33]. In mice, whole-body deletion of TBC1D1 confers leanness and protects against obesity induced by a high-fat diet [34,35]. Deficiency of TBC1D1 impairs glucose uptake, but enhances fatty acid uptake and oxidation in skeletal muscle [34,35]. The TBC1D1-Ser231 KI mutation in mice gives rise to obesity and type 2 diabetes through promoting IGF1 secretion and thereby increasing lipogenesis in the adipose tissue [9]. These studies show that TBC1D1 may regulate lipid metabolism in different tissues via distinct mechanisms. Here, we reveal a previously unrecognized role of TBC1D1 in the control of hepatic lipid storage through composite actions involving both direct binding and Rab2A-dependent regulation of PPARγ (Fig 7G), which not only further demonstrates the importance of TBC1D1 in lipid metabolism but also links this protein to another metabolic disease NAFLD.

Our previous study shows that the TBC1D1^S231A^ mutation inhibits its binding to 14-3-3s and increases its GAP activity toward Rab8A, thus resulting in a decrease of GTP-bound Rab8A [9]. The increased GAP activity by the TBC1D1^S231A^ mutation causes a partial inhibition of AICAR-stimulated GLUT4 transport and glucose uptake in skeletal muscle presumably through decreasing the GTP-bound form of a yet unknown Rab small G protein [7]. In this study, we find that TBC1D1^S231A^ mutation and TBC1D1-Ser231 hypophosphorylation through inhibition of AMPK both result in an increase of the GTP-bound active Rab2A, suggesting that Rab2A might not be a direct substrate of TBC1D1 in vivo (Fig 3E–3G). Small G proteins on a pathway may be networked to one another via their GAPs or guanine nucleotide exchange factors (GEFs) to form signaling cascades [36]. It is possible that TBC1D1 might regulate a Rab protein that, in turn, controls some GAPs or GEFs for Rab2A. Alternatively, TBC1D1 might have crosstalk with other pathways to indirectly regulate Rab2A activation. A third possibility is that Ser231 dephosphorylation might relocate TBC1D1 away from Rab2A thereby allowing a Rab-GEF to increase the GTP-loaded form of Rab2A. Nevertheless, our genetic analysis demonstrates that Rab2A functions genetically at the downstream of TBC1D1 to regulate PPARγ protein stability. Both TBC1D1 and Rab2A interact with PPARγ and may regulate PPARγ protein stability in a cooperative manner.

The functions of Rab GTPases in lipid droplet metabolism, such as Rab8A [37] and Rab18 [38,39], have been discovered but have not yet been implicated in the progression of NAFLD. The results from this study reveal the novel functions of Rab2A regulated by the AMPK-TBC1D1 axis in hepatic steatosis. Previous studies have demonstrated that Rab2A is localized at the Golgi apparatus and that this protein is essential for protein transport [24]. It also plays a critical role in regulation of the formation of autophagosomes [40]. Whole-body deletion of Rab2A leads to preweaning lethality in mice, thus preventing the utilization of this model in studying its functions in the adulthood [22]. Therefore, liver-specific Rab2A knockout models are needed to further delineate the in vivo functions of Rab2A in regulation of hepatic lipid metabolism and in the pathogenesis of NAFLD in the future.

It is intriguing that the protein levels of PPARγ, including PPARγ1 and PPARγ2, in the livers of mice with western DIO are significantly higher than those in the livers of mice with normal chow diet, while the *Pparγ* mRNA level is comparable between the 2 groups of mice. This is in contrast to previous reports in which the *Pparγ* transcripts are increased in the liver of high-fat diet-fed mice or leptin-deficient mice [12,13,41]. In our study, the GTP-bound form of Rab2A regulates the stability of PPARγ mainly by interacting with the AF-2 domain to prevent its proteasomal degradation. The AF-2 domain is responsible for ligand binding and has been shown to be critical for ligand-induced degradation [42]. It is possible that Rab2A may play a critical role in the ligand-induced PPARγ degradation. Future studies are required to elucidate the molecular mechanisms through which Rab2A binds to PPARγ, the components involved in this process, and the relationship with ligand binding. Recently, researchers have demonstrated that the serine 273 site of PPARγ is linked to obesity and insulin resistance [11], and other studies have shown that the phosphorylation of S273 can inversely reduce the total protein level of PPARγ in white adipose tissue through a yet unknown mechanism [16,43]. Given that PPARγ is primarily expressed in the adipose tissue, it is intriguing to find out whether the mechanism regulating hepatic PPARγ stability might also play a role in regulating adipose PPARγ protein.

In summary, we show that Rab2A functions genetically at the downstream of the AMPK-TBC1D1 axis to regulate hepatic PPARγ protein stability and thereby the expression of PPARγ target genes for TG accumulation in the liver in response to energy/nutrient status. Our findings may have therapeutic implications for treatment of fatty liver disease.

## Materials and methods

All mice were housed in a pathogen-free environment with a 12-hour light/12-hour dark cycle and had free access to water consumption and food intake. All animal breeding, husbandry, care, euthanasia, and use procedures followed the guidelines provided by the Ethics Committees of Nanjing University (Approval number MARC-CS3) and Anhui Medical University (Approval number LLSC20200327). Extended methods and information about reagents, cell culture, TBC1D1^S231A^-KI mouse model, DIO mouse model, AAV8-mediated Rab2A gene knockdown in the liver, transfection and plasmids, immunoblotting and antibodies, glucose starvation, immunoprecipitation, RNA isolation and quantitative PCR, RNA sequencing, prokaryotic expression and purification, pull down assay of Rab2A-GTP, cytoplasmic and nuclear extraction, immunofluorescence staining and imaging, Oil Red O staining and imaging, histology and imaging, blood chemistry, measurement of liver and cellular TC and TG levels, oral glucose tolerance test and insulin tolerance test, and statistical analysis are described in S1 Supplemental Materials and Methods. The original RNA sequencing data are listed in S1 RNA Sequencing data. The raw data of statistical results are given in S1 Excel spreadsheet with raw data from all figures and raw western blot images are shown in S1 Raw images.

## Supporting information

S1 Fig Hepatic lipid metabolism in aged TBC1D1^S231A^ mice. **(A)** Hematoxylin–eosin staining of liver sections from WT and TBC1D1-KI male mice aged 4 to 6 months (random feed, *n* = 5 per group). Representative images are shown. **(B)** TG levels in the livers of WT and TBC1D1 KI male mice aged 4 to 6 months (random feed, *n* = 5 per group). **(C)** Hematoxylin–eosin staining of liver sections from WT and TBC1D1-KI male mice aged 12 months (random feed, *n* = 4 per group). Representative images are shown. **(D)** TG levels in the livers of WT and TBC1D1-KI male mice aged 12 months (random feed, *n* = 4 per group). **(E–H)** mRNA expression levels of genes in liver samples from TBC1D1-KI mice aged 18 months. The levels of genes related to lipolysis (E), fatty acid uptake and secretion (F), transcription factors (G), and fatty acid synthesis (H) were determined by Q-PCR (random feed, *n* = 4 per group). **(I)** Immunoblotting analysis of liver samples from WT and TBC1D1-KI mice aged 18 months (random feed, *n* = 4 per group). **(J)** Statistical analysis of the protein levels shown in I. **(K)** Immunoblotting analysis of liver samples from WT and TBC1D1-KI mice aged 12 months (random feed, *n* = 3 per group). **(L)** Statistical analysis of the protein levels shown in K. These data were analyzed with unpaired 2-tailed Student *t* test and are presented as the means ± s.e.m.s. “*” indicates *p* < 0.05, and “**” indicates *p* < 0.01. Raw data are given in S1 Excel spreadsheet with raw data from all figures. TG, triglyceride; WT, wild-type.
(TIF)

S2 Fig Activation of PPARγ signaling in the livers of aged TBC1D1^S231A^ mice. **(A)** The KEGG enrichment analysis based on RNA sequencing, data show significantly different pathways in the livers of WT and TBC1D1-KI male mice aged 18 months (random feed, *n* = 3 per group). **(B)** mRNA expression levels of genes in heatmap (Fig 1E) were confirmed by Q-PCR analysis of liver samples from WT and TBC1D1-KI male mice aged 12 months (random feed, *n* = 4 per group). **(C)** Increased protein level of PPARγ in the liver of WT and TBC1D1-KI mice aged 12 months. The data were obtained by immunoblotting (random feed, *n* = 3 per group). **(D)** Statistical analysis of the protein levels in C. **(E)** mRNA expression levels of PPARγ target genes in the livers of WT and TBC1D1-KI mice aged 12 months were confirmed by Q-PCR (random feed, *n* = 4 per group). The data were analyzed with unpaired 2-tailed Student *t* test and are presented as the means ± s.e.m.s. “*” indicates *p* < 0.05, “**” indicates *p* < 0.01, and “***” indicates *p* < 0.001. Raw data are given in S1 Excel spreadsheet with raw data from all figures. WT, wild-type.
(TIF)

S3 Fig TBC1D1 regulates the protein stability of PPARγ. **(A)** Overexpression of TBC1D1 increases the protein stability of endogenous PPARγ. HepG2 cells were cultured and transfected with the indicated plasmids, and 2 days later, the cells were collected and analyzed by immunoblotting. **(B–D)** Overexpression of TBC1D1 increases the protein stability of exogenous PPARγ1. HepG2 (B) and HEK293T (C and D) cells were cultured and transfected with the indicated plasmids for 2 days, and the cells were collected and analyzed by immunoblotting. **(E–G)** Overexpression of TBC1D1 increases the protein stability of exogenous PPARγ2. HepG2 (E) and HEK293T (F and G) cells were cultured and transfected with the indicated plasmids for 2 days, and the cells were collected and analyzed by immunoblotting. **(H)** Gradient overexpression of TBC1D1^S237A^ plasmids increases the protein stability of exogenous PPARγ2. HEK293T cells were cultured and transfected with the indicated plasmids, and 2 days later, the cells were collected and analyzed by immunoblotting. **(I)** Blocking the phosphorylation of TBC1D1 at serine 237 increases endogenous protein levels of PPARγ. HepG2 cells were cultured, transfected with the indicated plasmids for 2 days, harvested and analyzed by immunoblotting. **(J)** Blocking the phosphorylation of TBC1D1 at serine 237 increases the activation of PPARγ. TBC1D1^WT^-and TBC1D1^S237A^-overexpressing HepG2 cells were harvested and analyzed by Q-PCR (*n* = 3 per group). The data were analyzed with unpaired 2-tailed Student *t* test and are presented as the means ± s.e.m.s. Free-Flag versus Flag-TBC1D1^WT^ (“*” indicates *p* < 0.05, “**” indicates *p* < 0.01, and “***” indicates *p* < 0.001). Free-Flag versus Flag-TBC1D1^S237A^ (“ζ” indicates *p* < 0.05, “ζζ” indicates *p* < 0.01, and “ζζζ” indicates *p* < 0.001). Flag-TBC1D1^WT^ versus Flag-TBC1D1^S237A^ (“#” indicates *p* < 0.05, “##” indicates *p* < 0.01, and “###” indicates *p* < 0.001). **(K)** Binding assay between WT TBC1D1 and exogenous PPARγ2. HEK293T cells were transfected with Flag-TBC1D1 and PPARγ2-MYC plasmids, harvested, and immunoprecipitated with anti-Flag beads and analyzed by immunoblotting. **(L)** Binding assay between WT TBC1D1 and endogenous PPARγ. HepG2 cells stably expressing Flag-TBC1D1 were harvested and immunoprecipitated with anti-Flag beads and analyzed by immunoblotting. **(M, N)** Overexpression of WT TBC1D1 attenuated the degradation of PPARγ2. HEK293T cells were cultured and transfected with the indicated plasmids, and 2 days later, the cells were stimulated with 200-μM CHX for the indicated hours. The cells were then harvested and analyzed by immunoblotting; the MYC blots were spliced to obtain a similar baseline protein level (M). (N) Quantification of PPARγ2 levels in C. The ratios in lanes 1 and 7 were defined as 1, respectively. Raw data are given in S1 Excel spreadsheet with raw data from all figures. All experiments were performed at least twice with similar results. CHX, cycloheximide; WT, wild-type.
(TIF)

S4 Fig Screening of Rabs that mediate the regulation of PPARγ2. **(A–L)** Rabs, such as Rab2A (A), Rab2B (B), Rab8A (C), Rab8B (D), Rab10 (E), Rab14 (F), Rab1A (G), Rab24 (H), Rab35 (I), Rab7A (J), Rab15 (K), and Rab40A (L), mediate the protein stability of exogenous PPARγ2 after gradient overexpression of different plasmids. HEK293T cells were cultured and transfected with the indicated plasmids, and 2 days later, the cells were collected and analyzed by immunoblotting. All experiments were performed at least twice with similar results.
(TIF)

S5 Fig Screening of Rabs that mediate the regulation of PPARγ2. **(A–K)** Rabs, such as Rab1B (A), Rab5A (B), Rab9A (C), Rab9B (D), Rab11B (E), Rab22B (F), Rab32 (G), Rab4B (H), Rab39A (I), Rab13 (J), and Rab23 (K), mediate the protein stability of exogenous PPARγ2 after gradient overexpression of different plasmids. HEK293T cells were cultured and transfected with the indicated plasmids, and 2 days later, the cells were collected and analyzed by immunoblotting. **(L)** Overexpression of Rab2A increases the protein stability of cytoplasmic and nuclear localized PPARγ2. HEK293T cells were cultured and transfected with the indicated plasmids, and 2 days later, the cells were collected, handled according to the standard protocol and analyzed by immunoblotting. All experiments were performed at least twice with similar results.
(TIF)

S6 Fig Synergistic regulation on PPARγ by TBC1D1 and Rab2A. **(A)** GRASP55, as a marker, specifically interacts with the GTP-bound form of Rab2A. HEK293T cells were cultured, and transfected with the indicated plasmids for 2 days, lysed, and analyzed by GST-GRASP55 pulldown and immunoblotting assays. **(B)** Parallel regulation of PPARγ2 by Rab2A and TBC1D1-WT protein. HEK293T cells were cultured and transfected with a dose curve of TBC1D1-WT protein combined with Rab2A overexpression or not. The cells were collected and analyzed by immunoblotting. The level of MYC was quantified and normalized with lane 1. **(C)** Knockdown of Rab2A attenuates the function of TBC1D1 in the regulation of endogenous PPARγ stability. HepG2 cells were cultured and transfected with lentivirus-expressing plasmids, and then the positive cells were screened, harvested, and analyzed by immunoblotting. The level of PPARγ was quantified and normalized to lane 1. **(D, E)** Knockdown of Rab2A partially rescues the protein level of PPARγ2 underlying TBC1D1-WT or TBC1D1-S237A mutation overexpression. HEK293T cells were cultured, and transfected with a dose curve of TBC1D1-WT protein (D) or TBC1D1-S237A protein (E) combined with Rab2A knockdown or not. The cells were collected and analyzed by immunoblotting. The level of MYC was quantified and normalized to lane 1. All experiments were performed at least 3 times with similar results in addition to the assay in A (twice). WT, wild-type.
(TIF)

S7 Fig Rab2A regulates the degradation of PPARγ. **(A)** The GTP-bound form of Rab2A increases the protein stability of exogenous PPARγ2. HEK293T cells were cultured and transfected with the indicated plasmids for 2 days, and the cells were then harvested and analyzed by immunoblotting. **(B)** Simplified models of different truncated plasmids of PPARγ2. **(C)** Mapping the detailed fragment of PPARγ2 regulated by Rab2A. HEK293T cells were cultured and transfected with the indicated plasmids for 2 days, and the cells were then harvested and analyzed by immunoblotting. **(D, E)** Overexpression of Rab2A attenuated the degradation of PPARγ2. HEK293T cells were cultured and transfected with the indicated plasmids, and 2 days later, the cells were stimulated with 200-μM CHX for the indicated hours. The cells were then harvested and analyzed by immunoblotting; the MYC blots were spliced to obtain a similar baseline protein level (D). (E) Quantification of PPARγ2 levels in D. The ratios in lanes 1 and 7 were defined as 1, respectively. **(F–M)** Rab2A does not regulate the lysosomal degradation of PPARγ2. HEK293T cells were cultured and transfected with the indicated plasmids for 2 days, and the cells were then stimulated with various concentrations of NH_4_Cl (F), bafilomycin A1 (J) or various times of NH_4_Cl (H), bafilomycin A1 (L). The cells were harvested and analyzed by immunoblotting. (G) Quantification of PPARγ2 levels in F. (I) Quantification of PPARγ2 levels in H. (K) Quantification of PPARγ2 levels in J. (M) Quantification of PPARγ2 level in L. The ratio in lane 1 was defined as 1. Raw data are given in S1 Excel spreadsheet with raw data from all figures. All the above experiments were performed at least twice with similar results. CHX, cycloheximide.
(TIF)

S8 Fig Metabolic parameters in the Rab2A liver-specific-knockdown mice. **(A–F)** Quantification of mRNA levels in Rab2A-knockdown and control liver samples. Male mice were intravenously injected with AAV8-Rab2A-shRNA virus and then fed a western diet for 2 months. The liver samples were analyzed by Q-PCR, and this analysis mostly focused on genes related to lipogenesis (A), lipid droplets (B), cholesterol synthesis (C), transcription factors (D), lipoprotein uptake and secretion (E), and lipolysis (F) (random feed, *n* = 5 per group). **(G)** Body weight of AAV8-Rab2A-shRNA and control mice (random feed, *n* = 5 per group). **(H)** Tissue weights of AAV8-Rab2A-shRNA and control mice (random feed, *n* = 5 per group). **(I)** Hematoxylin–eosin staining of liver sections from AAV8-Rab2A-shRNA and control mice (random feed, *n* = 5 per group). Representative images are shown. **(J)** NEFA levels in the serum of AAV8-Rab2A-shRNA and control mice (random feed, *n* = 5 per group). **(K)** Basal glucose level in the blood of AAV8-Rab2A-shRNA and control mice (overnight fast, *n* = 5 per group). **(L)** Insulin level in the serum of AAV8-Rab2A-shRNA and control mice (random feed, *n* = 5 per group). **(M)** OGTT of AAV8-Rab2A-shRNA and control mice (*n* = 5 per group). **(N)** ITT of AAV8-Rab2A-shRNA and control mice (*n* = 5 per group). The data were analyzed with unpaired 2-tailed Student *t* test and are presented as the means ± s.e.m.s. “*” indicates *p* < 0.05, “**” indicates *p* < 0.01, and “***” indicates *p* < 0.001. Raw data are given in S1 Excel spreadsheet with raw data from all figures. AAV8, adeno-associated virus serotype 8; EDL, extensor digitorum longus; epWAT, epididymis white adipose tissue; ITT, insulin tolerance test; NEFA, nonesterified fatty acid; OGTT, Oral glucose tolerance test; prWAT, perirenal white adipose tissue; scWAT, subcutaneous white adipose tissue; shRNA, short hairpin RNA; TA, tibialis anterior muscle.
(TIF)

S1 Supplemental Materials and Methods Extended reagents, materials and methods with detail description. (DOCX)

S1 RNA Sequencing data Original RNA sequencing data. (XLSX)

S1 Excel spreadsheet with raw data from all figures The raw data of statistical results. (XLSX)

S1 Raw images Raw western blot data. (PDF)

## Abbreviations

AAV8: adeno-associated virus serotype 8
ACC: acetyl-CoA carboxylase
ACS1: cytoplasmic acetyl-CoA synthase
AF-2: activation function 2
AMP: adenosine monophosphate
AMPK: adenosine monophosphate–activated protein kinase
CD-HFD: choline-deficient high-fat diet
CHX: cycloheximide
DBD: DNA-binding domain
DIO: diet-induced obesity
FASN: fatty acid synthase
GAP: GTPase-activating protein
GEF: guanine nucleotide exchange factor
GO: Gene Ontology
IGF1: insulin-like growth factor 1
KI: knock-in
LBD: ligand-binding domain
MBOAT7: membrane-bound O-acyltransferase domain–containing 7
NAFLD: nonalcoholic fatty liver disease
NEFA: nonesterified fatty acid
NTR: N terminal region
PNPLA3: patatin-like phospholipase domain–containing 3
RabGAP: Rab-GTPase activating protein
shRNA: short hairpin RNA
TC: total cholesterol
TG: triglyceride
TM6SF2: transmembrane 6 superfamily member 2
WT: wild-type
