# Supplementary figures and images for: Rab2A regulates the progression of nonalcoholic fatty liver disease downstream of AMPK-TBC1D1 axis by stabilizing PPARγ
Source: PLoS Biol. 2022 Jan 21;20(1):e3001522. doi: 10.1371/journal.pbio.3001522 (PMC8809606; doi:10.1371/journal.pbio.3001522)

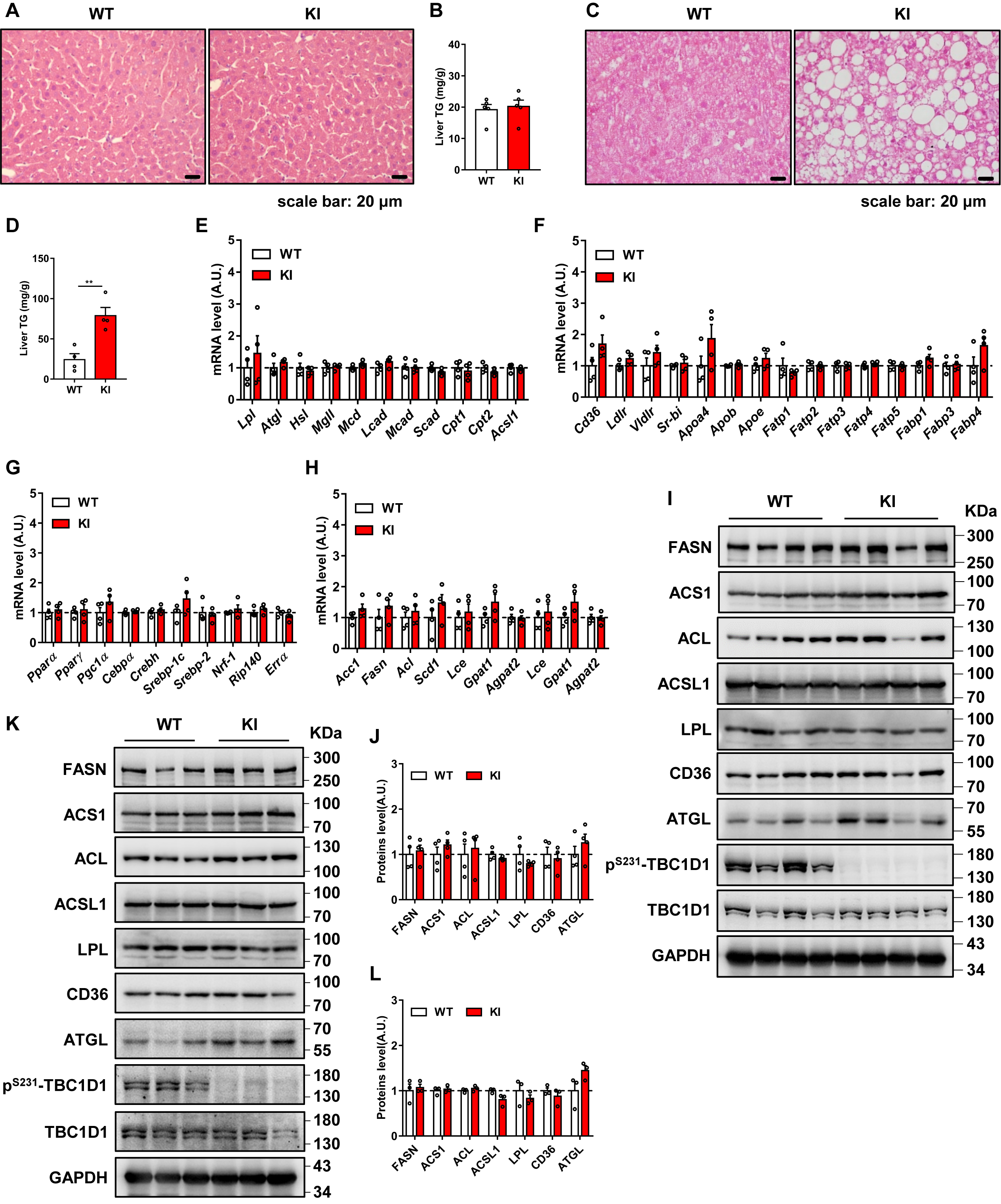

Supplement: S1 Fig — (A) Hematoxylin–eosin staining of liver sections from WT and TBC1D1-KI male mice aged 4 to 6 months (random feed, n = 5 per group). Representative images are shown. (B) TG levels in the livers of WT and TBC1D1 KI male mice aged 4 to 6 months (random feed, n = 5 per group). (C) Hematoxylin–eosin staining of liver sections from WT and TBC1D1-KI male mice aged 12 months (random feed, n = 4 per group). Representative images are shown. (D) TG levels in the livers of WT and TBC1D1-KI male mice aged 12 months (random feed, n = 4 per group). (E–H) mRNA expression levels of genes in liver samples from TBC1D1-KI mice aged 18 months. The levels of genes related to lipolysis (E), fatty acid uptake and secretion (F), transcription factors (G), and fatty acid synthesis (H) were determined by Q-PCR (random feed, n = 4 per group). (I) Immunoblotting analysis of liver samples from WT and TBC1D1-KI mice aged 18 months (random feed, n = 4 per group). (J) Statistical analysis of the protein levels shown in I. (K) Immunoblotting analysis of liver samples from WT and TBC1D1-KI mice aged 12 months (random feed, n = 3 per group). (L) Statistical analysis of the protein levels shown in K. These data were analyzed with unpaired 2-tailed Student t test and are presented as the means ± s.e.m.s. “*” indicates p < 0.05, and “**” indicates p < 0.01. Raw data are given in S1 Excel spreadsheet with raw data from all figures. TG, triglyceride; WT, wild-type. (TIF) [file pbio.3001522.s001.tif]

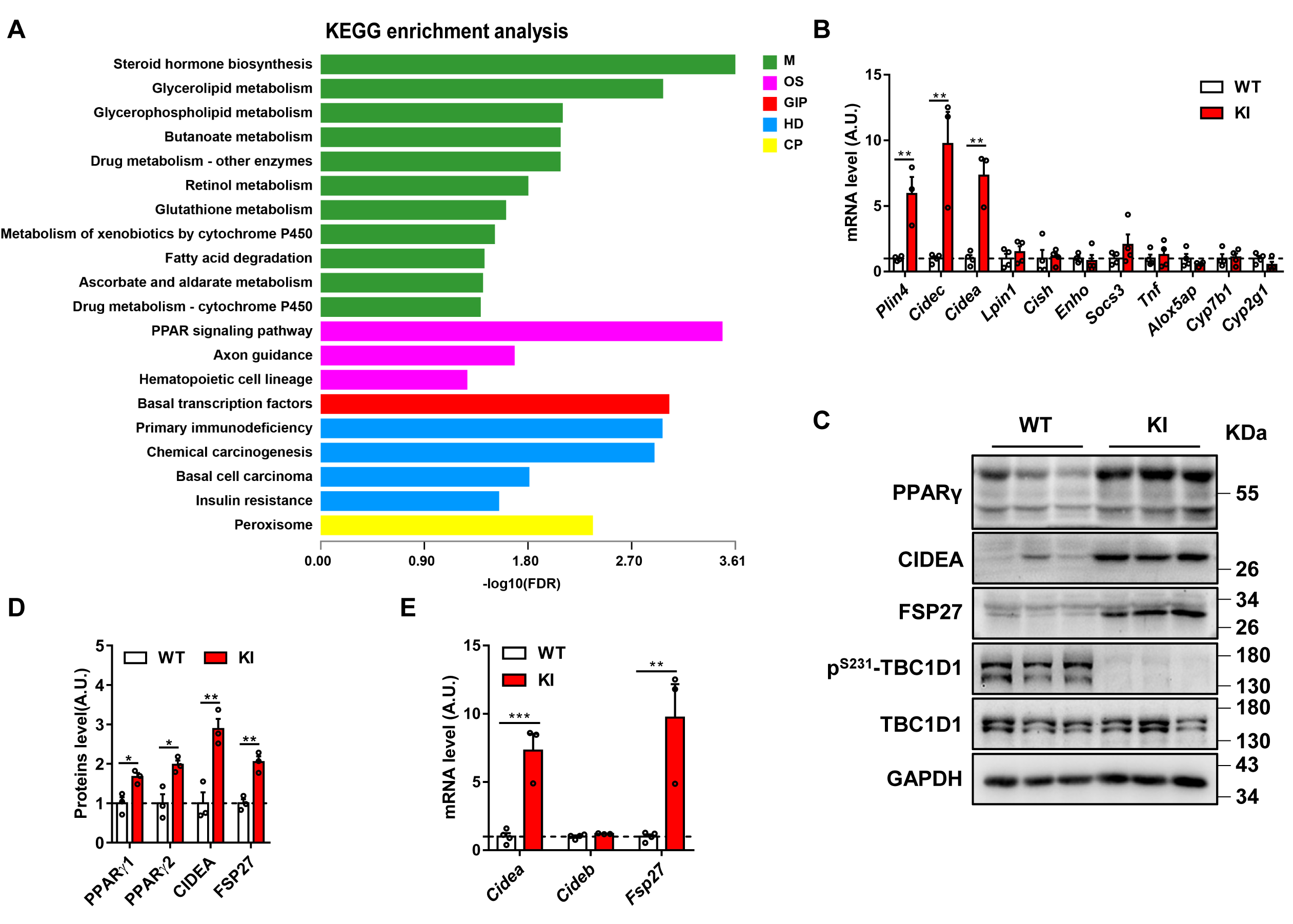

Supplement: S2 Fig — (A) The KEGG enrichment analysis based on RNA sequencing, data show significantly different pathways in the livers of WT and TBC1D1-KI male mice aged 18 months (random feed, n = 3 per group). (B) mRNA expression levels of genes in heatmap (Fig 1E) were confirmed by Q-PCR analysis of liver samples from WT and TBC1D1-KI male mice aged 12 months (random feed, n = 4 per group). (C) Increased protein level of PPARγ in the liver of WT and TBC1D1-KI mice aged 12 months. The data were obtained by immunoblotting (random feed, n = 3 per group). (D) Statistical analysis of the protein levels in C. (E) mRNA expression levels of PPARγ target genes in the livers of WT and TBC1D1-KI mice aged 12 months were confirmed by Q-PCR (random feed, n = 4 per group). The data were analyzed with unpaired 2-tailed Student t test and are presented as the means ± s.e.m.s. “*” indicates p < 0.05, “**” indicates p < 0.01, and “***” indicates p < 0.001. Raw data are given in S1 Excel spreadsheet with raw data from all figures. WT, wild-type. (TIF) [file pbio.3001522.s002.tif]

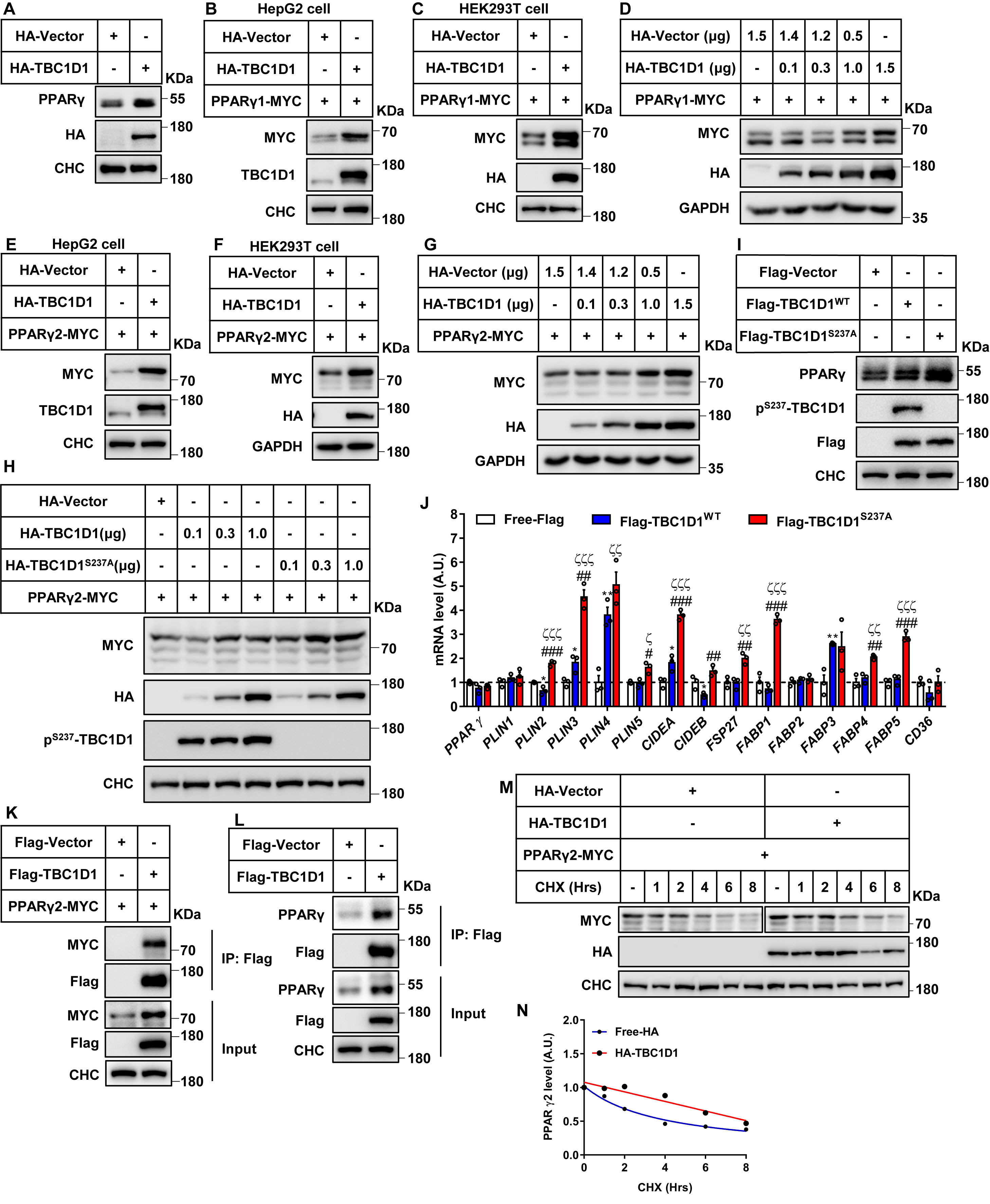

Supplement: S3 Fig — (A) Overexpression of TBC1D1 increases the protein stability of endogenous PPARγ. HepG2 cells were cultured and transfected with the indicated plasmids, and 2 days later, the cells were collected and analyzed by immunoblotting. (B–D) Overexpression of TBC1D1 increases the protein stability of exogenous PPARγ1. HepG2 (B) and HEK293T (C and D) cells were cultured and transfected with the indicated plasmids for 2 days, and the cells were collected and analyzed by immunoblotting. (E–G) Overexpression of TBC1D1 increases the protein stability of exogenous PPARγ2. HepG2 (E) and HEK293T (F and G) cells were cultured and transfected with the indicated plasmids for 2 days, and the cells were collected and analyzed by immunoblotting. (H) Gradient overexpression of TBC1D1S237A plasmids increases the protein stability of exogenous PPARγ2. HEK293T cells were cultured and transfected with the indicated plasmids, and 2 days later, the cells were collected and analyzed by immunoblotting. (I) Blocking the phosphorylation of TBC1D1 at serine 237 increases endogenous protein levels of PPARγ. HepG2 cells were cultured, transfected with the indicated plasmids for 2 days, harvested and analyzed by immunoblotting. (J) Blocking the phosphorylation of TBC1D1 at serine 237 increases the activation of PPARγ. TBC1D1WT-and TBC1D1S237A-overexpressing HepG2 cells were harvested and analyzed by Q-PCR (n = 3 per group). The data were analyzed with unpaired 2-tailed Student t test and are presented as the means ± s.e.m.s. Free-Flag versus Flag-TBC1D1WT (“*” indicates p < 0.05, “**” indicates p < 0.01, and “***” indicates p < 0.001). Free-Flag versus Flag-TBC1D1S237A (“ζ” indicates p < 0.05, “ζζ” indicates p < 0.01, and “ζζζ” indicates p < 0.001). Flag-TBC1D1WT versus Flag-TBC1D1S237A (“#” indicates p < 0.05, “##” indicates p < 0.01, and “###” indicates p < 0.001). (K) Binding assay between WT TBC1D1 and exogenous PPARγ2. HEK293T cells were transfected with Flag-TBC1D1 and PPARγ2-MYC plasmids, harves [file pbio.3001522.s003.tif]

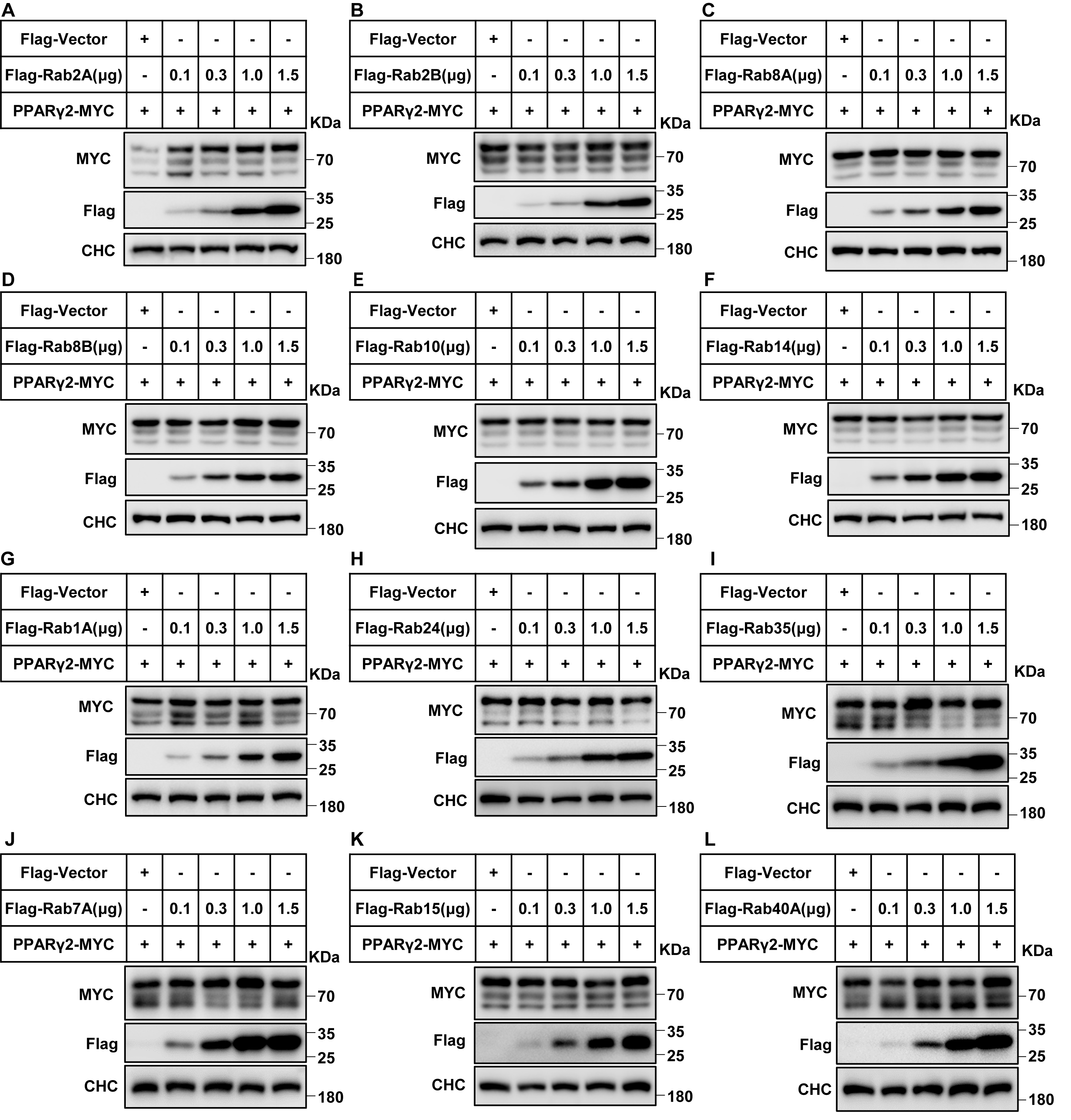

Supplement: S4 Fig — (A–L) Rabs, such as Rab2A (A), Rab2B (B), Rab8A (C), Rab8B (D), Rab10 (E), Rab14 (F), Rab1A (G), Rab24 (H), Rab35 (I), Rab7A (J), Rab15 (K), and Rab40A (L), mediate the protein stability of exogenous PPARγ2 after gradient overexpression of different plasmids. HEK293T cells were cultured and transfected with the indicated plasmids, and 2 days later, the cells were collected and analyzed by immunoblotting. All experiments were performed at least twice with similar results. (TIF) [file pbio.3001522.s004.tif]

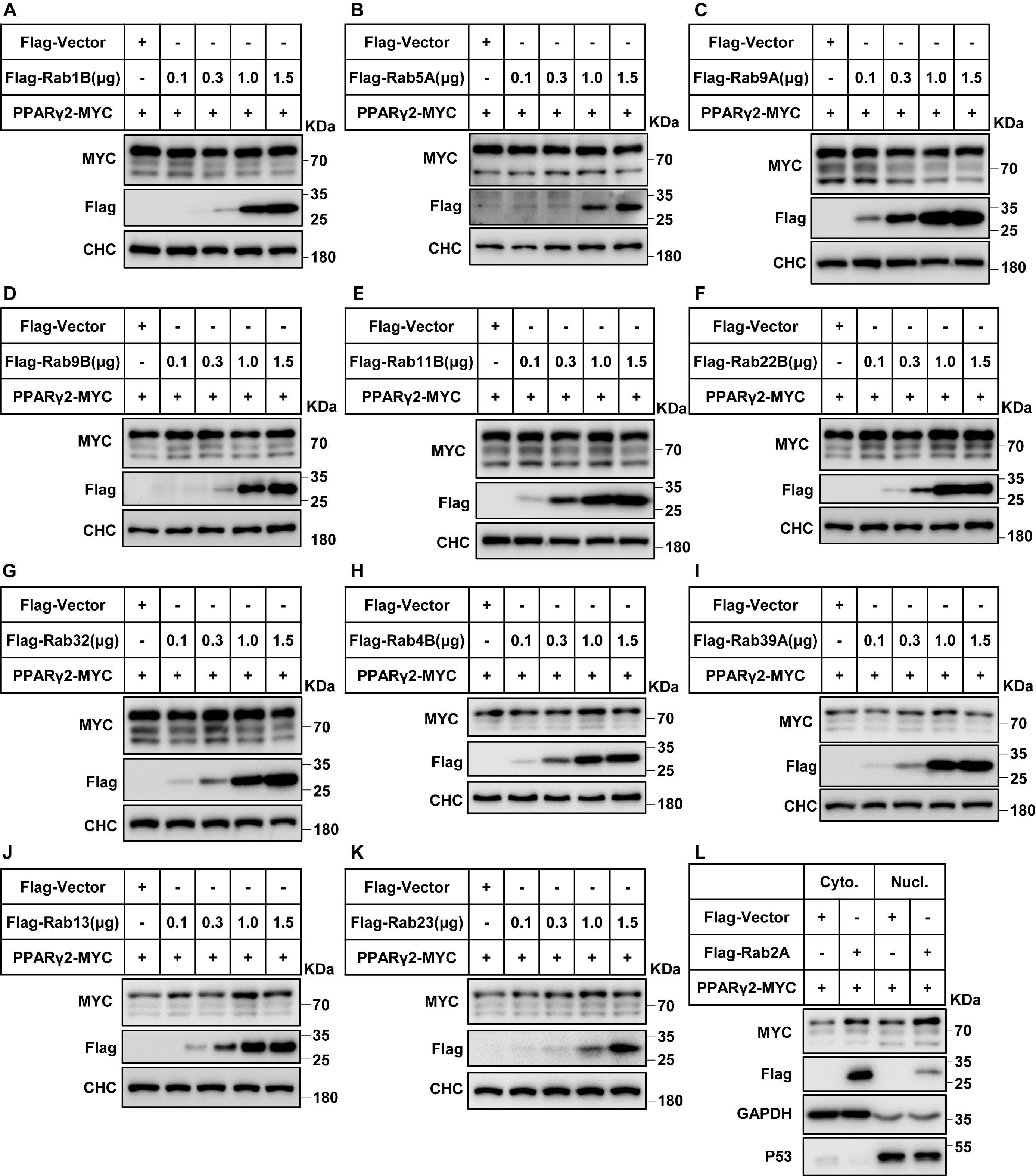

Supplement: S5 Fig — (A–K) Rabs, such as Rab1B (A), Rab5A (B), Rab9A (C), Rab9B (D), Rab11B (E), Rab22B (F), Rab32 (G), Rab4B (H), Rab39A (I), Rab13 (J), and Rab23 (K), mediate the protein stability of exogenous PPARγ2 after gradient overexpression of different plasmids. HEK293T cells were cultured and transfected with the indicated plasmids, and 2 days later, the cells were collected and analyzed by immunoblotting. (L) Overexpression of Rab2A increases the protein stability of cytoplasmic and nuclear localized PPARγ2. HEK293T cells were cultured and transfected with the indicated plasmids, and 2 days later, the cells were collected, handled according to the standard protocol and analyzed by immunoblotting. All experiments were performed at least twice with similar results. (TIF) [file pbio.3001522.s005.tif]

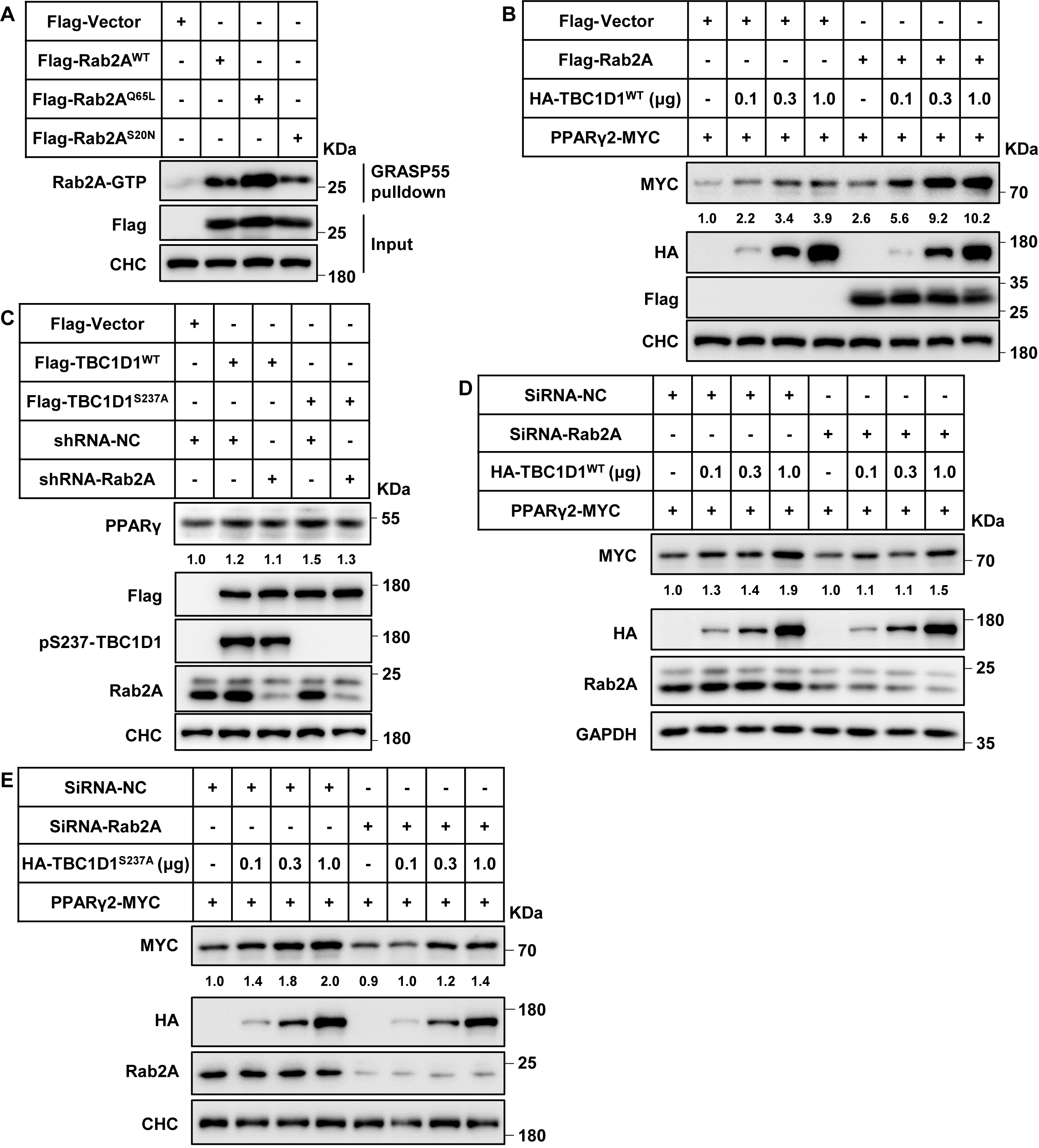

Supplement: S6 Fig — (A) GRASP55, as a marker, specifically interacts with the GTP-bound form of Rab2A. HEK293T cells were cultured, and transfected with the indicated plasmids for 2 days, lysed, and analyzed by GST-GRASP55 pulldown and immunoblotting assays. (B) Parallel regulation of PPARγ2 by Rab2A and TBC1D1-WT protein. HEK293T cells were cultured and transfected with a dose curve of TBC1D1-WT protein combined with Rab2A overexpression or not. The cells were collected and analyzed by immunoblotting. The level of MYC was quantified and normalized with lane 1. (C) Knockdown of Rab2A attenuates the function of TBC1D1 in the regulation of endogenous PPARγ stability. HepG2 cells were cultured and transfected with lentivirus-expressing plasmids, and then the positive cells were screened, harvested, and analyzed by immunoblotting. The level of PPARγ was quantified and normalized to lane 1. (D, E) Knockdown of Rab2A partially rescues the protein level of PPARγ2 underlying TBC1D1-WT or TBC1D1-S237A mutation overexpression. HEK293T cells were cultured, and transfected with a dose curve of TBC1D1-WT protein (D) or TBC1D1-S237A protein (E) combined with Rab2A knockdown or not. The cells were collected and analyzed by immunoblotting. The level of MYC was quantified and normalized to lane 1. All experiments were performed at least 3 times with similar results in addition to the assay in A (twice). WT, wild-type. (TIF) [file pbio.3001522.s006.tif]

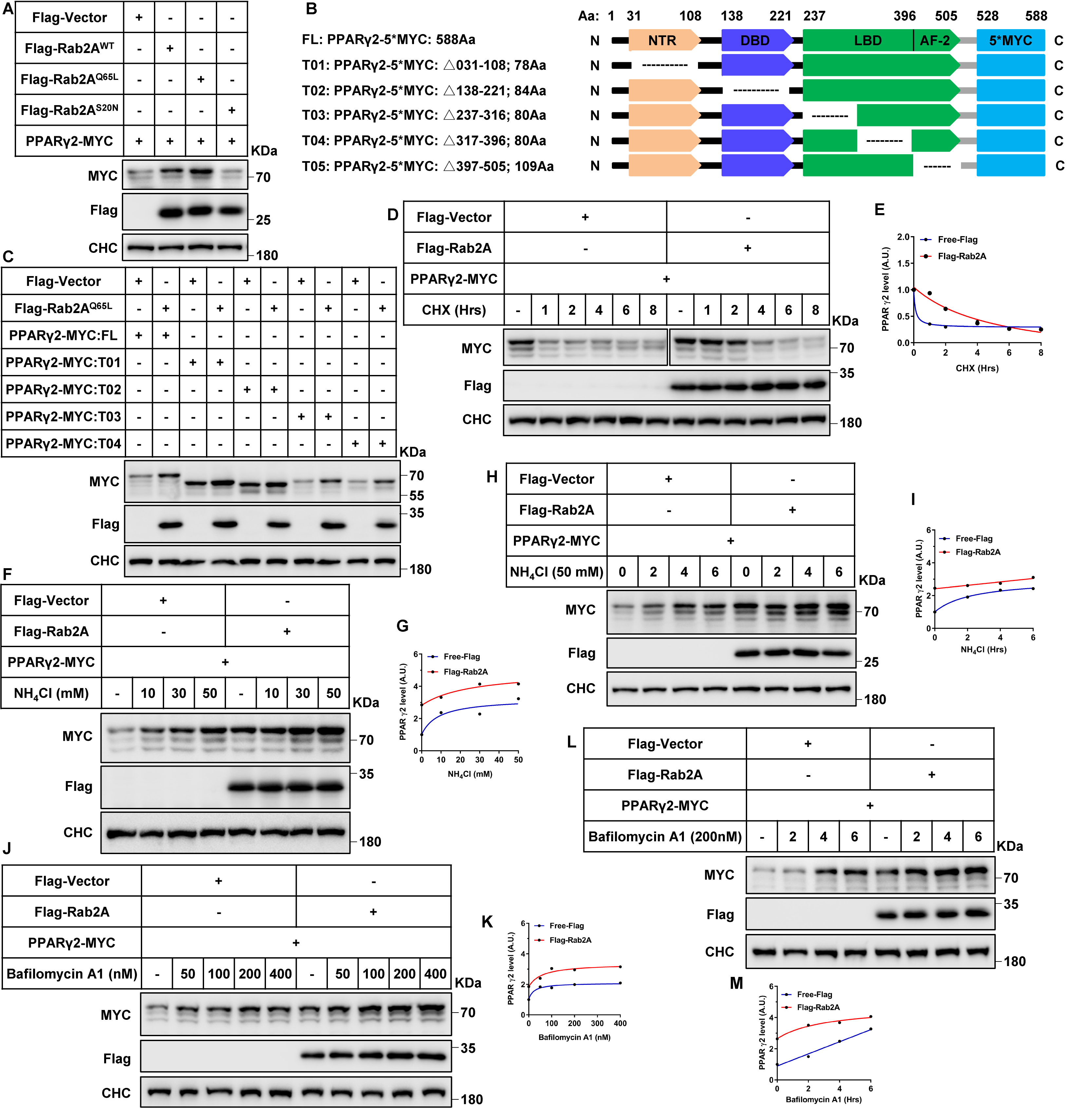

Supplement: S7 Fig — (A) The GTP-bound form of Rab2A increases the protein stability of exogenous PPARγ2. HEK293T cells were cultured and transfected with the indicated plasmids for 2 days, and the cells were then harvested and analyzed by immunoblotting. (B) Simplified models of different truncated plasmids of PPARγ2. (C) Mapping the detailed fragment of PPARγ2 regulated by Rab2A. HEK293T cells were cultured and transfected with the indicated plasmids for 2 days, and the cells were then harvested and analyzed by immunoblotting. (D, E) Overexpression of Rab2A attenuated the degradation of PPARγ2. HEK293T cells were cultured and transfected with the indicated plasmids, and 2 days later, the cells were stimulated with 200-μM CHX for the indicated hours. The cells were then harvested and analyzed by immunoblotting; the MYC blots were spliced to obtain a similar baseline protein level (D). (E) Quantification of PPARγ2 levels in D. The ratios in lanes 1 and 7 were defined as 1, respectively. (F–M) Rab2A does not regulate the lysosomal degradation of PPARγ2. HEK293T cells were cultured and transfected with the indicated plasmids for 2 days, and the cells were then stimulated with various concentrations of NH4Cl (F), bafilomycin A1 (J) or various times of NH4Cl (H), bafilomycin A1 (L). The cells were harvested and analyzed by immunoblotting. (G) Quantification of PPARγ2 levels in F. (I) Quantification of PPARγ2 levels in H. (K) Quantification of PPARγ2 levels in J. (M) Quantification of PPARγ2 level in L. The ratio in lane 1 was defined as 1. Raw data are given in S1 Excel spreadsheet with raw data from all figures. All the above experiments were performed at least twice with similar results. CHX, cycloheximide. (TIF) [file pbio.3001522.s007.tif]

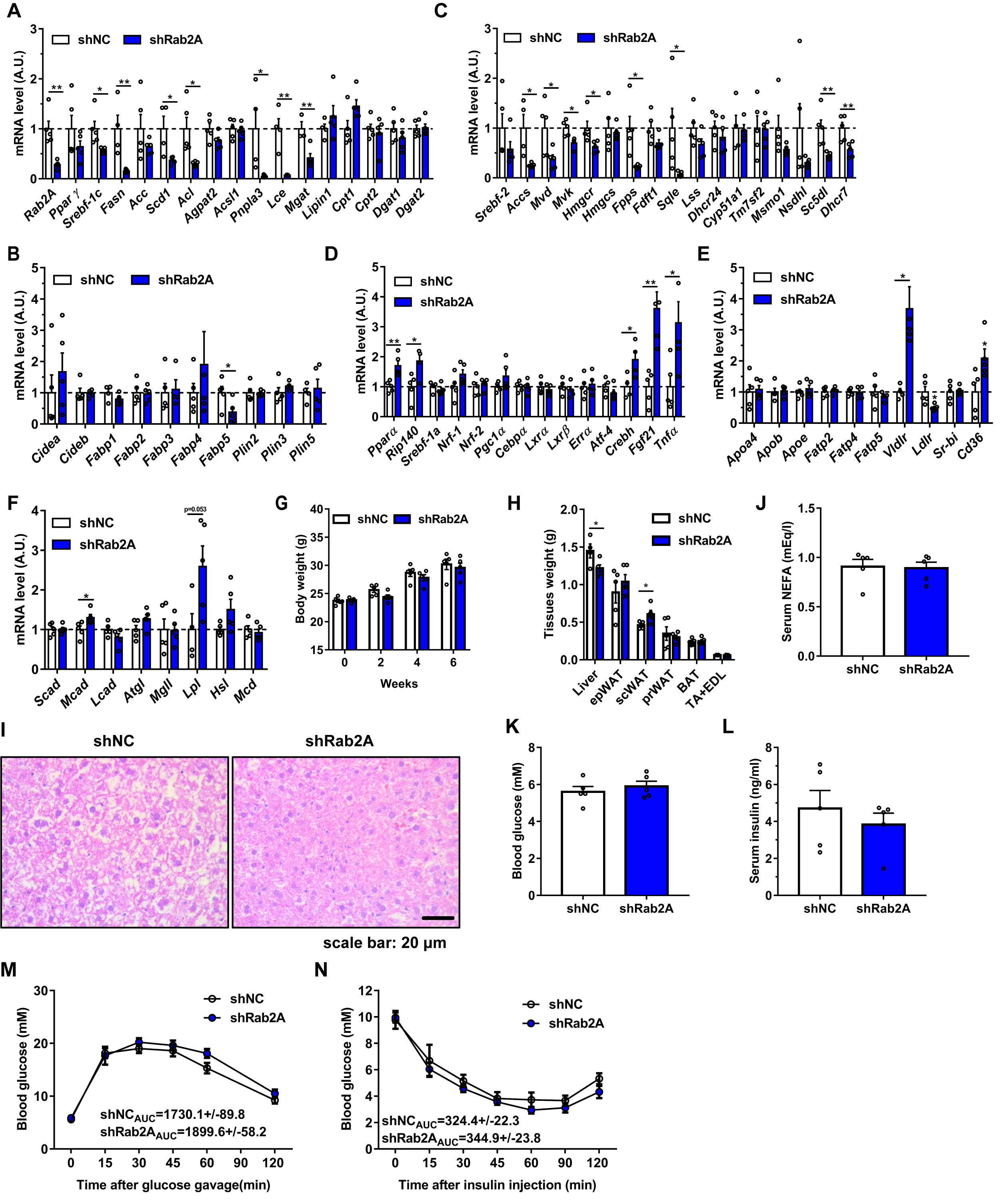

Supplement: S8 Fig — (A–F) Quantification of mRNA levels in Rab2A-knockdown and control liver samples. Male mice were intravenously injected with AAV8-Rab2A-shRNA virus and then fed a western diet for 2 months. The liver samples were analyzed by Q-PCR, and this analysis mostly focused on genes related to lipogenesis (A), lipid droplets (B), cholesterol synthesis (C), transcription factors (D), lipoprotein uptake and secretion (E), and lipolysis (F) (random feed, n = 5 per group). (G) Body weight of AAV8-Rab2A-shRNA and control mice (random feed, n = 5 per group). (H) Tissue weights of AAV8-Rab2A-shRNA and control mice (random feed, n = 5 per group). (I) Hematoxylin–eosin staining of liver sections from AAV8-Rab2A-shRNA and control mice (random feed, n = 5 per group). Representative images are shown. (J) NEFA levels in the serum of AAV8-Rab2A-shRNA and control mice (random feed, n = 5 per group). (K) Basal glucose level in the blood of AAV8-Rab2A-shRNA and control mice (overnight fast, n = 5 per group). (L) Insulin level in the serum of AAV8-Rab2A-shRNA and control mice (random feed, n = 5 per group). (M) OGTT of AAV8-Rab2A-shRNA and control mice (n = 5 per group). (N) ITT of AAV8-Rab2A-shRNA and control mice (n = 5 per group). The data were analyzed with unpaired 2-tailed Student t test and are presented as the means ± s.e.m.s. “*” indicates p < 0.05, “**” indicates p < 0.01, and “***” indicates p < 0.001. Raw data are given in S1 Excel spreadsheet with raw data from all figures. AAV8, adeno-associated virus serotype 8; EDL, extensor digitorum longus; epWAT, epididymis white adipose tissue; ITT, insulin tolerance test; NEFA, nonesterified fatty acid; OGTT, Oral glucose tolerance test; prWAT, perirenal white adipose tissue; scWAT, subcutaneous white adipose tissue; shRNA, short hairpin RNA; TA, tibialis anterior muscle. (TIF) [file pbio.3001522.s008.tif]
