# Supplementary material for: Rab2A regulates the progression of nonalcoholic fatty liver disease downstream of AMPK-TBC1D1 axis by stabilizing PPARγ
Source: PLoS Biol. 2022 Jan 21;20(1):e3001522. doi: 10.1371/journal.pbio.3001522 (PMC8809606; doi:10.1371/journal.pbio.3001522)
Supplement: S1 Supplemental Materials and Methods — (DOCX) [file pbio.3001522.s009.docx]

**Supplementary Materials and Methods**

**Reagents**

CHX (No. S7418), A769662 (No. S2697), bafilomycin (S1413), compound C (S7840) and MG132 (No. S2619) were purchased from Selleck Chemicals. We obtained MG-101 (ALLN) from MCE (No. HY-18964), NH_4_Cl from Sangon Biotech (No. A6000340500), anti-Flag affinity beads (No. SA042005) from Smart-Lifesciences (China), glutathione-Sepharose 4B beads from GE Healthcare (No. 17-0756-01), linear polyethylenimine (LPEI) from PolyScience (No. 24314-2), Lipofectamine-3000 transfection reagent from Thermo Fisher (P/N 100022052) and Insulin Aspart injection reagents from Novo Nordisk. All the other reagents were obtained from Sangon Biotech, Thermo Fisher or Sigma without specific statements.

**Cell culture**

Human embryonic kidney HEK293T cells and human liver carcinoma HepG2 cells were obtained from the Cell Resource Center, Chinese Academy of Medical Sciences and Peking Union Medical College (China). Mouse primary hepatocytes were isolated as previously described [1]. The cells were grown in a monolayer at 37°C with 5% CO2 and were maintained in DMEM (Biological Industries) containing 100 units/ml penicillin and 100 μg/ml streptomycin sulphate (Thermo Fisher) and supplemented with 10% fetal bovine serum (FBS, Biological Industries).

**TBC1D1^S231A^ knockin mouse model**

TBC1D1^Ser231Ala^-knockin mice were generated, housed and genotyped as previously described [1]. Briefly, the serine 231 site on TBC1D1 was mutated to alanine, and genotyping was performed with the following primers: 5’-TGGTTTACTGTGGCAGGAGGCAT-3’ and 5’-CACTGGGCTTTGTCTCT GATACTG-3’. All mice were housed in a pathogen-free environment with a 12-hour light/12-hour dark cycle and had free access to water consumption and food intake. All animal breeding, husbandry, care, euthanasia and use procedures followed the guidelines provided by the Ethics Committees of Nanjing University (Approval number MARC-CS3) and Anhui Medical University (Approval number LLSC20200327). .

**Mouse model of diet-induce obesity**

Male wild-type C57BL/6J mice were purchased from GemPharmatech (Nanjing, China), and the diet induced obese mice (DIO) were fed a western diet (No. D12079B, Research Diets) from 8 weeks to 20 weeks.

**AAV8-mediated Rab2A gene knockdown in the liver**

AAV8-mediated gene knockdown in the mouse liver was performed as previously described [2]. The AAV8 shRNA vectors pAAV-U6-shRNA/psgRNA v2.0-CMV-EGFP-WPRE and pAAV-U6-shRNA (Rab2A) v2.0-CMV-EGFP-WPRE were obtained from Obio Technology Co., Ltd. The sequence of shRNA-Rab2A (GCCTATCTCTTCAAGTACATCTTCAAGAGAGATGTACTTGAAGAGATAGGCT TTTTT) was previously described [3]. Male mice were randomized into groups for each experiment. Eight-week-old wild-type C57BL/6J mice were intravenously injected with 5 × 10^11^ vg AAV8 via the tail using a 29-gauge insulin syringe (BD). All tests were performed 6 weeks after AAV8 injection.

**Transfection, knockdown and plasmids**

Transient transfection was performed as previously described [4]. Briefly, HEK293T and HepG2 cells at densities of 6 × 10^5^ and 8 × 10^5^, respectively, were seeded in 60- mm dishes (two dishes for each sample) in DMEM supplemented with 10% FBS on day 0. On day 1, 2 μg of plasmids and 6 μg of linear polyethyleneimine were mixed and transfected into each dish. For the transient knockdown assay with siRNA, one day 1, the cells were transfected with a mixing solution containing 180 pM siRNA and 18 μl of Lipo3000. siRNA targeting human Rab2A was synthesized by Gene Pharma (Shanghai, China), and the sequence was as follows: 5’-CCAATTCCAACATGGTCAT-3’.

A stable expression cell line was constructed as previously described [5]. Briefly, the pHAGE-3*flag vector was used for the expression of cDNA, and the pLKO.1 vector was used for the expression of shRNA. The cells were transfected with the appropriate plasmids and screened with puromycin. The shRNA sequence of human Rab2A was as follows: 5’-CCGGTTCCATCACAAGGTCGTATTACCTCG AGGTAATACGACCTTGTGATGGATTTTTG-3’ and 5’-AATTCAAAAATCCATCACAAGGTCGTA TTA CCTCGAGGTAATACGACCTTGTGATGGA A-3’, The shRNA sequence of mouse Rab2A was as follows: 5’-CCGGGCCTATCTCTTCAAGTACATCCTCGAGGATGTACTTGAAGAGATAGGCTTT TTG-3’ and 5’-AATTCAAAAAGCCTATCTCTTCAAGTACATCCTCGAGGATGTACTTGAAGAG ATAGGC-3’, the shRNA sequence of human PPARγ was as follows: 5’-CCGGGTTTGAGTTTGCT GTGAAGCTCGAGCTTCACAGCAAACTCAAACTTTTTG-3’ and 5’-AATTCAAAAAGTTTGAGT TTGCTGTGAAGCTC GAGCTTCACAGCAAACTCAAAC-3’, the shRNA sequence of mouse PPARγ was as follows: 5’-CCGGGCTCCACACTATGAAGACATTCTCGAGAATGTCTTCATAGTGTGG AGCTTTTTG-3’ and 5’-AATTCAAAAAGCTCCACACTATGAAGACATTCTCGAGAATGTCTTC ATAGTGTGGAGC-3’.

The following plasmids were constructed by standard molecular cloning techniques: pHAGE-3*flag-TBC1D1 encodes full-length human TBC1D1 with a FLAG epitope tag, pHAGE-3*flag-Rab2A and pCDNA3-3*flag-Rab2A encodes full-length mouse Rab2A with a FLAG epitope tag, pCDNA3-EGFP-Rab2A encodes full-length mouse Rab2A with a EGFP epitope tag, pCDNA3-3*flag-Rab2B encodes full-length mouse Rab2B with a FLAG epitope tag, pCDNA3-3*flag-Rab8A encodes full-length mouse Rab8A with a FLAG epitope tag, pCDNA3-3*flag-Rab8B encodes full-length mouse Rab8B with a FALG epitope tag, pCDNA3-3*flag-Rab10 encodes full-length mouse Rab10 with a FLAG epitope tag, pCDNA3-3*flag-Rab14 encodes full-length mouse Rab14 with a FLAG epitope tag, pCDNA3-3*flag-Rab1A encodes full-length human Rab1A with a FLAG epitope tag, pCDNA3-3*flag-Rab24 encodes full-length human Rab24 with a FLAG epitope tag, pCDNA3-3*flag-Rab35 encodes full-length human Rab35 with a FLAG epitope tag, pCDNA3-3*flag-Rab7A encodes full-length human Rab7A with a FLAG epitope tag, pCDNA3-3*flag-Rab15 encodes full-length human Rab15 with a FLAG epitope tag, pCDNA3-3*flag-Rab40A encodes full-length human Rab40A with a FLAG epitope tag, pCDNA3-3*flag-Rab1B encodes full-length human Rab1B with a FLAG epitope tag, pCDNA3-3*flag-Rab5A encodes full-length human Rab5A with a FLAG epitope tag, pCDNA3-3*flag-Rab9A encodes full-length human Rab9A with a FLAG epitope tag, pCDNA3-3*flag-Rab9B encodes full-length human Rab9B with a FLAG epitope tag, pCDNA3-3*flag-Rab11B encodes full-length human Rab11B with a FLAG epitope tag, pCDNA3-3*flag-Rab22B encodes full-length human Rab22B with a FLAG epitope tag, pCDNA3-3*flag-Rab32 encodes full-length human Rab32 with a FLAG epitope tag, pCDNA3-3*flag-Rab4B encodes full-length human Rab4B with a FLAG epitope tag, pCDNA3-3*flag-Rab39A encodes full-length human Rab39A with a FLAG epitope tag, pCDNA3-3*flag-Rab13 encodes full-length human Rab13 with a FLAG epitope tag, pCDNA3-3*flag-Rab23 encodes full-length human Rab23 with a FLAG epitope tag,pCDNA3-PPARγ1-5*MYC encodes full-length mouse PPARγ1 followed by a MYC epitope tag, pCDNA3-PPARγ2-5*MYC encodes full-length mouse PPARγ2 followed by a MYC epitope tag, pCDNA3-PPARγ2-mCherry encodes full-length mouse PPARγ2 followed by a mCherry epitope tag, pGEX-6p-1-GST- PPARγ2 encodes full-length human PPARγ2 with a GST epitope tag and pGEX-6p-1-GST-GRASP55 encodes full-length human GRASP55 with a GST epitope tag. pCDNA3-HA-TBC1D1, pCDNA3-HA-TBC1D1^S237A^, pCDNA3-HA-TBC1D1^S237A/R854K^ and pCDNA3-HA-TBC1D1^R854K^ were constructed as previously described (1). pHAGE-3*flag-TBC1D1^S237A^, pHAGE-3*flag-Rab2A^Q65L^ and pHAGE-3*flag-Rab2A^S20N^ were mutated by quick-change mutagenesis. Truncated plasmids of pCDNA3-PPARγ2-5*MYC were also constructed using standard molecular cloning techniques.

**Glucose starvation**

Glucose starvation experiments were performed as previously described [6]. In brief, the culture and transfection of the cells were performed as described above. At day 2, cells were rinsed twice with PBS. Then, for the time curve assay, cells were incubated in glucose-free DMEM (Gibco, cat. 11966) supplemented with 10% FBS and 1 mM sodium pyruvate (Gibco, cat. 11360) for desired periods of time. For the concentration curve assay, cells were incubated with glucose-free DMEM supplemented with 10% FBS, 1 mM sodium pyruvate and different concentrations of glucose. The samples were collected for western blotting.

**Immunoblotting and antibodies**

Immunoblotting of cell or tissue samples was performed as previously described [7]. Briefly, the samples were collected and homogenized in RIPA buffer (50 mM Tris-HCl, pH 8.0, 150 mM NaCl, 1.0% Triton X-100, 0.1% SDS, and 0.5% sodium deoxycholate) supplemented with 1.0 mM Na3VO4, 1.0 mM NaF, 1.0 mM benzamidine, 0.4 mM phenylmethylsulfonyl fluoride, 5.0 mM EDTA, 1.0 mM EGTA, 0.1 mg/ml leupeptin, 0.1 mg/ml aprotinin, 0.1 mg/ml pepstatin, 0.25 mM dithiothreitol and protease inhibitor cocktail followed 10 times through a 22G needle. The protein concentrations were detected using Bradford reagent as previously described [8]. Equal amounts of the samples were mixed with 4×loading buffer (150 mM Tris-HCl, pH 6.8, 12% SDS, 30% glycerol, 6% 2-mercaptoethanol and 0.05% bromophenol blue), heated at 100°C for 10 minutes, resolved by SDS-PAGE and transferred to PVDF or NC (nitrocellulose) membranes. The membranes were then blocked with 5% fat-free milk in Tris-buffered saline buffer (TBS) containing 0.1% TWEEN-20 and incubated with the indicated primary antibody overnight at 4°C. After washing in TBST buffer for approximately 40 minutes, the membranes were incubated in 5% fat-free milk supplemented with the corresponding peroxidase-AffiniPure secondary antibodies (1:10,000) at room temperature for 2 hours. The blots were then washed for 40 minutes, and western blotting signals were acquired with an autoradiography machine (Tanon-5200). Quantification of the protein levels was performed with ImageJ (National Institutes of Health, https://imagej.nih.gov/ij/) and normalized to the level of clathrin heavy chain (CHC) or GAPDH. Detailed information on antibodies is provided in **Table S1**.

**Immunoprecipitation**

The cell samples (at least 2 mg of proteins) were collected and homogenized in 600 μl of IP buffer (50 mM Tris-HCl, pH 7.6, 150 mM NaCl and 0.5% NP-40) containing various protease inhibitors. After needling and centrifugation, 75 μl of the lysates was mixed with 25 μl of 4× loading buffer to obtain the input samples. The resulting samples (500 μl) were incubated with 20 μl of beads at 4°C for approximately 4 hours. The beads were then collected, washed six to eight times with IP buffer and boiled with 1× loading buffer to obtain the pellet samples. The aliquots were analysed by immunoblotting.

For the in-vitro binding assay, recombinant proteins including Flag-TBC1D1, Flag-TBC1D1^S237A^ or Flag-Rab2A were collected from HepG2 cell lysates according to the above methods and then eluted with Flag peptides in a metal bath at 37℃ for 30 minutes. Then GST-PPARγ proteins prebound to the beads were added to form a complex with Flag-TBC1D1, Flag-TBC1D1^S237A^ or Flag-Rab2A at 4 ℃ for 2 hours. Beads were centrifuged and washed with IP buffer for 6-8 times before adding 1× loading buffer. The proteins’ binding was analysed by immunoblotting.

**RNA isolation and quantitative PCR**

Cell or mouse liver samples were collected and homogenized in TRIzol Reagent (#9109, RNAiso Plus, Takara). Total RNA was separated with trichloromethane, isopropanol and ethanol following the manufacturer’s instructions. One microgram of RNA was reverse-transcribed into cDNA using HiScript III RT SuperMix (+gDNA wiper) (#R323-01, Vazyme Biotechnology) after quality control. Quantitative PCR (Q-PCR) was performed using AceQ qPCR SYBR Green Master Mix (#Q111-02/03, Vazyme Biotechnology) and analysed with an Applied Biosystems StepOnePlus Real-Time PCR system. The primer sequences are listed in **Table S2**.

**RNA sequencing**

The RNA quality was determined with a 2100 Bioanalyzer (Agilent) and quantified using an ND-2000 instrument (NanoDrop Technologies). Only high-quality RNA samples (OD260/280=1.8~2.2, OD260/230≥2.0, RIN≥6.5, 28S:18S≥1.0, >2 μg) were used to construct a sequencing library by Shanghai Majorbio Bio-pharm Technology Co., Ltd., and the data were analysed using the free online platform of the Majorbio Cloud Platform (www.majorbio.com) [9]. Briefly, the RNA-seq transcriptome library was prepared with the TruSeq RNA sample preparation kit from Illumina (San Diego, CA, USA) using 1 μg of total RNA according to Illumina’s library construction protocol. After quantification by TBS380, the paired-end RNA-seq sequencing library was sequenced with the Illumina HiSeq xten/NovaSeq 6000 sequencers (2×150 bp read length).

The raw paired end reads were trimmed and subjected to quality control by SeqPrep (https://github.com/jstjohn/SeqPrep) and Sickle (https://github.com/najoshi/sickle) with the default parameters. For the identification of differentially expressed genes (DEGs) between two different samples, the expression level of each transcript was calculated according to the transcripts per million reads (TPM) method. RSEM (http://deweylab.biostat.wisc.edu/rsem/) was used to quantify the gene abundances. EdgeR software in the R statistical package (Empirical analysis of Digital Gene Expression in R; http://www.bioconductor.org/packages/2.12/bioc/html/edgeR.html) was utilized for differential expression analysis. In addition, functional enrichment analysis including GO and KEGG analyses was performed to identify which GO terms and metabolic pathways were significantly enriched with the DEGs based on a P value ≤0.05 and |log2FC| >=0.000 compared with the whole-transcriptome background. GO functional enrichment and KEGG pathway analysis were performed using Goatools (https://github.com/tanghaibao/Goatools) and KOBAS (http://kobas.cbi.pku.edu.cn/home.do).

Lipid metabolism-related genes of the significant genes were then screened via comparisons with six databases including NR (ftp://ftp.ncbi.nlm.nih.gov/blast/db/), Pfam (http://pfam.xfam.org/), Swiss-Prot (http://web.expasy.org/docs/swiss-prot_guideline.html), EggNOG (Clusters of Orthologous Groups of proteins, http://www.ncbi.nlm.nih.gov/COG/), GO (Gene Ontology, http://www.geneontology.org) and KEGG (Kyoto Encyclopedia of Genes and Genomes, http://www.genome.jp/kegg/). Heatmaps were then constructed using the pheatmap package in R based on the selected genes according to the following criteria: Benjamini/Hochberg-adjusted p value ≤0.05 and |log2FC| >=1.

**Prokaryotic expression and purification**

Prokaryotic expression and purification of GST-GRASP55 and GST-PPARγ2 recombinant protein were performed according to the handbook of GE Healthcare Life Sciences. Briefly, Escherichia coli BL21(DE3) was transformed with pGEX-6p-1-GST-GRASP55 plasmids or pGEX-6p-1-GST-PPARγ2 plasmids, and after expression was induced with IPTG (isopropyl β-D-1-thiogalactopyranoside) at 22°C for 4 hours, the cells were homogenized in lysis buffer (50 mM Tris-HCl, pH 8.0, 150 mM NaCl, 0.5 mM EDTA, 0.5 mM EGTA, 500 μM PMSF, 10 U/ml Dnase I, 5 mM DTT, 1% Triton X-100 and protease inhibitor cocktail). Purified GST recombinant protein was captured with glutathione Sepharose 4B beads (GE Healthcare) after overnight incubation at 4°C.

**Pull down assay of Rab2A-GTP**

The collection and homogenization of cell or liver samples were performed using the same protocol as that used for the immunoblotting. A previous study showed that GRASP55 binds specifically to the GTP-bound form of Rab2A [10]. For the Rab2A-GTP pulldown assay, protein lysates (approximately 2 mg) were incubated with purified GST-GRASP55 recombinant protein overnight, and the GST-GRASP55/Rab2A-GTP complex was captured with glutathione Sepharose 4B beads. After washing and removing the nonspecifically bound proteins, the sample was subjected to western blotting, and the protein level of GTP-Rab2A was detected with Rab2A antibody.

**Nuclear and Cytoplasmic extraction**

The culture and transfection of cell samples were performed as described above. Collection, homogenization and extraction were performed according to the standard protocol of Thermo Science (78833). After protein extraction, the sample was subjected to western blotting. p53 was chosen as the nuclear protein marker and GAPDH was the cytoplasmic protein marker.

**Immunofluorescence staining and imaging**

Immunofluorescence staining of HEK293T cells was performed according to the standard protocol. Briefly, the 293T cells were washed twice with PBS buffer and fixed in 4% paraformaldehyde for 20 minutes. The cells were then permeabilized with 0.1% Triton X-100 in PBS for another 20 minutes.

For cellular lipid droplet staining, the cells were incubated with BODIPY (1 μg/ml, (4,4-Difluoro-1,3,5,7,8-Pentamethyl-4-Bora-3a,4a-Diaza-s-Indacene), D3922, Thermo Fisher) for 30 minutes before fixation. Finally, the slides were mounted, and photographs were taken with a Zeiss confocal microscope (LSM800). Representative results are shown. At least 300 cells were selected to quantify the immunofluorescence intensity of cellular lipid droplets.

**Oil Red O staining and imaging**

Oil Red O was dissolved in isopropanol at a stock concentration of 0.5% (w/v), and the working solution was blended with 60% stock solution and 40% distilled water. The cells were stained with Oil Red O reagents as previously described [11]. Briefly, the cells were first fixed with 4% paraformaldehyde in PBS at room temperature for 30 minutes, washed twice with PBS and incubated with 60% isopropanol for 5 minutes. After removing the isopropanol, the cells were stained in Oil Red O working solution for 30 minutes and washed thoroughly with PBS, and images were taken with a phase-contrast microscope (Leica DMi1). The intensity of the Oil Red O-stained lipid droplets was analysed and quantified with ImageJ software (National Institutes of Health) from at least 15 different visual fields, and representative results are shown.

**Histology and imaging**

Histology and imaging were performed as previously described [1]. Briefly, fresh liver samples were first fixed with 4% paraformaldehyde overnight, dehydrated with tandem alcohol, embedded in paraffin wax and sectioned using a Leica RM2255 microtome to a thickness of 6-10 μm. The dry liver slices were stained with hematoxylin-eosin according to the standard protocol. Images were obtained with a phase-contrast microscope (Leica DMi1). The sizes of the lipid droplets were analysed with ImageJ software (National Institutes of Health) from at least 200 droplets per sample.

**Blood chemistry**

Mouse blood plasma was collected by retro-orbital bleeding. The plasma triglyceride, NEFA and total cholesterol levels were determined using a Wako LabAssay Triglyceride kit (290-63701), Wako LabAssay NEFA kit (294-63601) and LabAssay Cholesterol kit (294-65801) (Wako Chemicals USA, Inc.). The plasma insulin levels were detected by a rat/mouse insulin ELISA kit (EMD Millipore Corporation, #EZRMI-13K).

**Measurement of liver and cellular TC and TG levels**

The TC (total cholesterol) and TG (triglyceride) contents in the liver and cell lines were detected as previously described [12]. Briefly, the frozen liver blocks were weighed, and the cells were quantified to ensure a quantity higher than 1×10^7^ cells. For the measurement of the TG level, the samples were saponified in ethanolic KOH, glycerol was extracted with ethanol and MgCl2, and the levels were determined with the free glycerol reagent (F6428, Sigma-Aldrich) using glycerol (G7793, Sigma-Aldrich) as the standard for the calculation or triglyceride assay kit (A110-1-1, Nanjing Jiancheng Bioengineering Institute, China). For the measurement of TC levels, cholesterol was extracted with a chloroform-isopropanol-NP-40 (7:11:0.1) solution, and the level was measured using a LabAssay Cholesterol kit (catalogue no. 294–65801; Wako Chemicals USA, Inc.) with the standard protocol.

**Oral glucose tolerance test and insulin tolerance test**

The oral glucose tolerance test (OGTT) and insulin tolerance test (ITT) were performed as previously described (1). Briefly, the mice were deprived of food for 16 hours (OGTT) or 4 hours (ITT) before measurement of the initial blood glucose levels using Accu-Chek Performa test strips (Roche). For the OGTT, the mice were orally administered glucose solution (1.5 mg/g of body weight) via gavage. For the ITT, the mice received an intraperitoneal injection of insulin (0.75 mU/g body weight for DIO mice and 1.5 mU/g body weight for OB/OB mice). In both tests, the tail blood glucose levels were measured at 0, 15, 30, 60, and 120 minutes.

**Statistical analysis**

All statistical data are presented as the mean ± s.e.m.s. All experiments were repeated at least twice independently with similar results. All statistical analyses were performed using GraphPad Prism 7 (GraphPad Software). Unless stated, all the data were analyzed by unpaired two-tailed Student’s t-test, and differences were considered statistically significant at P<0.05.

Table S1: Antibodies

| **Primary antibodies** | **Dilution** | **Isotype** | **Source** | **Identifier** |
| --- | --- | --- | --- | --- |
| FLAG | 1:1,000 | Mouse | Sigma | Cat# F1804 |
| MYC | 1:1,000 | Mouse | ATCC | Cat# CRL-1729 |
| GAPDH | 1:1,000 | Rabbit | Proteintech Technology | Cat# 10494-1-AP |
| HA | 1:1,000 | Mouse | Biolegend | Cat# MMS-101P |
| CHC | 1:1,000 | Mouse | BD Transduction Laboratories | Cat# 610500 |
| p^Ser231^TBC1D1 | 1:1,000 | Rabbit | Millipore | Cat# 07-2268 |
| TBC1D1 | 1:1,000 | Rabbit | Cell Signaling Technology | Cat# 4629 |
| PPARγ | 1:1,000 | Rabbit | Cell Signaling Technology | Cat# 2435S |
| FASN | 1:1,000 | Rabbit | Cell Signaling Technology | Cat# 3189/3180 |
| ACS1 | 1:1,000 | Rabbit | Cell Signaling Technology | Cat# 3658 |
| ACL | 1:1,000 | Rabbit | Cell Signaling Technology | Cat# 13390 |
| ACSL1 | 1:1,000 | Rabbit | Cell Signaling Technology | Cat# 9189 |
| CD36 | 1:1,000 | Rabbit | Cell Signaling Technology | Cat# 14347 |
| ATGL | 1:1,000 | Rabbit | Cell Signaling Technology | Cat# 2439 |
| p^Ser473^-PKB | 1:1,000 | Rabbit | Cell Signaling Technology | Cat# 9271 |
| PKB | 1:1,000 | Rabbit | Cell Signaling Technology | Cat# 9272 |
| CREB | 1:1,000 | Rabbit | Cell Signaling Technology | Cat# 9197 |
| DHCR24 | 1:1,000 | Rabbit | Cell Signaling Technology | Cat# 2033 |
| p^Ser133^CREB | 1:1,000 | Rabbit | Cell Signaling Technology | Cat# 9189 |
| p53 | 1:1,000 | Rabbit | Cell Signaling Technology | Cat# 2527 |
| FSP27 | 1:1,000 | Rabbit | Peng Li, Tsinghua university | [13] |
| RAB2A | 1:2,000 | Mouse | Proteintech Technology | Cat# 67501-1-Ig |
| CIDEA | 1:1,000 | Rabbit | Proteintech Technology | Cat# 13170-1-AP |
| PLIN2 | 1:1,000 | Rabbit | Proteintech Technology | Cat# 15294-1-AP |
| PLIN3 | 1:1,000 | Rabbit | Proteintech Technology | Cat# 10694-1-AP |
| LPL (Lipoprotein lipase) | 1:1,000 | Rabbit | Proteintech Technology | Cat# 16899-1-AP |
| PPARγ(E-8) | 1:1,000 | Mouse | Santa Cruz Technology | Cat# sc-7273 |
| SREBP-1(2A4) | 1:1,000 | Mouse | Santa Cruz Technology | Cat# sc-13551 |
| GST (1E5) | 1:1,000 | Mouse | Santa Cruz Technology | Cat# sc-53909 |
| FGF21 | 1:1,000 | Goat | R&D Systems | Cat# AF3057 |
| **Secondary antibodies** | **Dilution** | **Source** | | **Identifier** |
| Peroxidase-AffiniPure Goat Anti-Mouse IgG (H+L) | 1:1,0000 | Jackson ImmunoResearch Laboratories | | Cat# 115-035-003 |
| Peroxidase-AffiniPure Goat Anti-Rabbit IgG (H+L) | 1:1,0000 | Jackson ImmunoResearch Laboratories | | Cat# 111-035-003 |
| Peroxidase-AffiniPure Rabbit Anti-Goat IgG (H+L) | 1:1,0000 | Jackson ImmunoResearch Laboratories | | Cat# 305-035-003 |

Table S2: Primer sequences for Q-PCR

| **Species** | **Gene Name** | **Forward primer** | **Reverse Primer** |
| --- | --- | --- | --- |
| Human | *36B4* | TGCATCAGTACCCCATTCTATCA | AAGGTGTAATCCGTCTCCACAGA |
| Human | *GAPDH* | GCCCCAGCGTCAAAGGT | GGCATCCTGGGCTACACTGA |
| Human | *PPARγ1* | AAAGAAGCCAACACTAAACC | CTTCCATTACGGAGAGATCC |
| Human | *PPARγ2* | GCGATTCCTTCACTGATAC | CTTCCATTACGGAGAGATCC |
| Human | *PPARγ1+2* | GGCTTCATGACAAGGGAGTTTC | AAACTCAAACTTGGGCTCCATAAA |
| Human | *Rab2A* | CGTTCCATCACAAGGTCGTATT | CTTAGCAGACGTTTCCATGAAGA |
| Human | *CIDEA* | AGACCTTGGGAGACAACACG | ACAGGAACCGCAGCAGAC |
| Human | *CIDEB* | CAGCGACCTTTCCGTGTCT | GGGTCTCCAATGCTTTGGCT |
| Human | *FSP27* | AAGTCCCTTAGCCTTCTCTACC | CCTTCCTCACGCTTCGATCC |
| Human | *PLIN1* | TGTGCAATGCCTATGAGAAGG | AGGGCGGGGATCTTTTCCT |
| Human | *PLIN2* | TTGCAGTTGCCAATACCTATGC | CCAGTCACAGTAGTCGTCACA |
| Human | *PLIN3* | TATGCCTCCACCAAGGAGAG | ATTCGCTGGCTGATGCAATCT |
| Human | *PLIN4* | CTGGTGGCCAACGCACATAG | GCCCCGGACACCATCTTTTC |
| Human | *PLIN5* | AAGGCCCTGAAGTGGGTTC | GCATGTGGTCTATCAGCTCCA |
| Human | *FABP1* | CGGAAATCGTGCAGAATGG | AATTCGTTTTGGATCACTTTGGA |
| Human | *FABP2* | ATGGCGTTTGACAGCACTTG | TCAGTTCCGTCTGCTAGATTGTA |
| Human | *FABP3* | CATGACCAAGCCTACCACAAT | CCCCAACTTAAAGCTGATCTCTG |
| Human | *FABP4* | TGGGATGGAAAATCAACC | TCTCTCATAAACTCTCGTGG |
| Human | *FABP5* | AAAACTGAGAGCACTTTGAAAACAAC | TTTCTGCCATCAGCTGTGGTT |
| Human | *CD36* | TCTTTCCTGCAGCCCAATG | TGTCAGCCTCTGTTCCAACTG |
| Mouse | *36B4* | CACTGGTCTAGGACCCGAGAAG | GGTGCCTCTGGAGATTTTCG |
| Mouse | *Gapdh* | TGTGTCCGTCGTGGATCTGA | CCTGCTTCACCACCTTCTTGAT |
| Mouse | *Plin1* | GGAGAACGTGCTCAGAGAGG | GATGGTGTTCCGGAGAGTGT |
| Mouse | *Plin2* | GGCGTCTCTTTTCTCCAGGA | CGGATCCACTACTGCTGCTG |
| Mouse | *Plin3* | GGTTTTGGCGGATACTAA | AGCTAGATACCATTTCTTGAG |
| Mouse | *Plin4* | CCCCTCATCTAAAGTGTC | AGCTGTCTGTTCAGAAG |
| Mouse | *Plin5* | GCAACAGGGCTACTTTG | GTTCATAGGCGAGATGG |
| Mouse | *Cidea* | GCCGTGTTAAGGAATCTGCTG | TGCTCTTCTGTATCGCCCAGT |
| Mouse | *Cideb* | CCCAAAGCAACAGGGAGAGAGT | AGTTCCAGACCCTACGGCTT |
| Mouse | *Cidec* | ATGGACTACGCCATGAAGTCT | CGGTGCTAACACGACAGGG |
| Mouse | *Lipin1* | GAGCATGCCAAGACCAACATC | CAATGGGAAGACGTGATCGA |
| Mouse | *Cish* | TCTTGTCCTTTGCTGGCTGT | TAGGAATGTACCCTCCGGCA |
| Mouse | *Enho* | ATCTCCCAAGGGGCTCTCAT | GTCAAATCGACTGGACCCCA |
| Mouse | *Socs3* | CACCTGGACTCCTATGAGAAAGTG | GAGCATCATACTGATCCAGGAACT |
| Mouse | *Tnf* | CTGAGGTCAATCTGCCCAAGTAC | CTTCACAGAGCAATGACTCCAAAG |
| Mouse | *Alox5ap* | TCTTTGCCCACAAGGTGGAG | GGCGAAGGACATGAGGAACA |
| Mouse | *Cyp7b1* | TAGCCCTCTTTCCTCCACTCATA | GAACCGATCGAACCTAAATTCCT |
| Mouse | *Cyp2g1* | CACGGGACACTCATTTCCGA | AGGCTTTTCCCCCAGATTGT |
| Mouse | *Fsp27α* | GCCACGCGGTATTGCCAGGA | GGGTCTCCCGGCTGGGCTTA |
| Mouse | *Fsp27β* | GTGACCACAGCTTGGGTCGGA | GGGTCTCCCGGCTGGGCTTA |
| Mouse | *Pparγ1* | TGTGTGACAGACAAGATTTGAAAGAA | GCATCTCTGTGTCAACCATGGT |
| Mouse | *Pparγ2* | TCGCTGATGCACTGCCTATG | GAGAGGTCCACAGAGCTGATT |
| Mouse | *Pparγ1+2* | CACAATGCCATCAGGTTTGG | GCTGGTCGATATCACTGGAGATC |
| Mouse | *Lpl* | ACTCTGTGTCTAACTGCCACTTCAA | ATACATTCCCGTTACCGTCCAT |
| Mouse | *Atgl* | CAACGCCACTCACATCTACG | ACCAGGTTGAAGGAGGGATG |
| Mouse | *Hsl* | GGAGCACTACAAACGCAACGA | TCGGCCACCGGTAAAGAG |
| Mouse | *Mgll* | GACGGACAGTACCTCTTTTG | AGAAAAGTAGGTTGGCCTCT |
| Mouse | *Mcd* | GGAGACAGGCCCCAACAGT | TGAGGATCTGCTCGGAAGCT |
| Mouse | *Lcad* | TCAATGGAAGCAAGGTGTTCA | GCCACGACGATCACGAGAT |
| Mouse | *Scad* | CGCCTCCACGGGAGTTATC | TGCGGATCCAAACTTCAGAAT |
| Mouse | *Pnpla3* | CGAGGCGAGCGGTACGT | TGACACCGTGATGGTGGTTT |
| Mouse | *Cgi-58* | TCCCCTTTCCTTCCAGTATTCC | CCCGATAGGCAACAGTGTTAACA |
| Mouse | *Cd36* | GAATGGGCTGTGATCGGAAC | ACGTCATCTGGGTTTTGCAC |
| Mouse | *Ldlr* | AGGCTGTGGGCTCCATAGG | TGCGGTCCAGGGTCATCT |
| Mouse | *Vldlr* | ACCTGTTCCTGTCCCAATGG | TCACTGTAAGTCACAGGAGTTGAAGTAC |
| Mouse | *Sr-bi* | TCCCCATGAACTGTTCTGTGAA | TGCCCGATGCCCTTGA |
| Mouse | *Apoa4* | ACCCAGCTAAGCAACAATGC | GCATCCCCAAGTTTGTCCTT |
| Mouse | *Apob* | CGTGGGCTCCAGCATTCTA | TCACCAGTCATTTCTGCCTTTG |
| Mouse | *Apoe* | GCAGGCGGAGATCTTCCA | CCACTGGCGATGCATGTC |
| Mouse | *Fatp1* | CTACCACTCTGCAGGGAACA | CAGGTAGCGGCAGATTTCAC |
| Mouse | *Fatp2* | CAACACACCGCAGAAACCA | ATTTCCCAGGGCTTTTTTCA |
| Mouse | *Fatp3* | CAGCTCTACAGCCATGTTTCTGA | CAAAGATTCCTGGAGCCTGAGA |
| Mouse | *Fatp4* | TTGCAAGTCCCATCAGCAAC | AACAGCGGGTCTTTCACAAC |
| Mouse | *Fatp5* | ACGCTTTGGTCCCATTCG | CATAGTTCATTAAGCCCACATTGC |
| Mouse | *Acc1* | TGACAGACTGATCGCAGAGAAAG | TGGAGAGCCCCACACACA |
| Mouse | *Acsl1* | CGCACCCTTCCAACCAACA | CGCTATTTCCACTGACTGCAT |
| Mouse | *Pparα* | CCACGAAGCCTACCTGAAGA | TTCTCGGCCATACACAAGGT |
| Mouse | *Pgc1α* | ATACCGCAAAGAGCACGAGAAG | AGCAGCGAAAGCGTCACAG |
| Mouse | *Cebpα* | CAAGAACAGCAACGAGTACCG | GTCACTGGTCAACTCCAGCAC |
| Mouse | *Crebh* | GGGTCAACAGGCACCATGAG | TCTAGCGTCACCGGATGTTG |
| Mouse | *Srebf-1c* | GGAGCCATGGATTGCACATT | GGCCCGGGAAGTCACTGT |
| Mouse | *Srebf-2* | GCGTTCTGGAGACCATGGA | ACAAAGTTGCTCTGAAAACAAATCA |
| Mouse | *Nrf-1* | CCACGGTAGCGCAGC | ACATGGACCTGCTGGACTTG |
| Mouse | *Rip140* | CGAACTCGGGAGGCGAC | CTTCAGATTCTCCGGGCCTC |
| Mouse | *Errα* | GTGTGAGATCACCAAGCGGA | GGCGTACAGCTTCTCAGGTT |
| Mouse | *Acl* | ACTTTCTCATTGAACCCTTCGTCC | ATCCACATCGCCCACATCCAC |
| Mouse | *Scd1* | TCCCTCCGGAAATGAACGAGAGAA | AGTGCAGCAGGACCATGAGAATGA |
| Mouse | *Dgat1* | GAGGCCTCTCTGCCCCTATG | GCCCCTGGACAACACAGACT |
| Mouse | *Dgat2* | CCGCAAAGGCTTTGTGAAG | GGAATAAGTGGGAACCAGATCA |
| Mouse | *Fasn* | GCTGCGGAAACTTCAGGAAAT | AGAGACGTGTCACTCCTGGACTT |
| Mouse | *Lce* | AGAGAACACGTAGCGACTCCG | ACCACCAAAGATAAAGGCAGCG |
| Mouse | *Gpat1* | CAACACCATCCCCGACATC | GTGACCTTCGATTATGCGATCA |
| Mouse | *Agpat2* | TTTGAGGTCAGCGGACAGAA | AGGATGCTCTGGTGATTAGAGATGA |
| Mouse | *Cpt1* | CACCAACGGGCTCATCTTCTA | CAAAATGACCTAGCCTTCTATCGAA |
| Mouse | *Cpt2* | AGCCTACCTGGTCAATGCATATC | GGGTTTGGGTATACGAGTTGAATT |
| Mouse | *Lipin2* | GCATTAACCAAGCCACGTTGT | AAAGGCGAGCACTGGTAGGA |
| Mouse | *Fabp1* | GGTGACAACTTTCAAAGGCATAAA | TGTCGCCCAATGTCATGGTA |
| Mouse | *Fabp2* | GTTGAAGGCAGAGTAGGAATGA | GGAAAGCAGAGGTCCAGTCC |
| Mouse | *Fabp3* | CCC CTC AGC TCA GCA CCA T | CAG AAAAATCCCAACCCAAGAAT |
| Mouse | *Fabp4* | AGCTGGTGGTGGAATGTGTT | AATTTCCATCCAGGCCTCTT |
| Mouse | *Fabp5* | CGGGTCTATGAGAAGGTGCAA | GAGCATATTCACTCTGGCAGCTAA |
| Mouse | *Hmgcs* | GCCGTGAACTGGGTCGAA | GCATATATAGCAATGTCTCCTGCAA |
| Mouse | *Hmgcr* | CTTGTGGAATGCCTTGTGATTG | AGCCGAAGCAGCACATGAT |
| Mouse | *Mvk* | GGACACGAGCTTTCTTGAGC | GCAGATTGCCAGGTACAGGT |
| Mouse | *Fpps* | ATGGAGATGGGCGAGTTCTTC | CCGACCTTTCCCGTCACA |
| Mouse | *Fdft1* | TCCCTGACGTCCTCACCTAC | GGGGATCCGGTGATAAATCT |
| Mouse | *Lss* | GCTGCATGTGGTGTATGGAC | GAGAAACGTGCTCCTGGAAG |
| Mouse | *Dhcr24* | CATCTTCCGCTACCTCTTCG | CTCTGCTTCATCTCCCTTGG |
| Mouse | *Dhcr7* | CGCTCCCAAAGTCAAGAGTC | GTGTCTTGGCCCAAATGTCT |
| Mouse | *Lxrα* | TCTGGAGACGTCACGGAGGTA | CCCGGTTGTAACTGAAGTCCTT |
| Mouse | *LXRβ* | CTCCCACCCACGCTTACAC | GCCCTAACCTCTCTCCACTCA |
| Mouse | *Nrf2* | CGAGATATACGCAGGAGAGGTAAGA | GCTCGACAATGTTCTCCAGCTT |
| Mouse | *Atf4* | AGCAAAACAAGACAGCAGCC | ACTCTCTTCTTCCCCCTTGC |
| Mouse | *Sqle* | CCTGTTGGGTTGCTTTCAAT | CACGTGGACTCCCTTTCAAT |
| Mouse | *Accs* | TGGGACCTTGTGGACAGGAT | TCCAGCACTGACAGCCACTT |
| Mouse | *Mvd* | AAGCAGACGGGCAGTACAGT | CCTGGAGGTGTCATTGAGGT |
| Mouse | *Cyp51a1* | CTGGCTGGTCTTAGGCACTTG | GGCCTTCACTGGGAGTCTTC |
| Mouse | *Tm7sf2* | GGTCTTGAGACCATCCCGAC | CCTCTTGCTTTCAACCTCTTAGG |
| Mouse | *Msmo1* | CTCAACCCGCTGAACTTGGTT | CTGGGCATCTGTCCCAAAGA |
| Mouse | *Nsdhl* | AGGAGATGATGGCTGCTTGG | CTGTAACCCATTCTGCCCCA |
| Mouse | *Ebp* | GTGCCTAATGACCTCCCGAC | TTTCCCTCAGGTTCCTAGCAGT |
| Mouse | *Sc5dl* | GATCCTGCCTCCAAGTGACA | CATCGCAGCCCCTGGTAT |
| Mouse | *Srebf-1a* | GGCCGAGATGTGCGAACT | TTGTTGATGAGCTGGAGCATGT |
| Mouse | *Mgat* | GCCTTGCCACTGATATATGCC | TTCAA TCTGCTCTGAGGTCGG |

**Reference**

1. Chen L, Chen Q, Xie B, Quan C, Sheng Y, Zhu S, et al. Disruption of the AMPK-TBC1D1 nexus increases lipogenic gene expression and causes obesity in mice via promoting IGF1 secretion. Proc Natl Acad Sci U S A. 2016;113(26):7219-24. Epub 2016/06/17. doi: 10.1073/pnas.1600581113. PubMed PMID: 27307439; PubMed Central PMCID: PMCPMC4932950.

2. Gong XM, Li YF, Luo J, Wang JQ, Wei J, Wang JQ, et al. Gpnmb secreted from liver promotes lipogenesis in white adipose tissue and aggravates obesity and insulin resistance. Nat Metab. 2019;1(5):570-83. Epub 2019/05/01. doi: 10.1038/s42255-019-0065-4. PubMed PMID: 32694855.

3. Ding X, Jiang X, Tian R, Zhao P, Li L, Wang X, et al. RAB2 regulates the formation of autophagosome and autolysosome in mammalian cells. Autophagy. 2019;15(10):1774-86. Epub 2019/04/09. doi: 10.1080/15548627.2019.1596478. PubMed PMID: 30957628; PubMed Central PMCID: PMCPMC6735470.

4. Xiao J, Luo J, Hu A, Xiao T, Li M, Kong Z, et al. Cholesterol transport through the peroxisome-ER membrane contacts tethered by PI(4,5)P2 and extended synaptotagmins. Sci China Life Sci. 2019;62(9):1117-35. Epub 2019/05/31. doi: 10.1007/s11427-019-9569-9. PubMed PMID: 31144242.

5. Chen L, Ma MY, Sun M, Jiang LY, Zhao XT, Fang XX, et al. Endogenous sterol intermediates of the mevalonate pathway regulate HMG-CoA reductase degradation and SREBP-2 processing. J Lipid Res. 2019. Epub 2019/08/29. doi: 10.1194/jlr.RA119000201. PubMed PMID: 31455613.

6. Zong Y, Zhang CS, Li M, Wang W, Wang Z, Hawley SA, et al. Hierarchical activation of compartmentalized pools of AMPK depends on severity of nutrient or energy stress. Cell Res. 2019;29(6):460-73. Epub 2019/04/06. doi: 10.1038/s41422-019-0163-6. PubMed PMID: 30948787; PubMed Central PMCID: PMCPMC6796943.

7. Lu XY, Shi XJ, Hu A, Wang JQ, Ding Y, Jiang W, et al. Feeding induces cholesterol biosynthesis via the mTORC1-USP20-HMGCR axis. Nature. 2020. Epub 2020/11/13. doi: 10.1038/s41586-020-2928-y. PubMed PMID: 33177714.

8. Chen L, Chen Q, Rong P, Wang HY, Chen S. The energy sensing LKB1-AMPKalpha1 pathway regulates IGF1 secretion and consequent activation of the IGF1R-PKB pathway in primary hepatocytes. FEBS J. 2017;284(13):2096-109. Epub 2017/05/14. doi: 10.1111/febs.14106. PubMed PMID: 28500773.

9. Stark R, Grzelak M, Hadfield J. RNA sequencing: the teenage years. Nat Rev Genet. 2019;20(11):631-56. Epub 2019/07/26. doi: 10.1038/s41576-019-0150-2. PubMed PMID: 31341269.

10. Short B, Preisinger C, Korner R, Kopajtich R, Byron O, Barr FA. A GRASP55-rab2 effector complex linking Golgi structure to membrane traffic. J Cell Biol. 2001;155(6):877-83. Epub 2001/12/12. doi: 10.1083/jcb.200108079. PubMed PMID: 11739401; PubMed Central PMCID: PMCPMC2150909.

11. Li BT, Sun M, Li YF, Wang JQ, Zhou ZM, Song BL, et al. Disruption of the ERLIN-TM6SF2-APOB complex destabilizes APOB and contributes to non-alcoholic fatty liver disease. PLoS Genet. 2020;16(8):e1008955. Epub 2020/08/11. doi: 10.1371/journal.pgen.1008955. PubMed PMID: 32776921; PubMed Central PMCID: PMCPMC7462549.

12. Chen Q, Rong P, Xu D, Zhu S, Chen L, Xie B, et al. Rab8a Deficiency in Skeletal Muscle Causes Hyperlipidemia and Hepatosteatosis by Impairing Muscle Lipid Uptake and Storage. Diabetes. 2017;66(9):2387-99. Epub 2017/07/12. doi: 10.2337/db17-0077. PubMed PMID: 28696211.

13. Wu L, Xu D, Zhou L, Xie B, Yu L, Yang H, et al. Rab8a-AS160-MSS4 regulatory circuit controls lipid droplet fusion and growth. Dev Cell. 2014;30(4):378-93. Epub 2014/08/28. doi: 10.1016/j.devcel.2014.07.005. PubMed PMID: 25158853.
