# Supplementary material for: Rab2A regulates the progression of nonalcoholic fatty liver disease downstream of AMPK-TBC1D1 axis by stabilizing PPARγ
Source: PLoS Biol. 2022 Jan 21;20(1):e3001522. doi: 10.1371/journal.pbio.3001522 (PMC8809606; doi:10.1371/journal.pbio.3001522)
Supplement: S1 Raw images — (PDF) [file pbio.3001522.s012.pdf]

Figure 1F

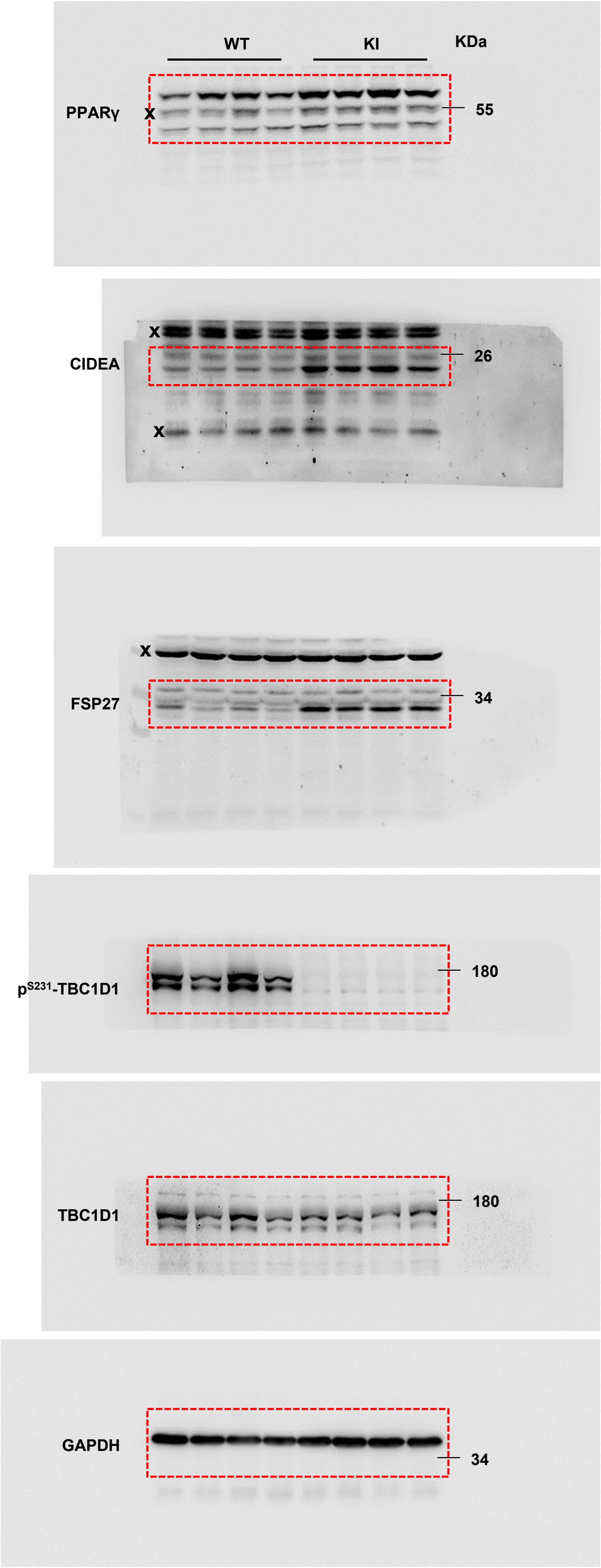

Chemiluminescence Western Blotting,
Autoradiography machine (Tanon-5200) detection

Figure 2A

HA-Vector	+	+	+	-	-	-	-	-	-
HA-TBC1D1^{WT}	-	-	-	+	+	+	-	-	-
HA-TBC1D1^{S237A}	-	-	-	-	-	-	+	+	+
PPARγ2-MYC	+	+	+	+	+	+	+	+	+
A769662(μM)	0	10	25	0	10	25	0	10	25

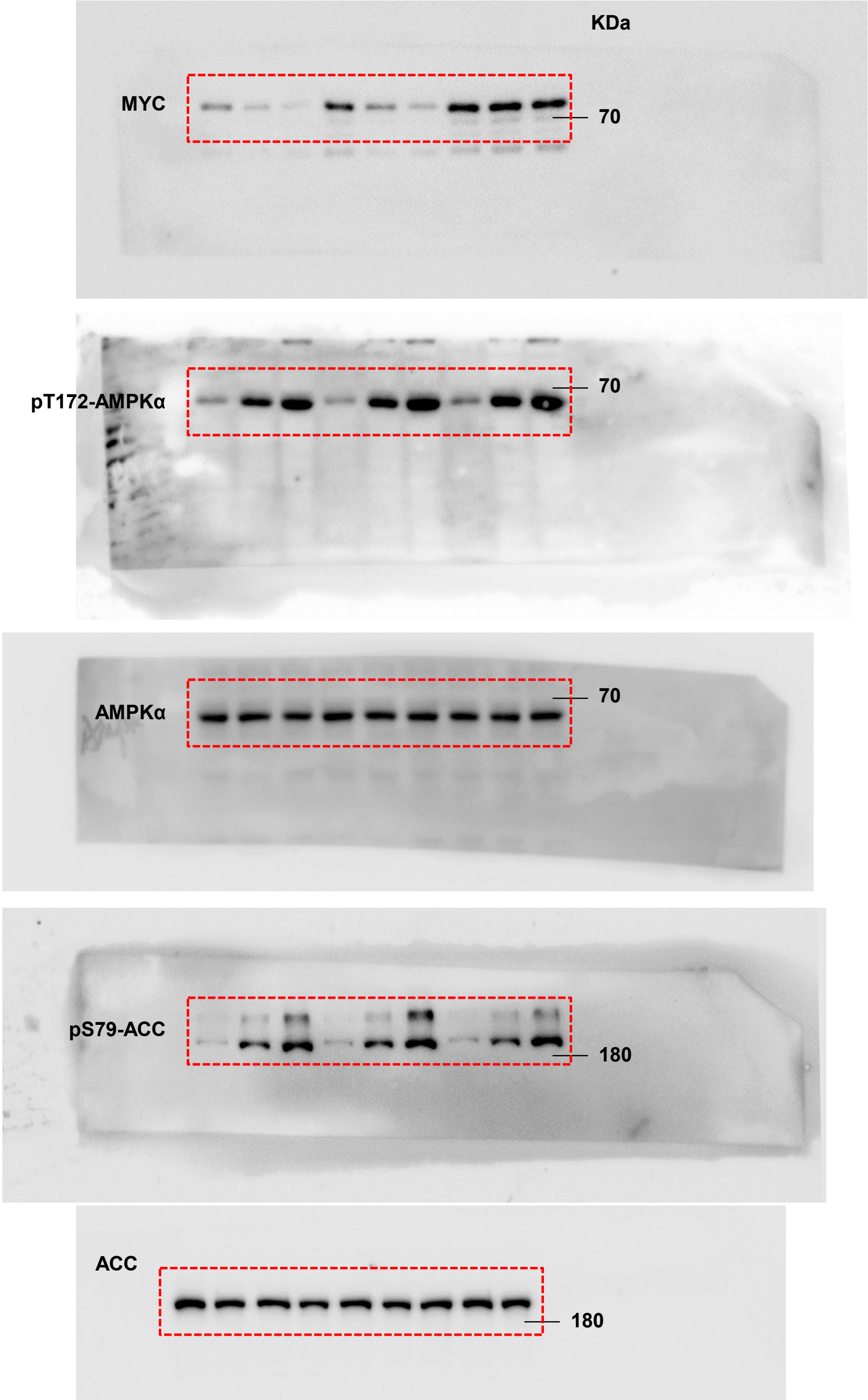

Chemiluminescence Western Blotting,
Autoradiography machine (Tanon-5200) detection

Figure 2A

HA-Vector	+	+	+	-	-	-	-	-	-
HA-TBC1D1^{WT}	-	-	-	+	+	+	-	-	-
HA-TBC1D1^{S237A}	-	-	-	-	-	-	+	+	+
PPARγ2-MYC	+	+	+	+	+	+	+	+	+
A769662(μM)	0	10	25	0	10	25	0	10	25

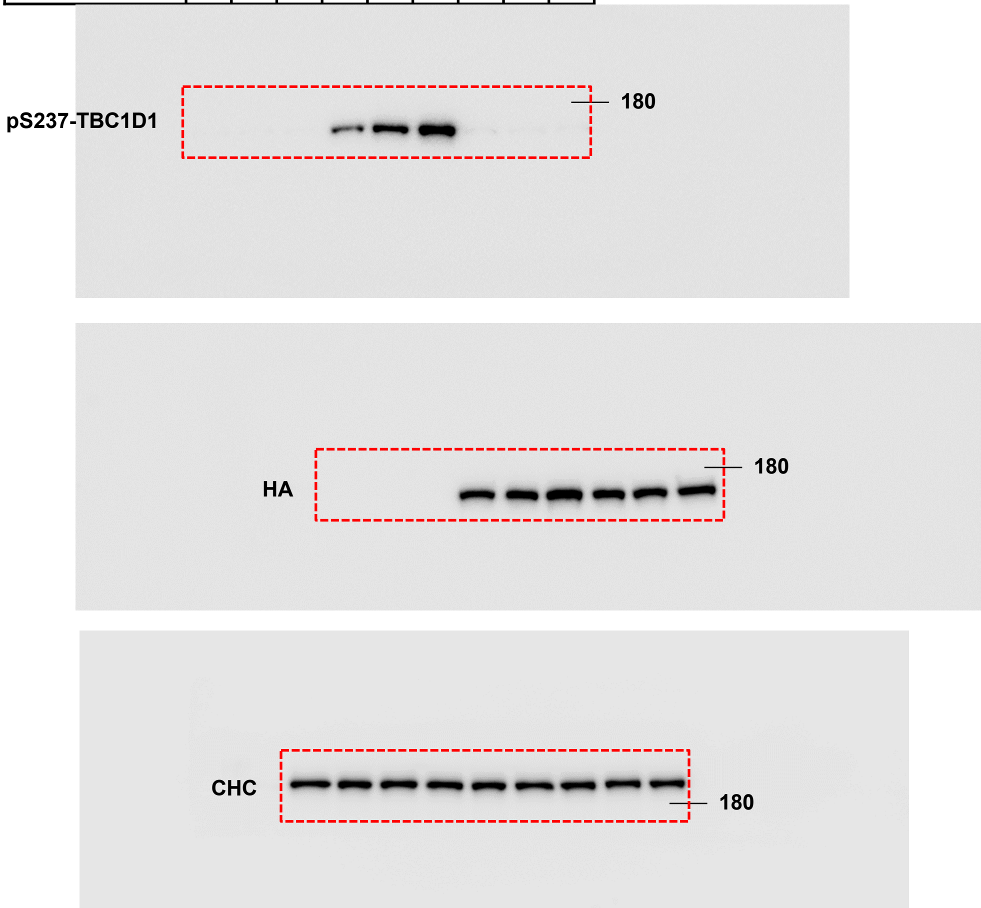

Chemiluminescence Western Blotting,
Autoradiography machine (Tanon-5200) detection

Figure 2B

HA-Vector	+	+	+	-	-	-	-	-	-
HA-TBC1D1^{WT}	-	-	-	+	+	+	-	-	-
HA-TBC1D1^{S237A}	-	-	-	-	-	-	+	+	+
A769662(μM)	0	10	25	0	10	25	0	10	25

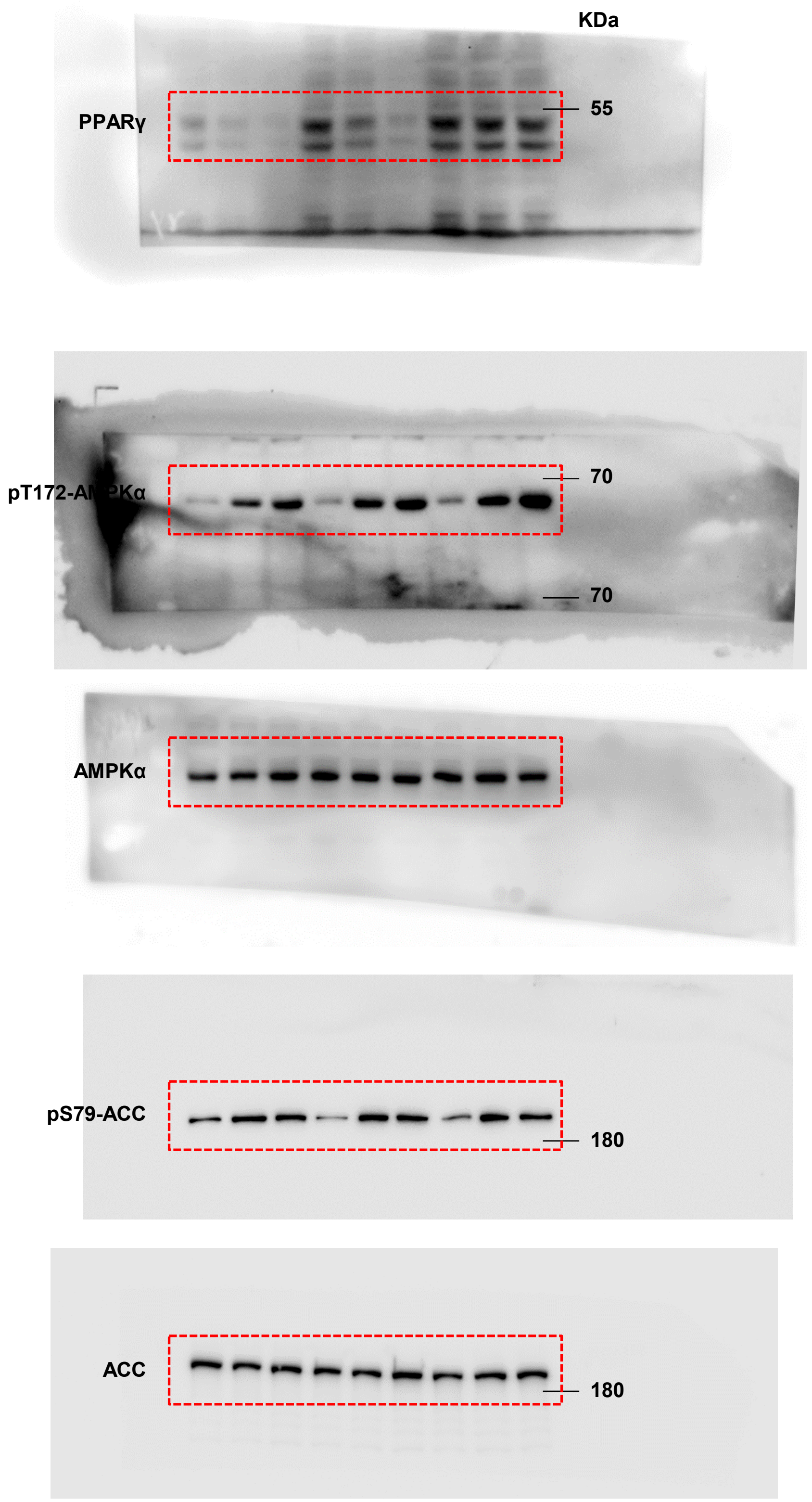

Chemiluminescence Western Blotting,
Autoradiography machine (Tanon-5200) detection

Figure 2B

HA-Vector	+	+	+	-	-	-	-	-	-
HA-TBC1D1^{WT}	-	-	-	+	+	+	-	-	-
HA-TBC1D1^{S237A}	-	-	-	-	-	-	+	+	+
A769662(μM)	0	10	25	0	10	25	0	10	25

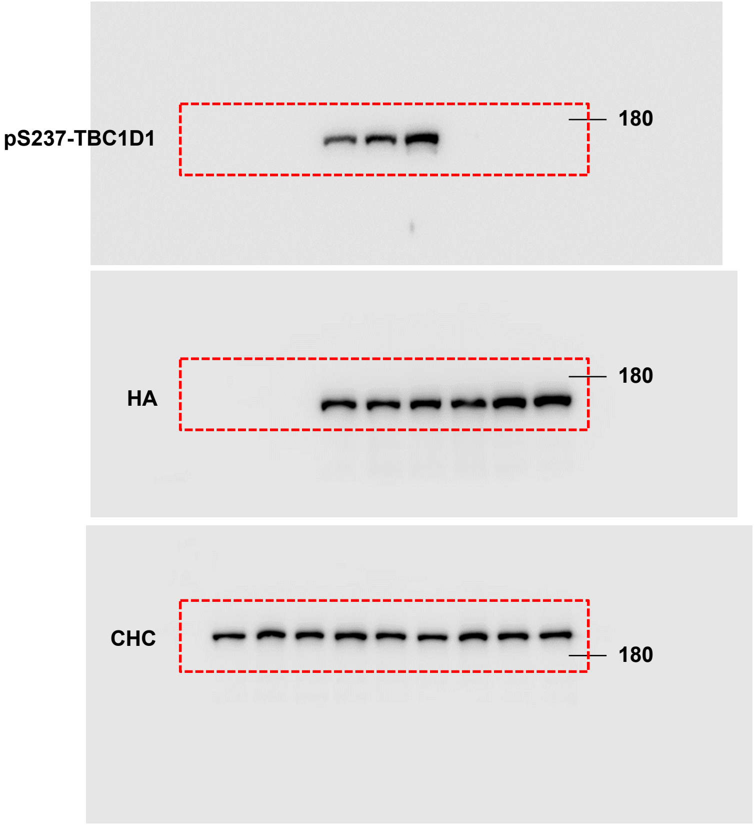

Chemiluminescence Western Blotting,
Autoradiography machine (Tanon-5200) detection

Figure 3A

GST-PPARγ2	+	+	-	+	-
Flag-TBC1D1^{WT}	-	+	+	-	-
Flag-TBC1D1^{S237A}	-	-	-	+	+

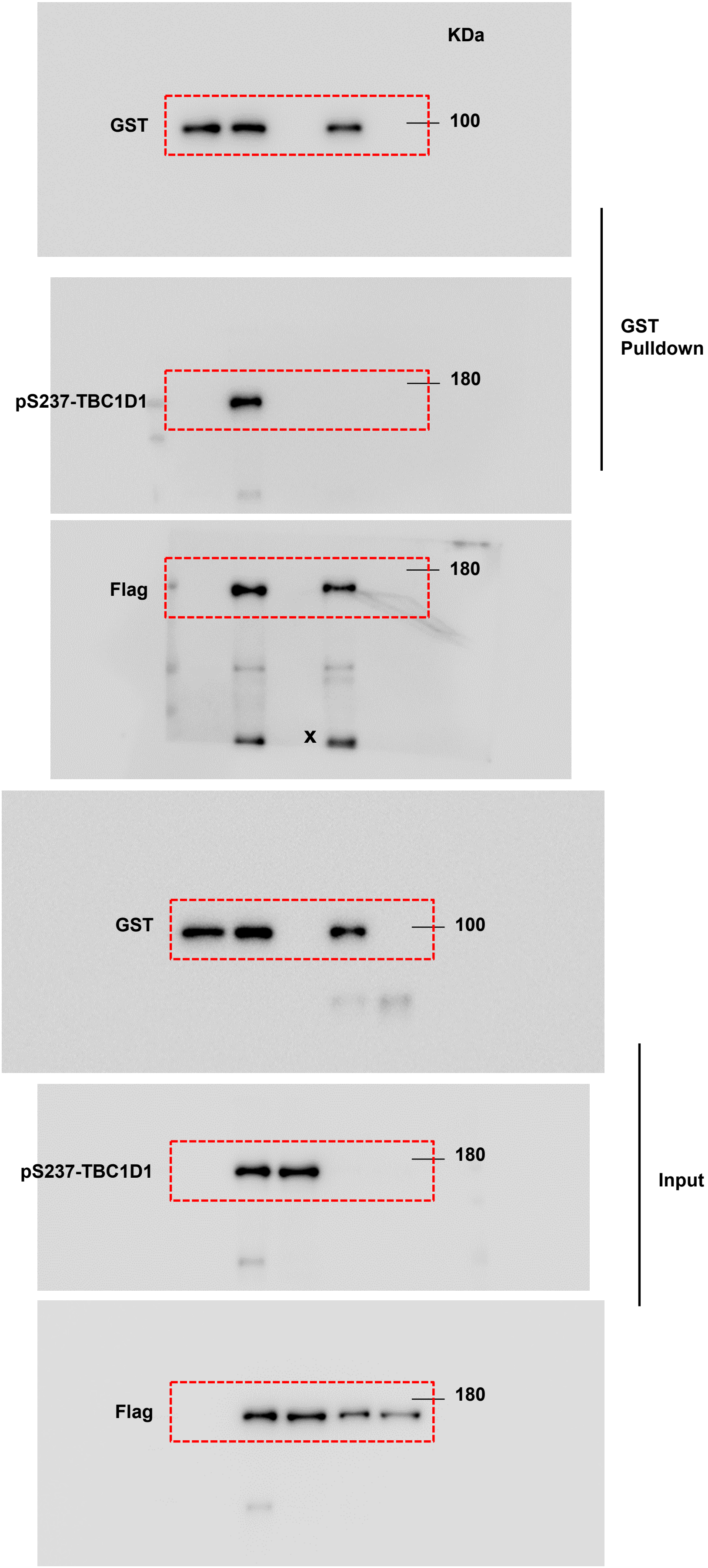

Chemiluminescence Western Blotting,
Autoradiography machine (Tanon-5200) detection

Figure 3B

HA-Vector	+	-	-	-	-
HA-TBC1D1	-	+	-	-	-
HA-TBC1D1^{S237A}	-	-	+	-	-
HA-TBC1D1$^{S237A/R854K}$	-	-	-	+	-
HA-TBC1D1^{R854K}	-	-	-	-	+
PPARγ2-MYC	+	+	+	+	+

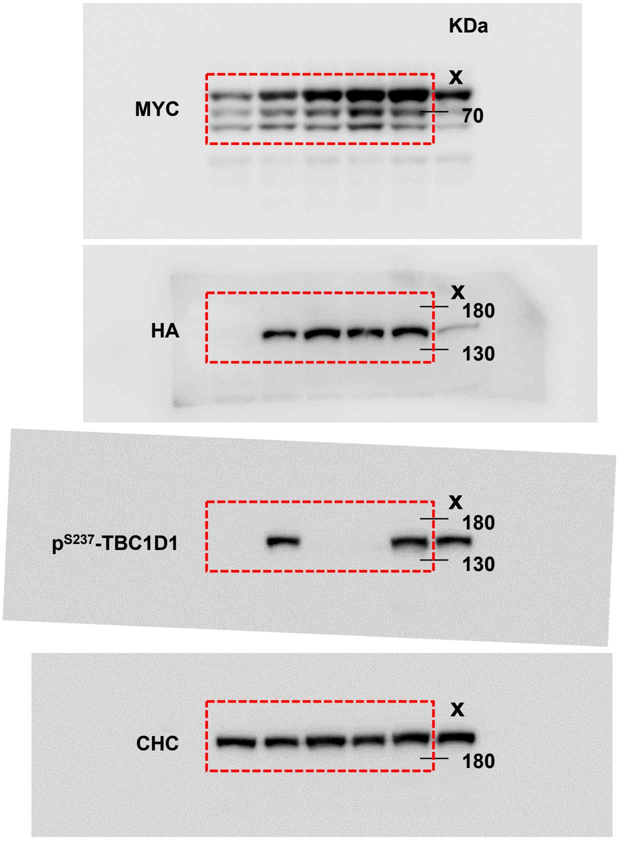

Chemiluminescence Western Blotting,
Autoradiography machine (Tanon-5200) detection

Figure 3C

Flag-Vector	+	-
Flag-Rab2A	-	+

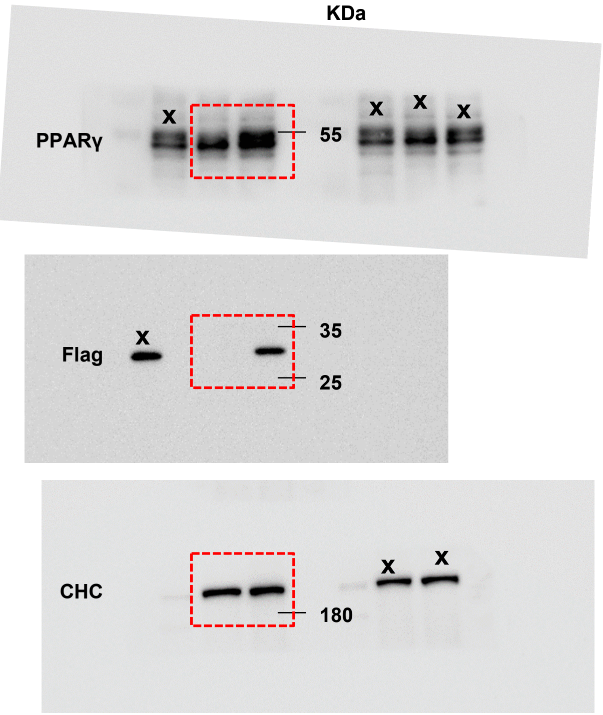

Figure 3D

shRNA-NC	+	-
shRNA-Rab2A	-	+

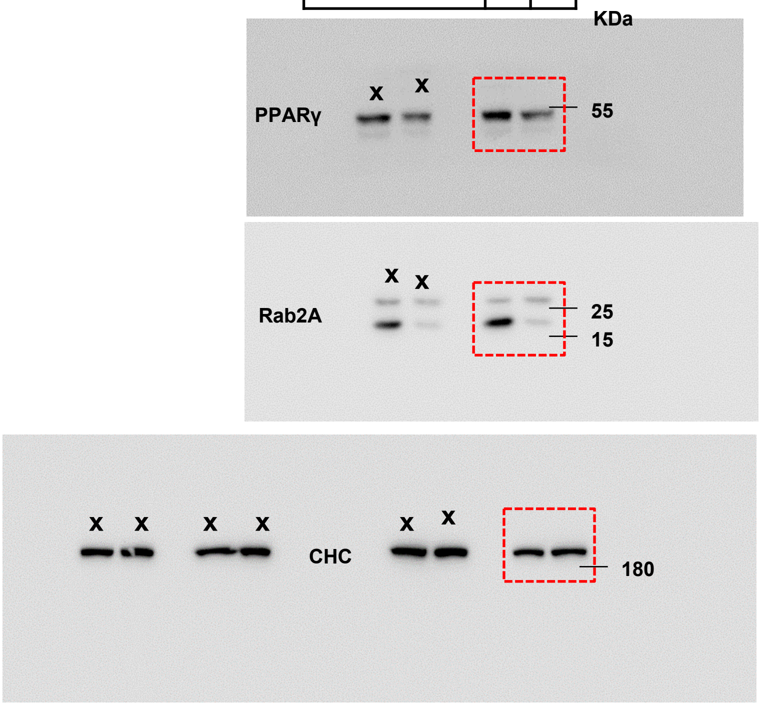

Chemiluminescence Western Blotting,
Autoradiography machine (Tanon-5200) detection

Figure 3E

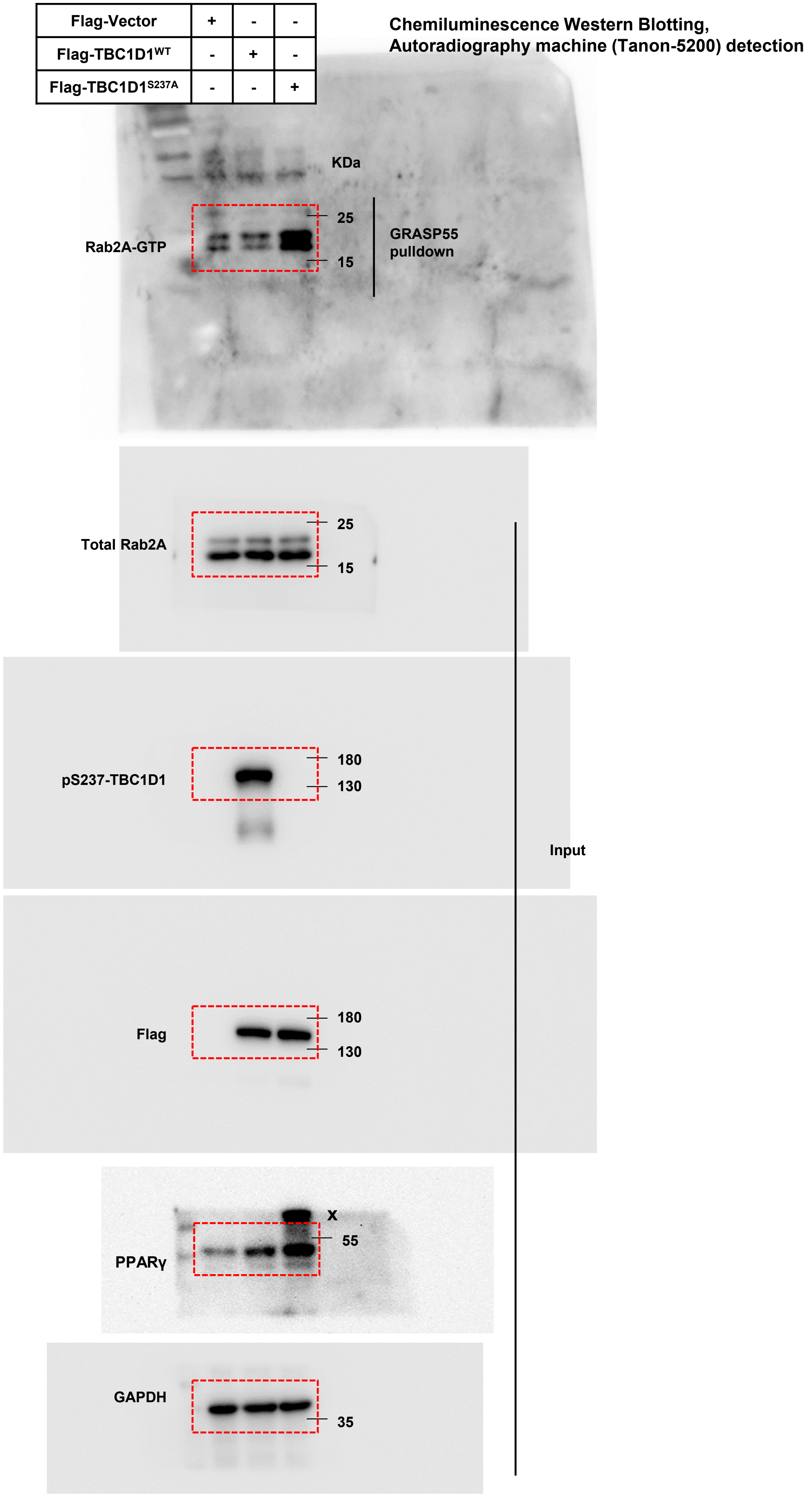

Flag-Vector + - -
Flag-TBC1D1WT - + -
Flag-TBC1D1S237A - - +
Chemiluminescence Western Blotting,
Autoradiography machine (Tanon-5200) detection
KDa
Rab2A-GTP
25
15
GRASP55
pulldown
Total Rab2A
25
15
pS237-TBC1D1
180
130
Input
Flag
180
130
PPARγ
x
55
GAPDH
35

Figure 3F

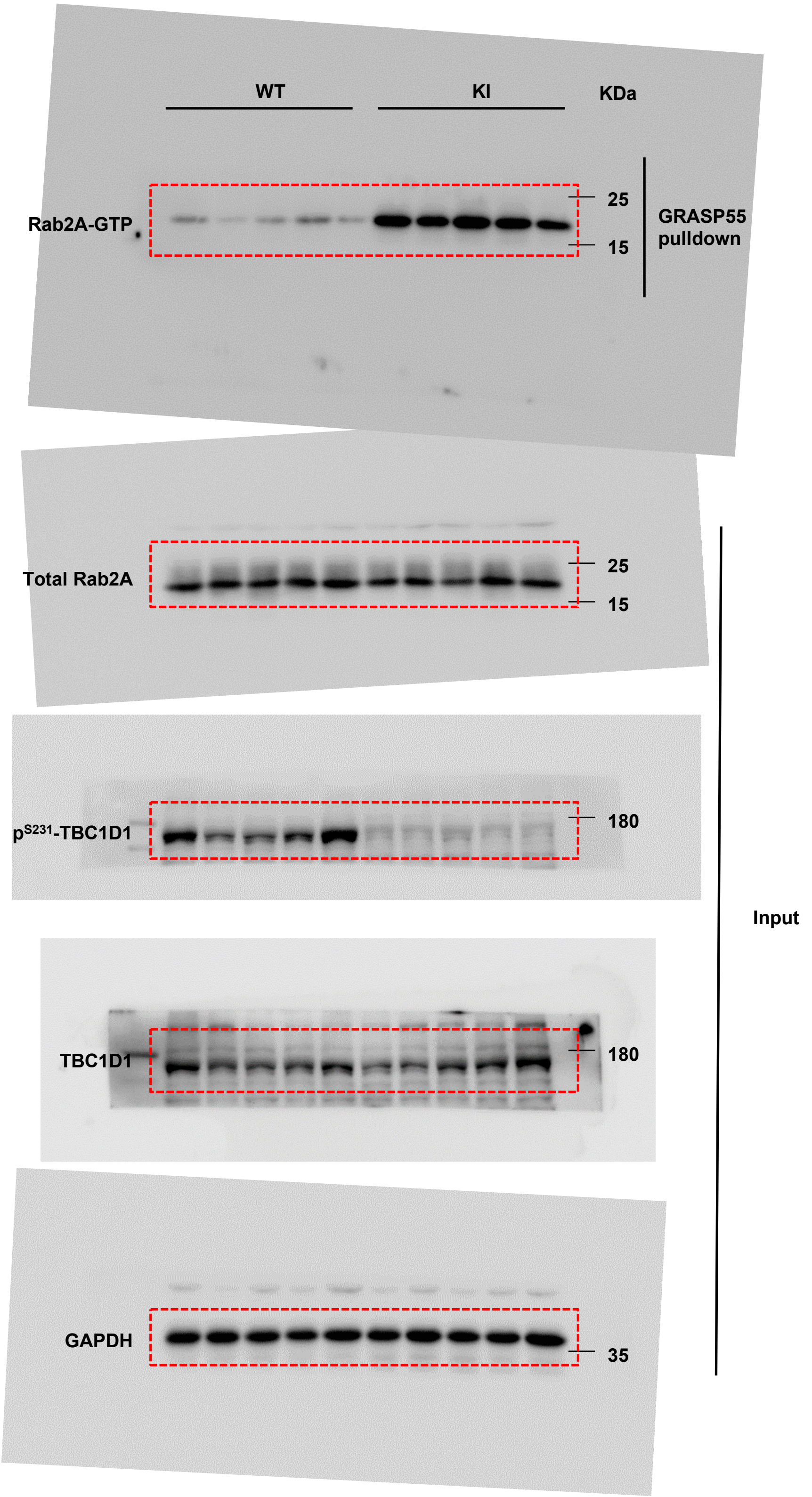

Chemiluminescence Western Blotting,
Autoradiography machine (Tanon-5200) detection

Figure 3H

HA-Vector	+	-	-	-	-
HA-TBC1D1^{WT}	-	+	+	-	-
HA-TBC1D1^{S237A}	-	-	-	+	+
SiRNA-NC	+	+	-	+	-
SiRNA-Rab2A	-	-	+	-	+
PPARγ2-MYC	+	+	+	+	+

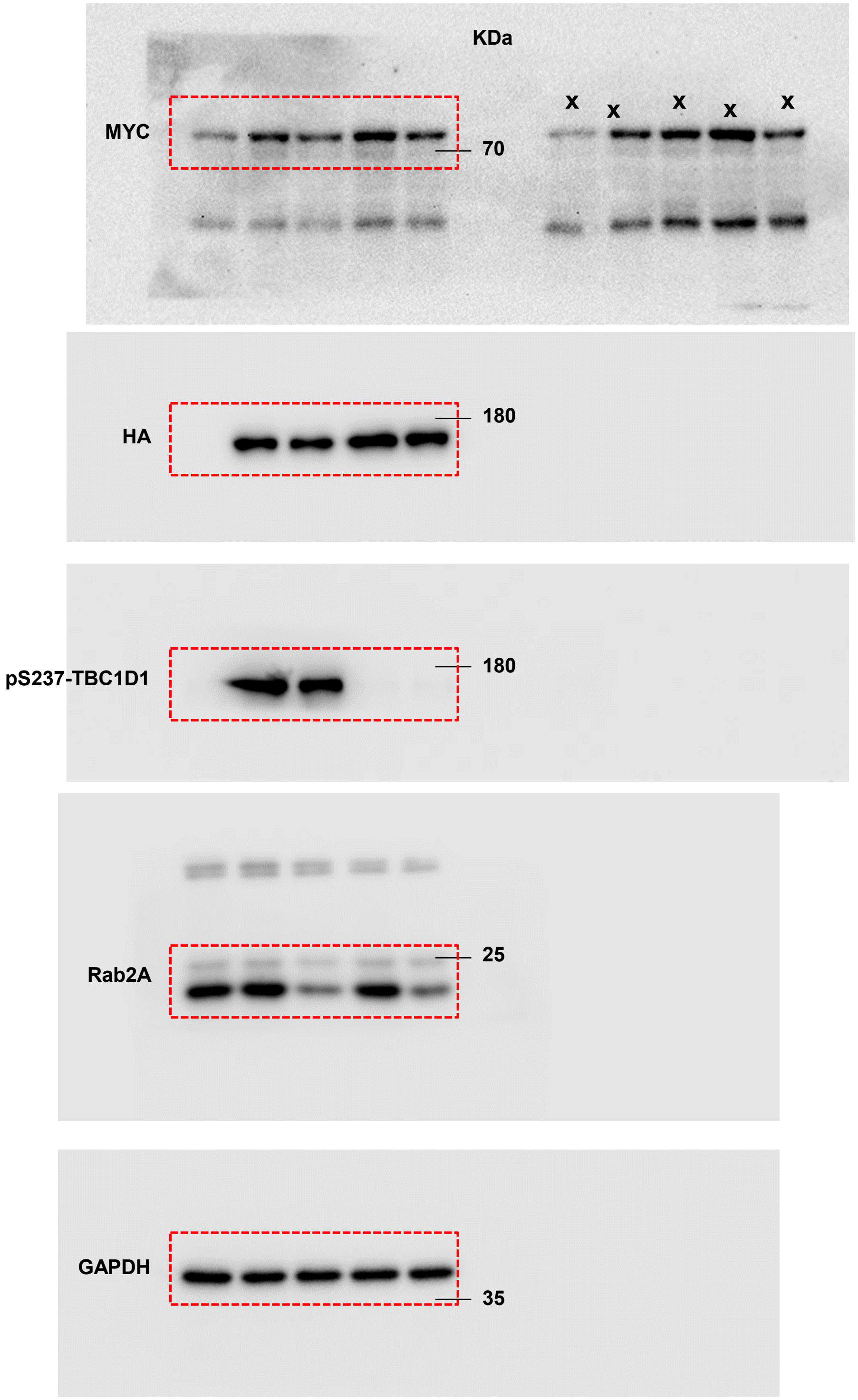

Chemiluminescence Western Blotting,
Autoradiography machine (Tanon-5200) detection

Figure 4A

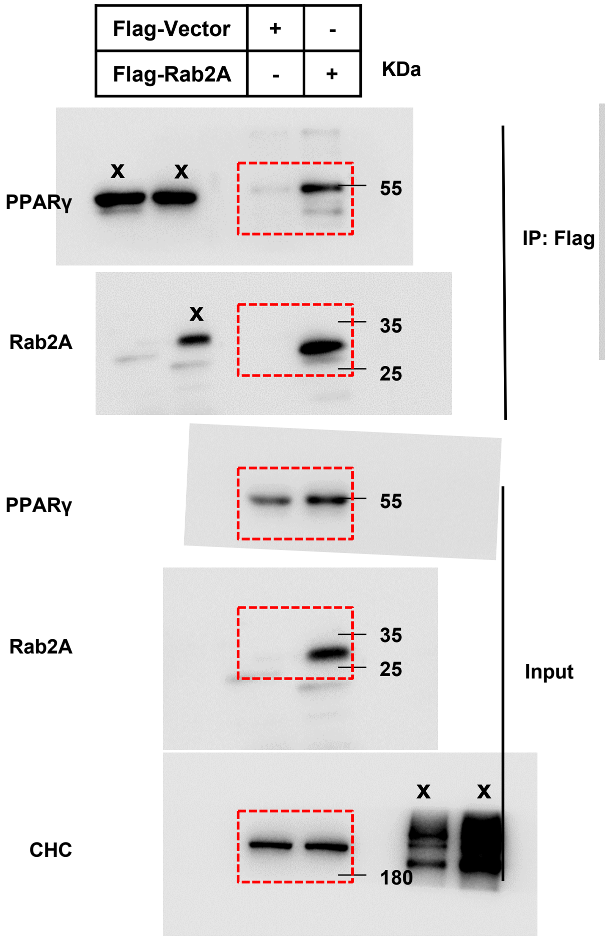

Figure 4B

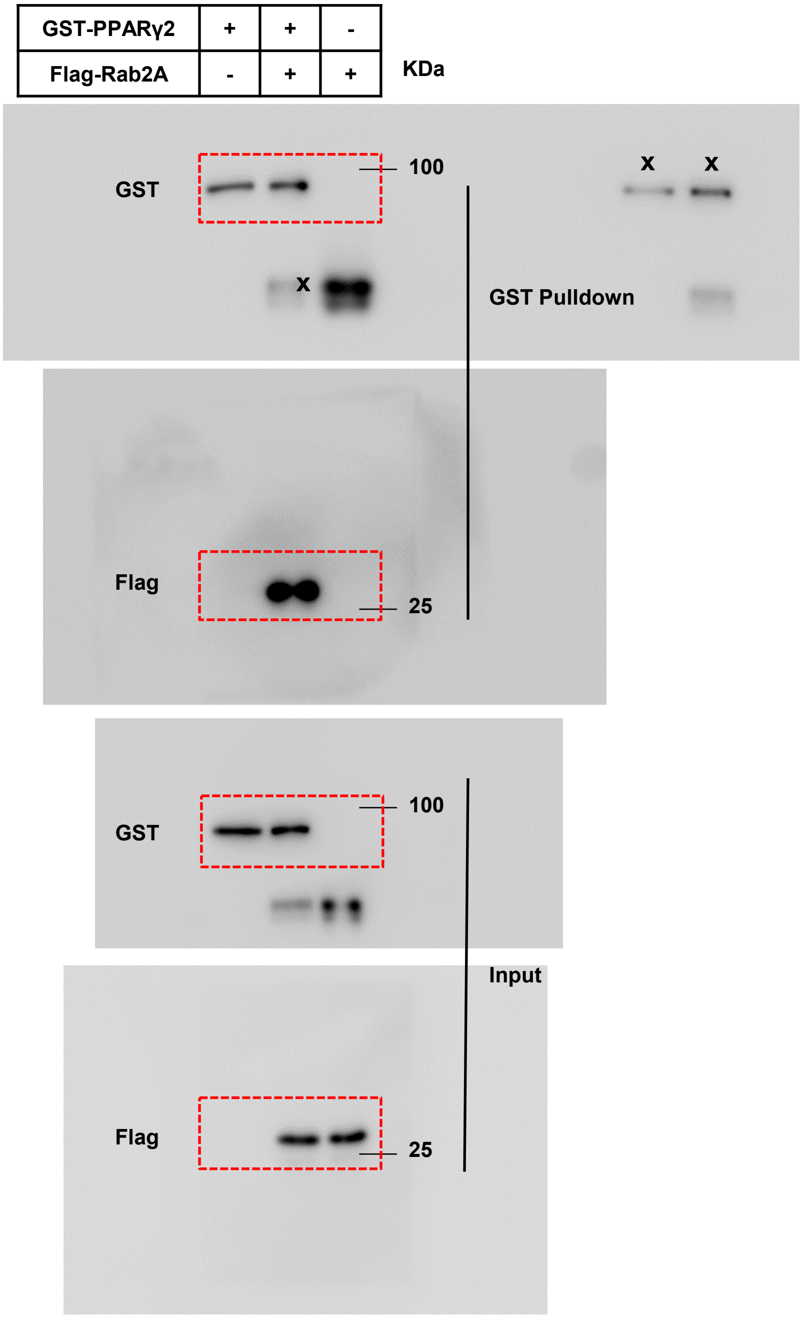

Chemiluminescence Western Blotting,
Autoradiography machine (Tanon-5200) detection

Figure 4D

Flag-Vector	+	-	-	-
Flag-Rab2A^{WT}	-	+	-	-
Flag-Rab2A^{Q65L}	-	-	+	-
Flag-Rab2A^{S20N}	-	-	-	+

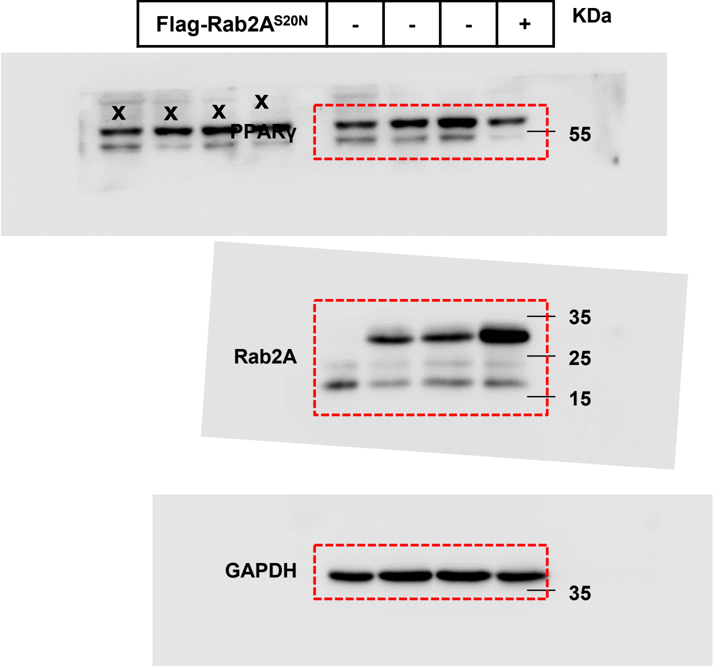

Chemiluminescence Western Blotting,
Autoradiography machine (Tanon-5200) detection

Figure 4E

Flag-Vector	+	-	-	-
Flag-Rab2A^{WT}	-	+	-	-
Flag-Rab2A^{Q65L}	-	-	+	-
Flag-Rab2A^{S20N}	-	-	-	+
PPARγ2-MYC	+	+	+	+

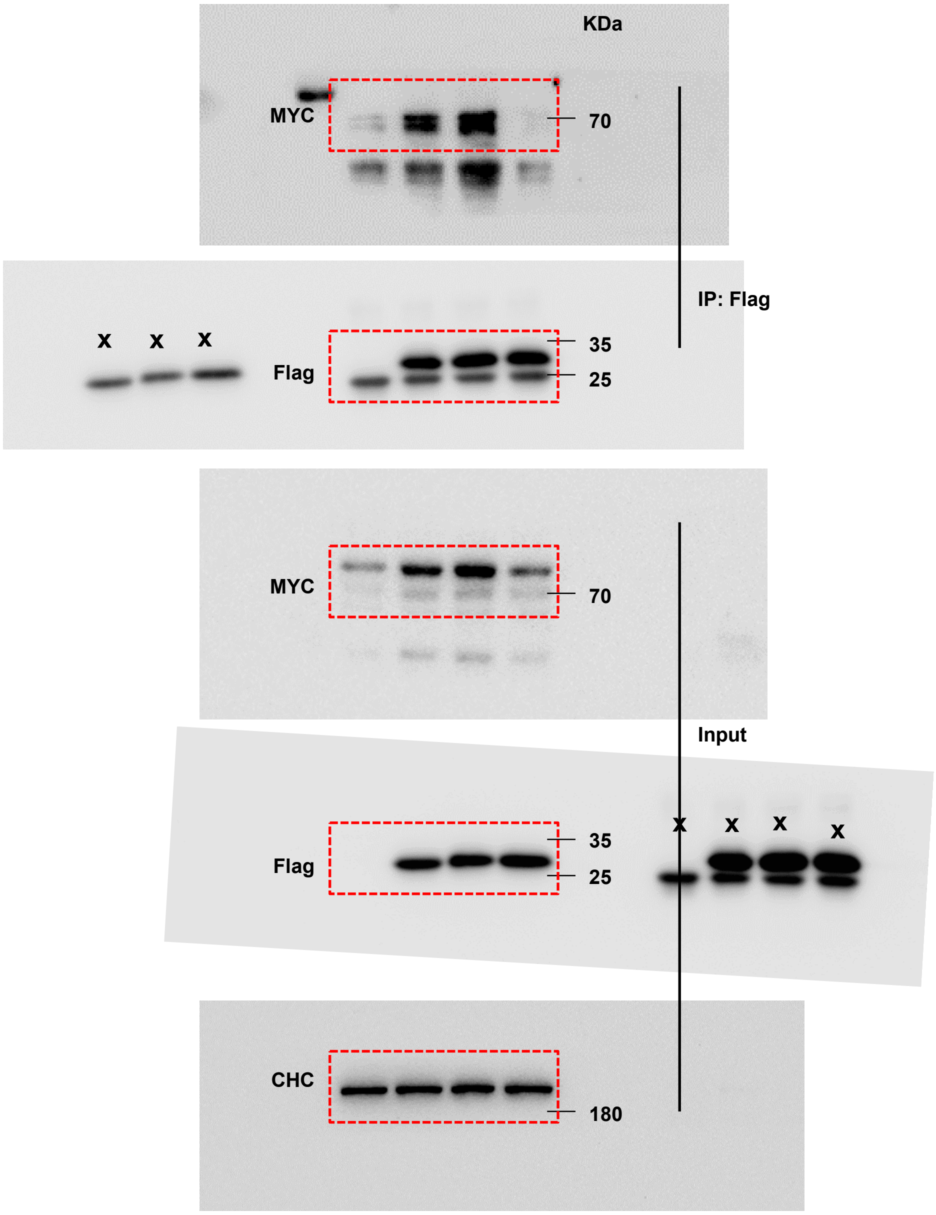

Chemiluminescence Western Blotting,
Autoradiography machine (Tanon-5200) detection

Figure 4F

Flag-Vector	+	-	+	-
Flag-Rab2A^{Q65L}	-	+	-	+
PPARγ2-MYC:FL	+	+	-	-
PPARγ2-MYC:T05	-	-	+	+

KDa

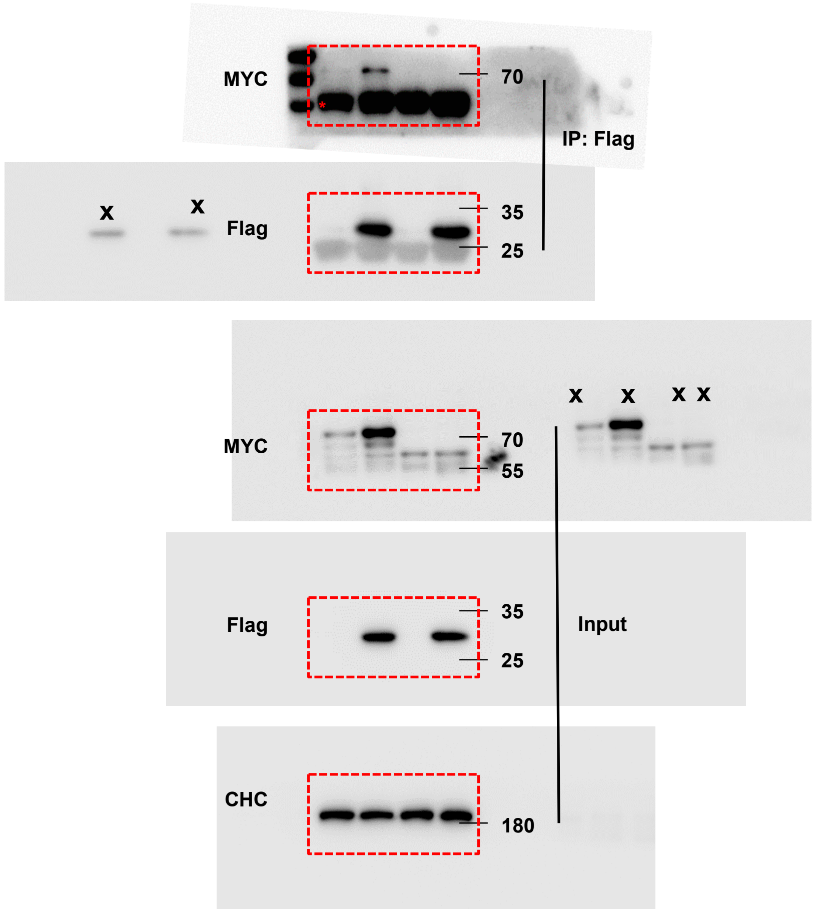

Chemiluminescence Western Blotting,
Autoradiography machine (Tanon-5200) detection

Figure 4G

Flag-Vector	+					-				
Flag-Rab2A	-					+				
PPARγ2-MYC	+									
MG132 (μM)	-	2.5	5.0	10	20	-	2.5	5.0	10	20

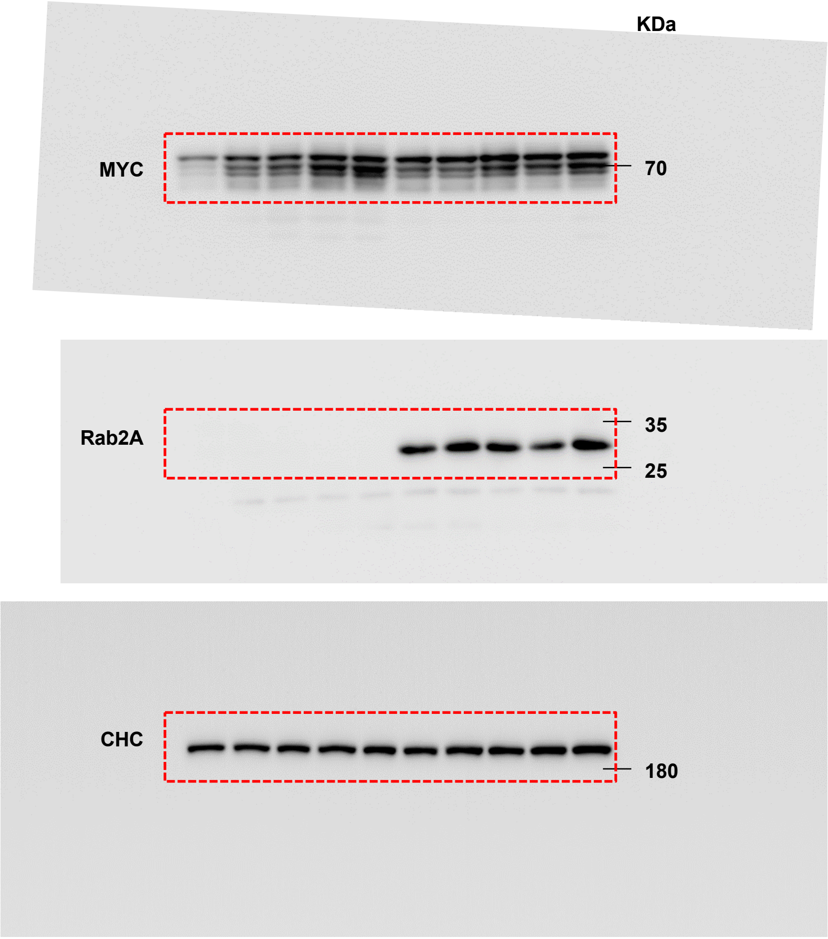

Chemiluminescence Western Blotting,
Autoradiography machine (Tanon-5200) detection

Figure 4I

Flag-Vector	+			-		
Flag-Rab2A	-			+		
PPARγ2-MYC	+					
MG132 (20 μM)	0	2	4	0	2	4

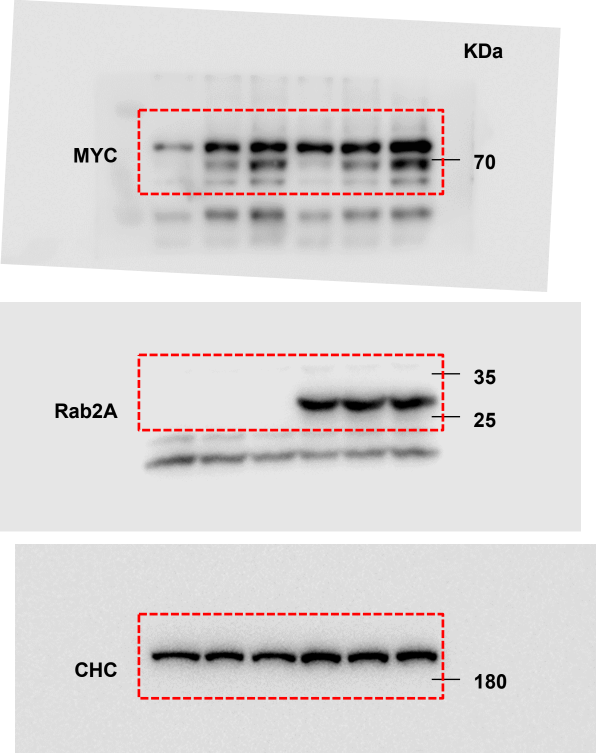

Figure 4K

Flag-Vector	+				-			
Flag-Rab2A	-				+			
PPARγ2-MYC	+							
ALLN (μg/ml)	-	1	3	10	-	1	3	10

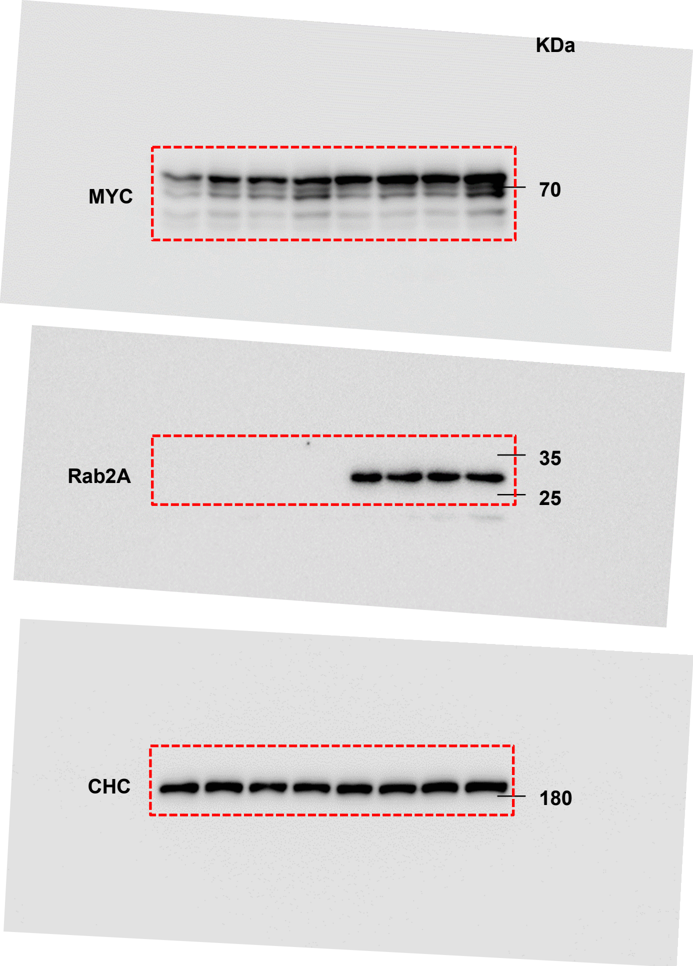

Chemiluminescence Western Blotting, Autoradiography machine (Tanon-5200) detection

Figure 4M

Flag-Vector	+				-			
Flag-Rab2A	-				+			
PPARγ2-MYC	+							
ALLN (10μg/ml)	-	2	4	6	-	2	4	6

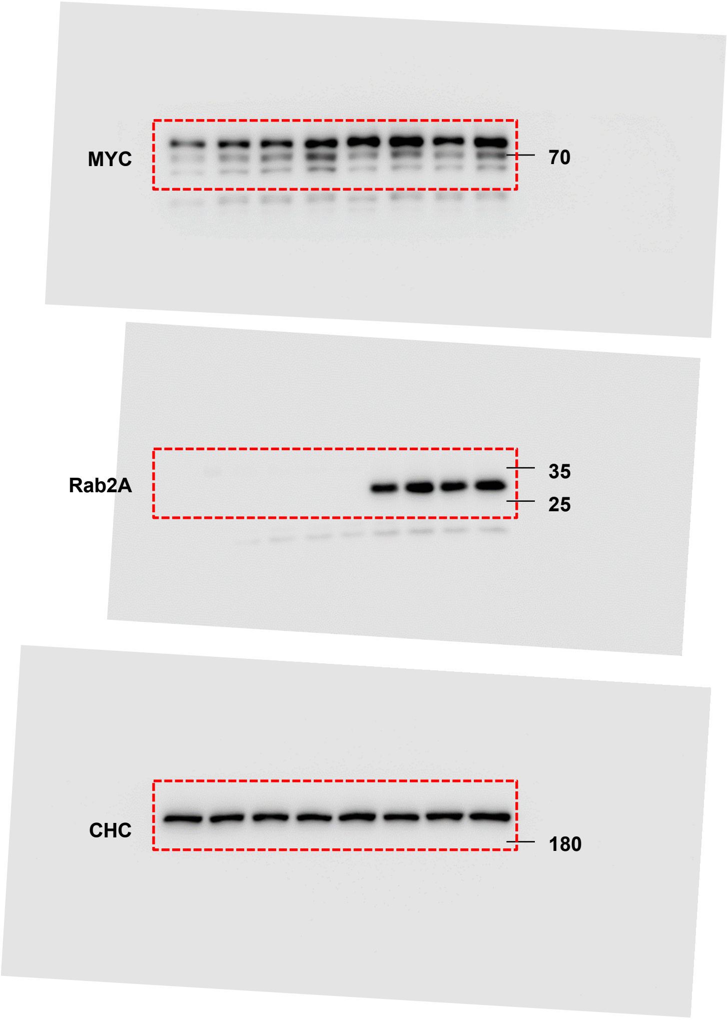

Chemiluminescence Western Blotting, Autoradiography machine (Tanon-5200) detection

Figure 5A

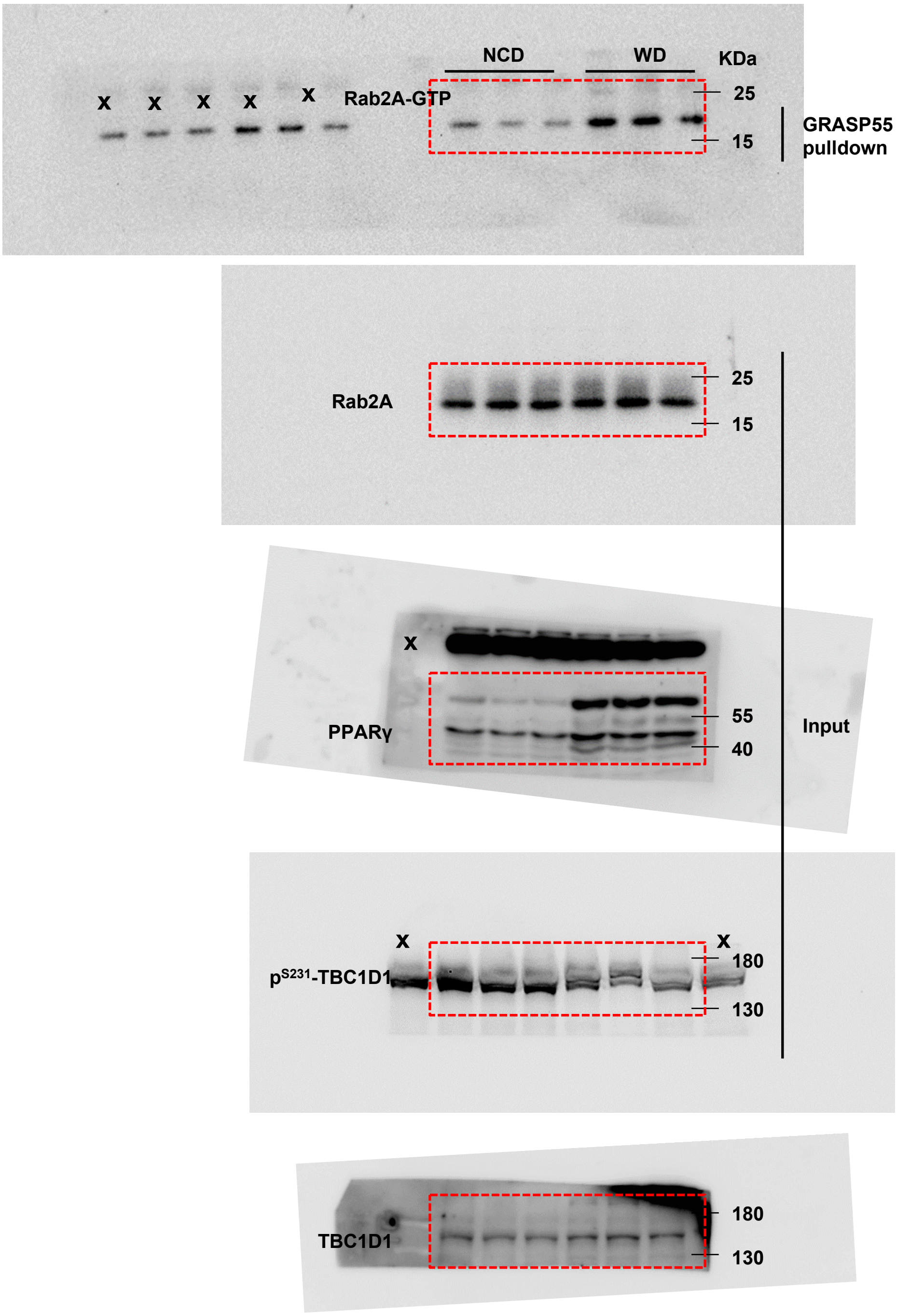

Chemiluminescence Western Blotting, Autoradiography machine (Tanon-5200) detection

Figure 5A

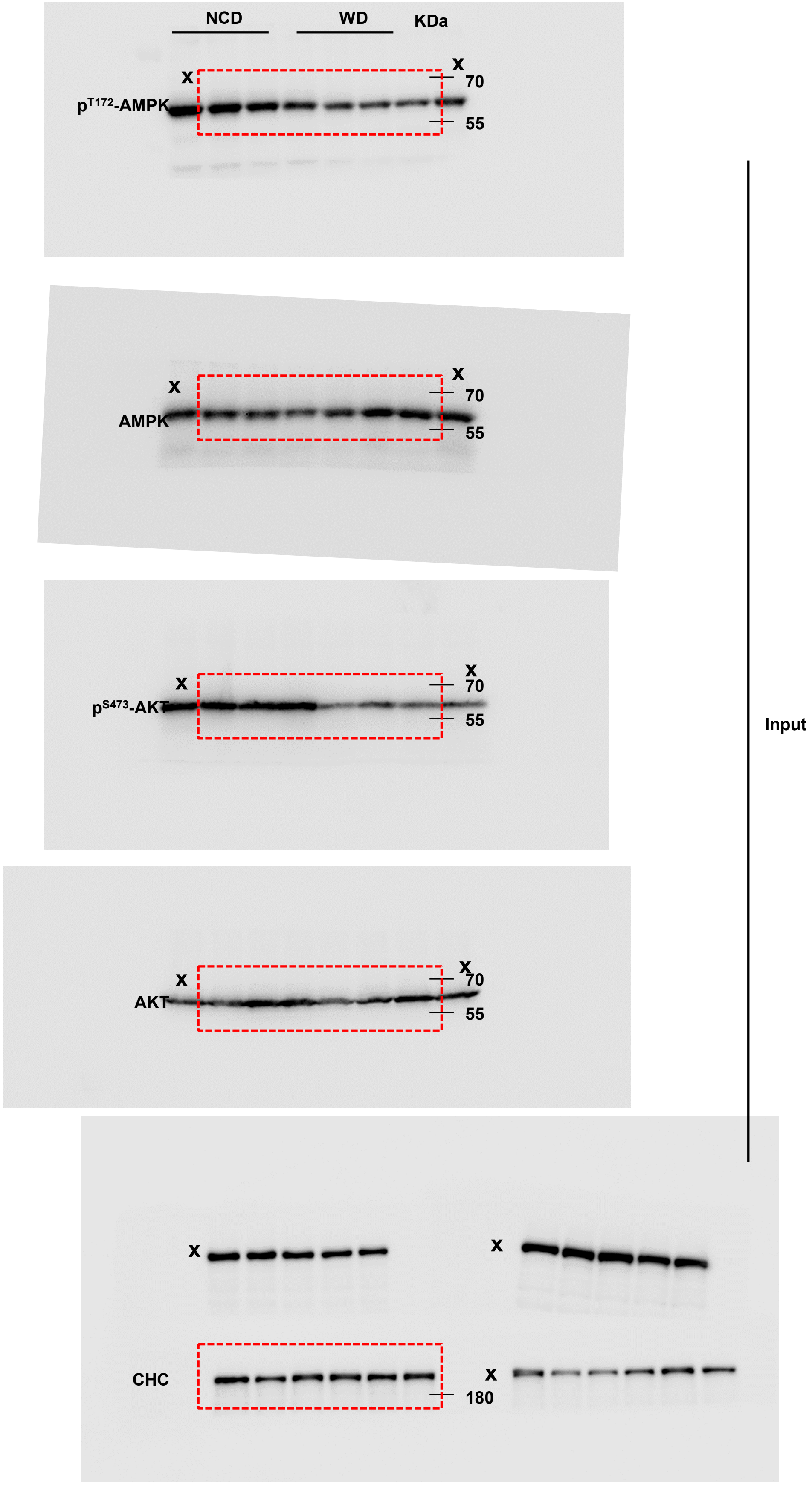

Chemiluminescence Western Blotting,
Autoradiography machine (Tanon-5200) detection

Figure 5D

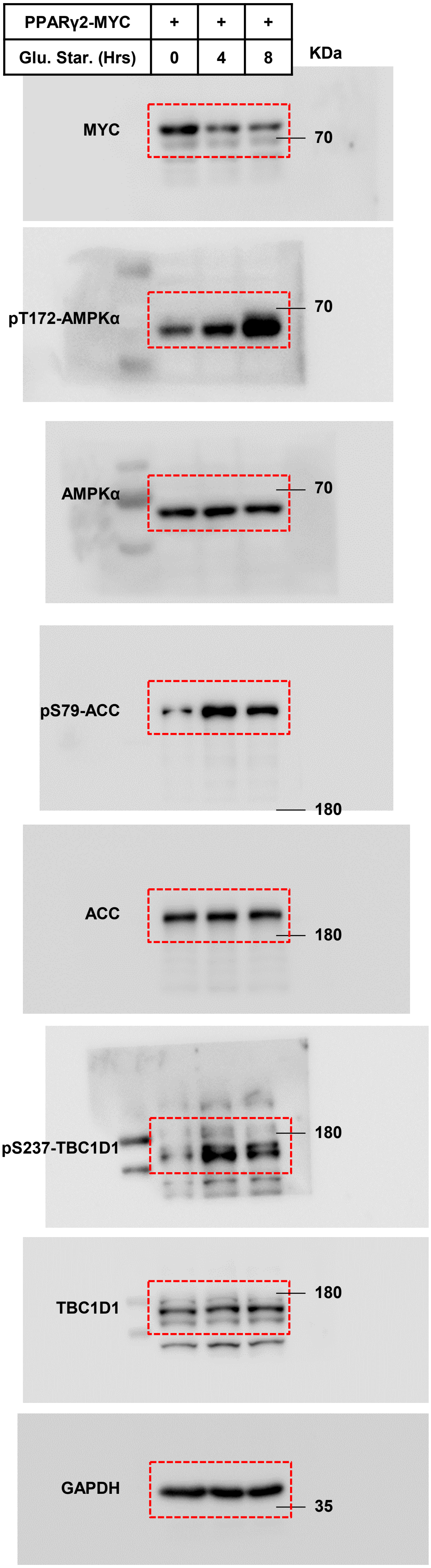

Chemiluminescence Western Blotting,
Autoradiography machine (Tanon-5200) detection

Figure 5E

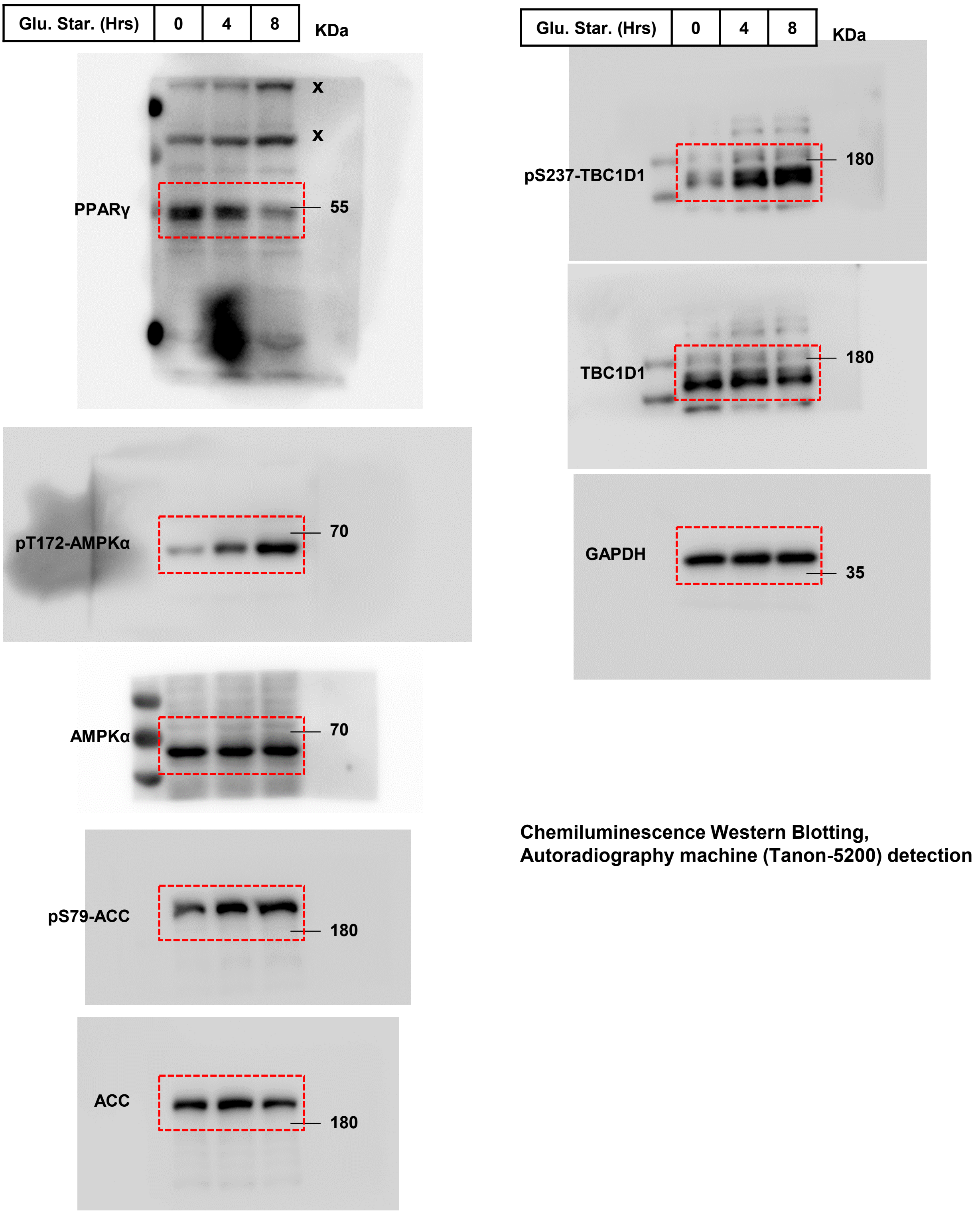
Glu. Star. (Hrs)
0
4
8
KDa
x
x
PPARγ
55
pT172-AMPKα
70
AMPKα
70
pS79-ACC
180
ACC
180
Glu. Star. (Hrs)
0
4
8
KDa
pS237-TBC1D1
180
TBC1D1
180
GAPDH
35
Chemiluminescence Western Blotting,
Autoradiography machine (Tanon-5200) detection

Figure 5F

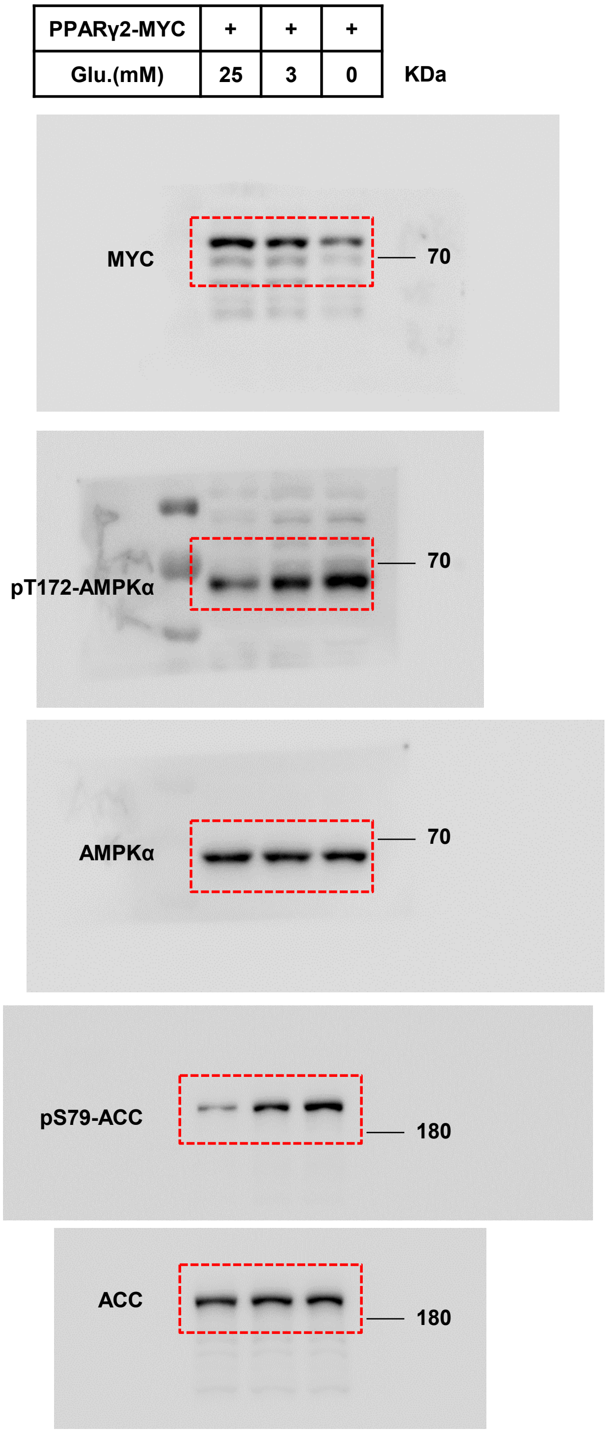

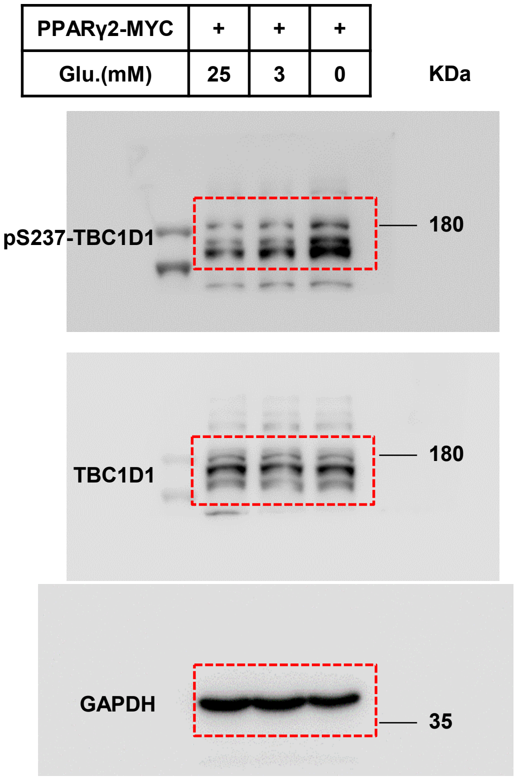

Chemiluminescence Western Blotting,
Autoradiography machine (Tanon-5200) detection

Figure 5G

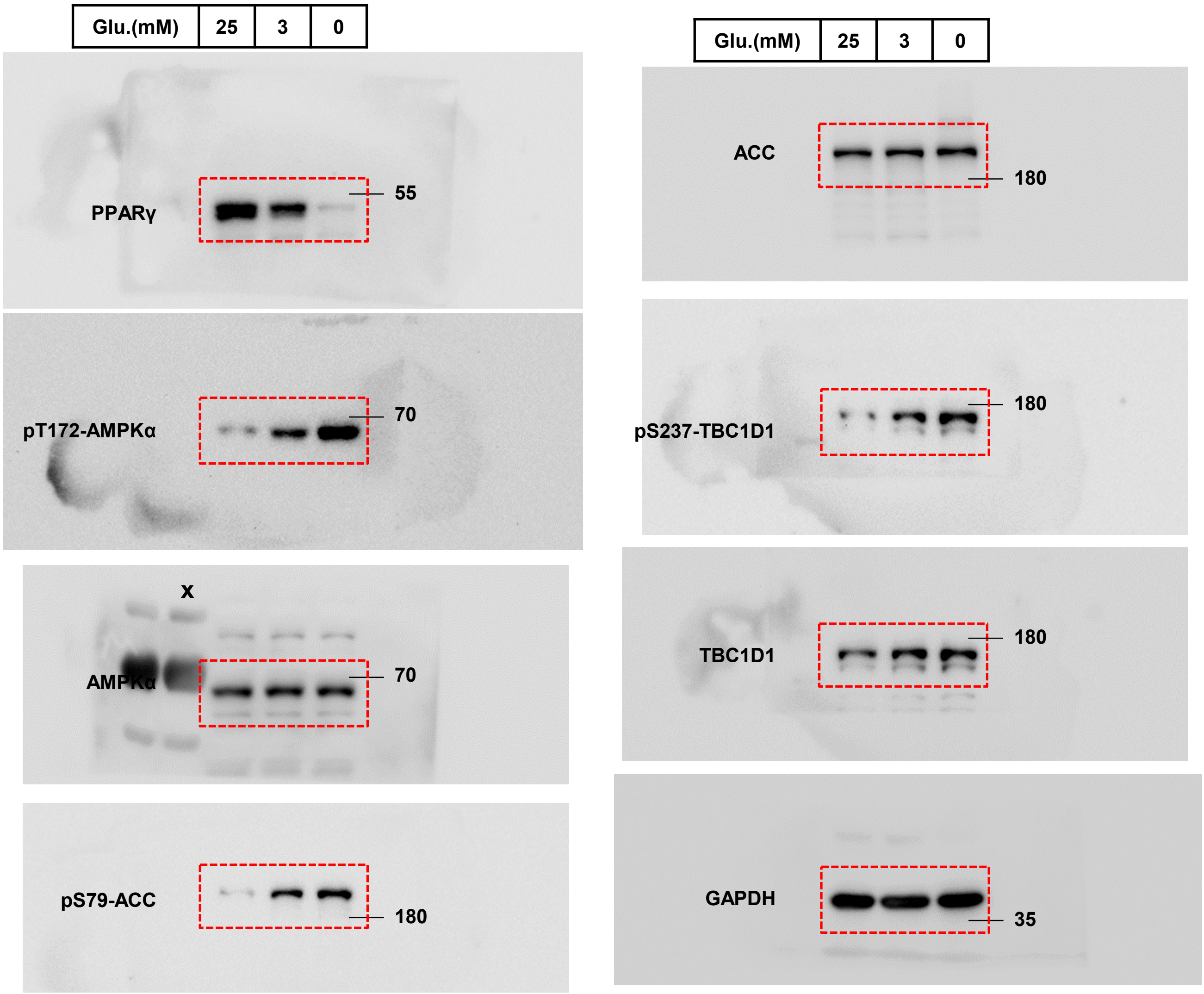

Chemiluminescence Western Blotting,
Autoradiography machine (Tanon-5200) detection

Figure 5H

PPARγ2-MYC	+	+	+	+	+
Glu.(mM)	25	0	0	0	0
Compound C(μM)	0	0	1.25	2.5	5.0

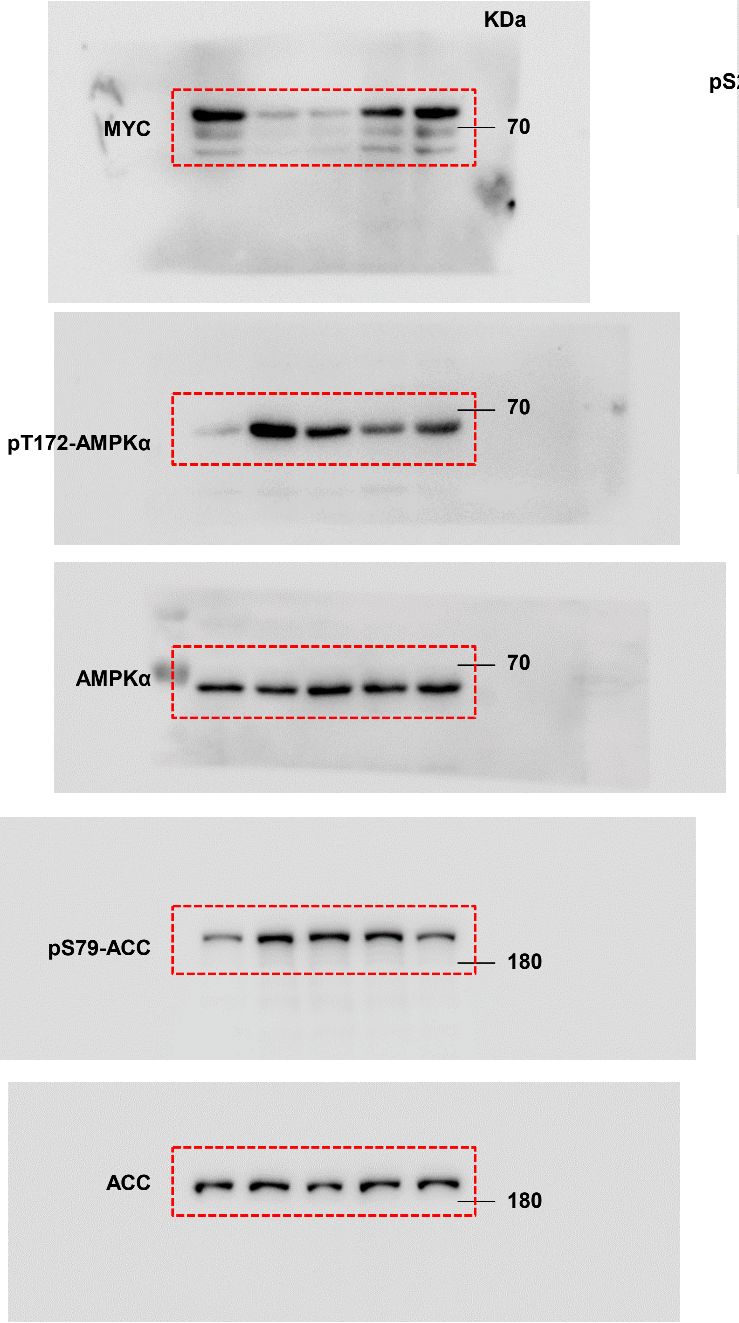

PPARγ2-MYC	+	+	+	+	+
Glu.(mM)	25	0	0	0	0
Compound C(μM)	0	0	1.25	2.5	5.0

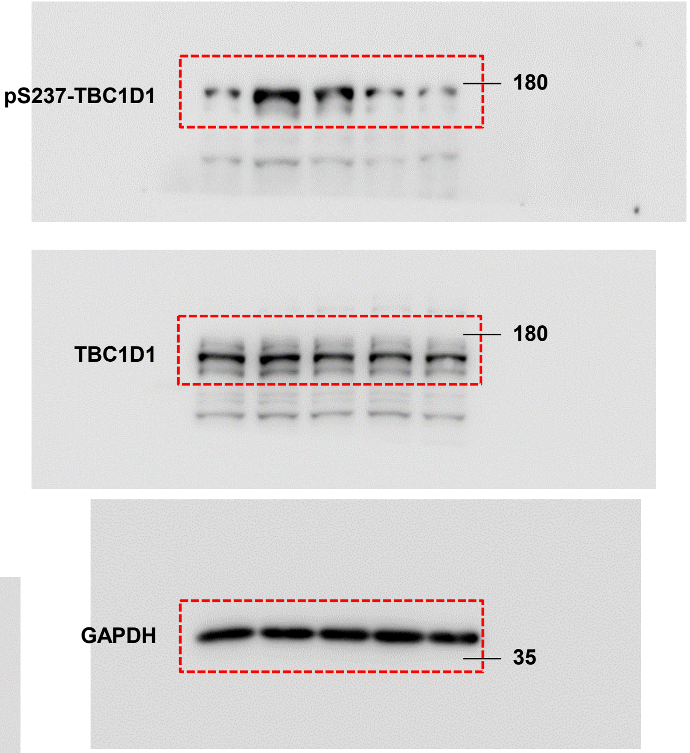

Chemiluminescence Western Blotting,
Autoradiography machine (Tanon-5200) detection

Figure 5I

Glu.(mM)	25	0	0	0	0
Compound C(μM)	0	0	1.25	2.5	5.0

Glu.(mM)	25	0	0	0	0
Compound C(μM)	0	0	1.25	2.5	5.0

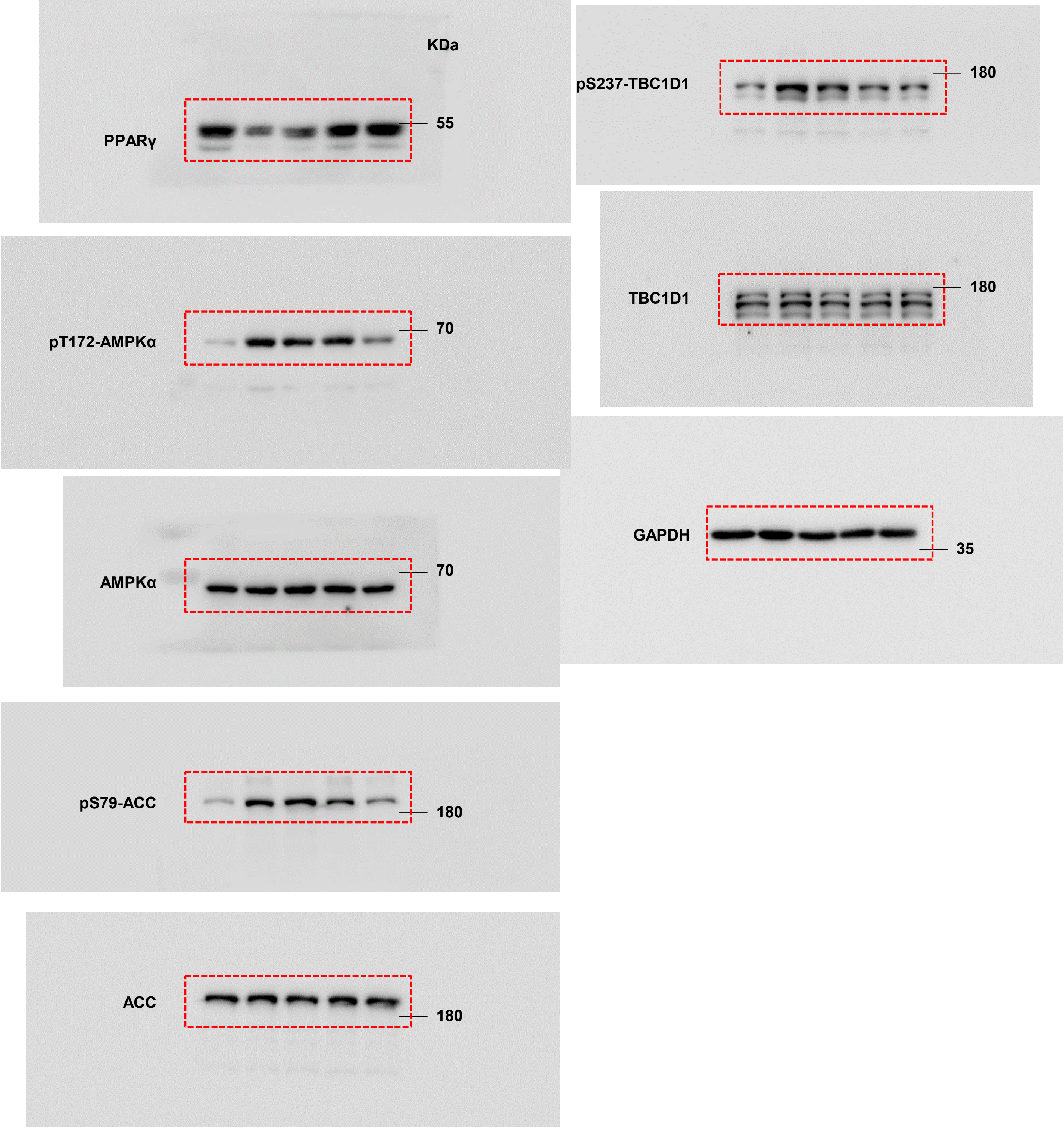

Chemiluminescence Western Blotting,
Autoradiography machine (Tanon-5200) detection

Figure 5J

PPARγ2-MYC	+	+	+
Glu.(mM)	25	0	0
Compound C(μM)	0	0	5.0

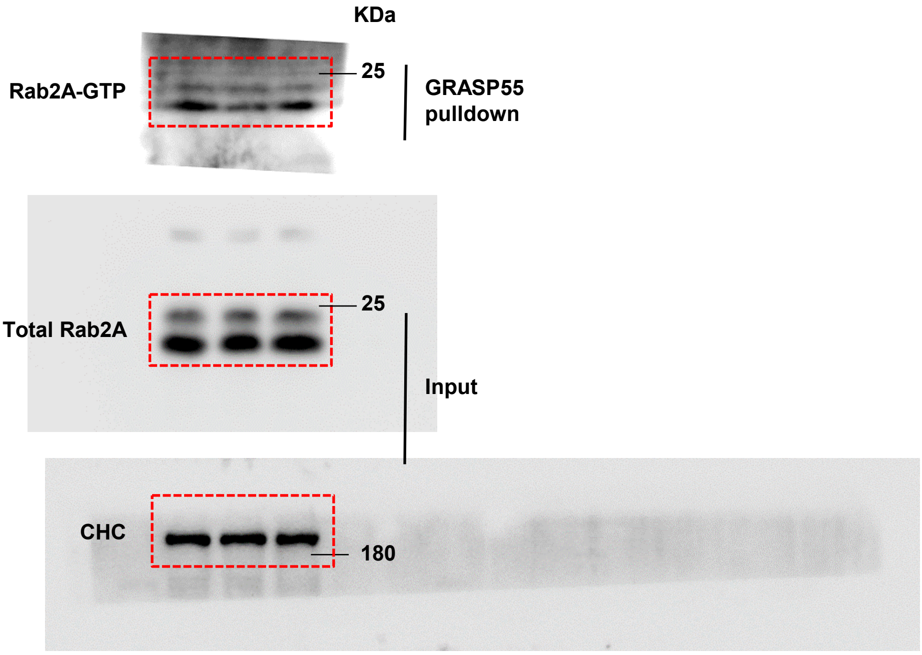

Chemiluminescence Western Blotting,
Autoradiography machine (Tanon-5200) detection

Figure 7A

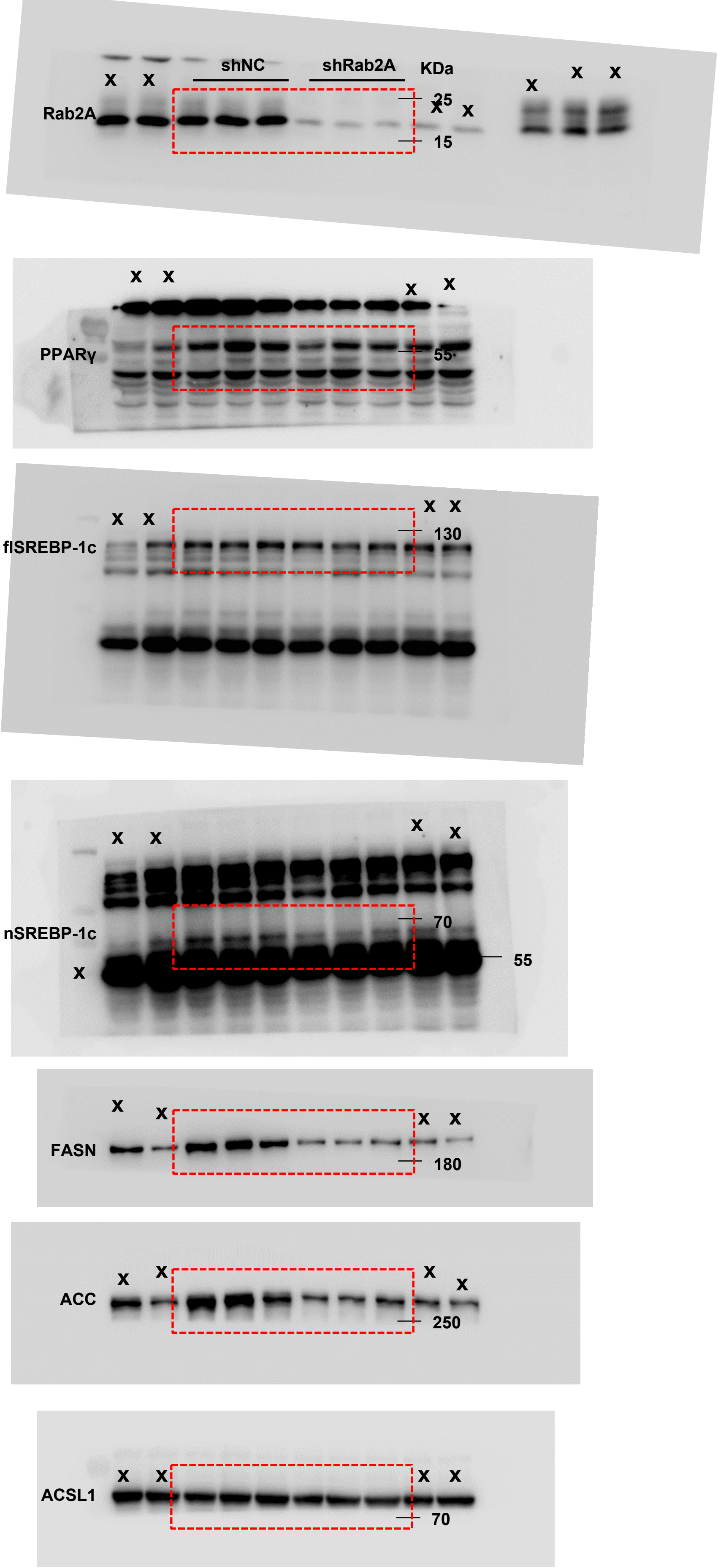

Chemiluminescence Western Blotting, Autoradiography machine (Tanon-5200) detection

Figure 7A

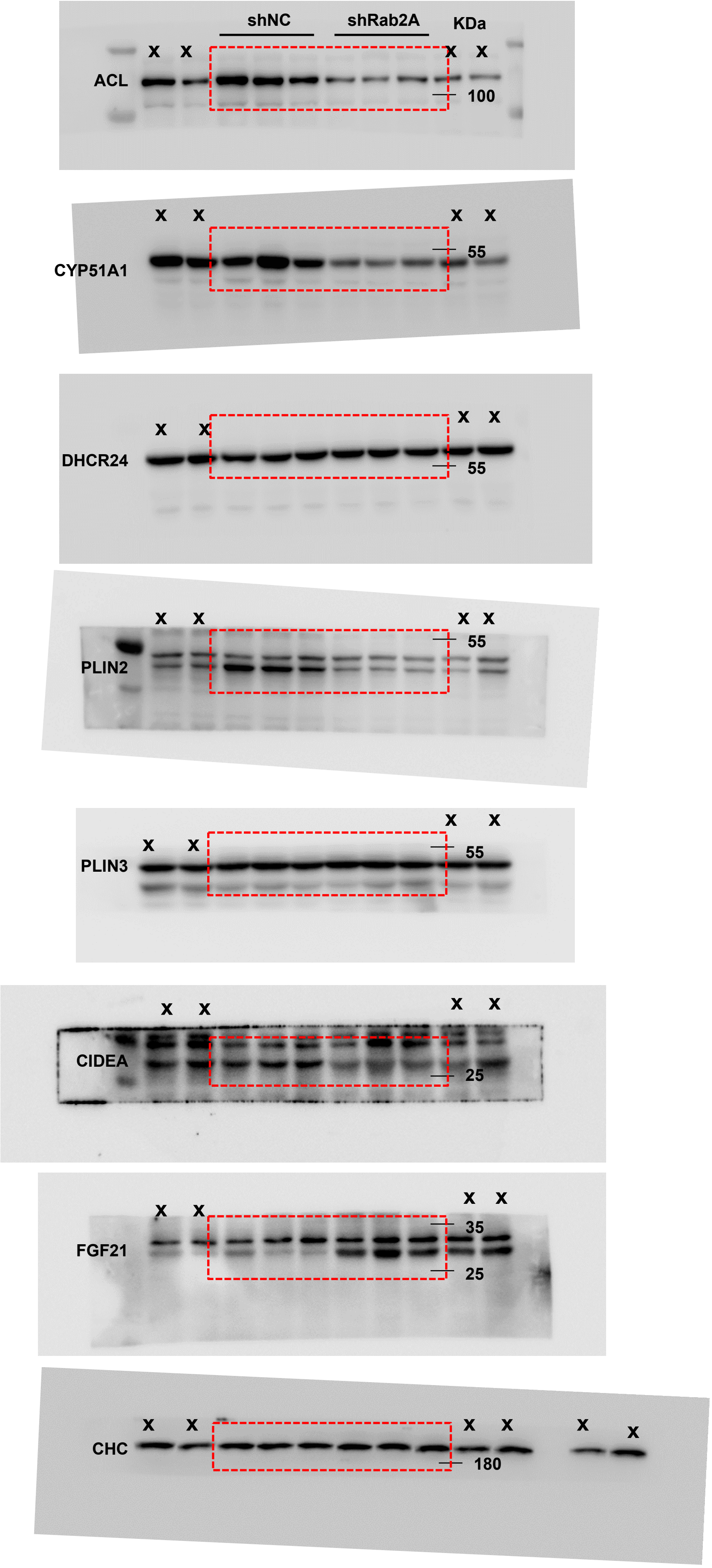

Chemiluminescence Western Blotting,
Autoradiography machine (Tanon-5200) detection

Figure S1I

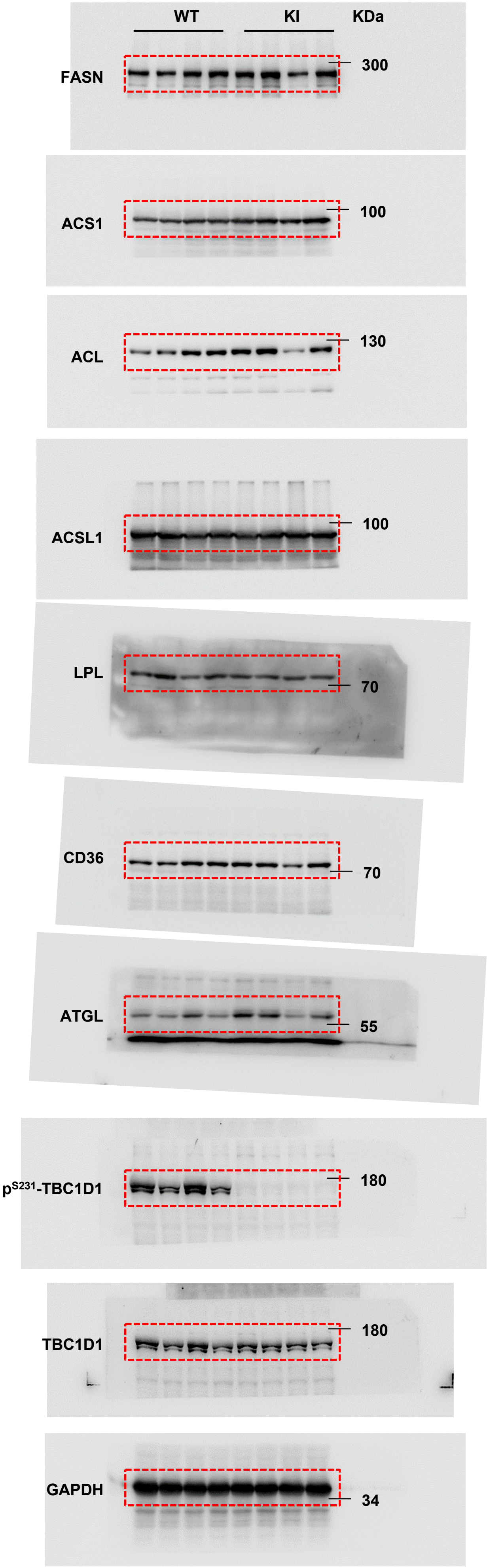

Chemiluminescence Western Blotting,
Autoradiography machine (Tanon-5200) detection

Figure S1K

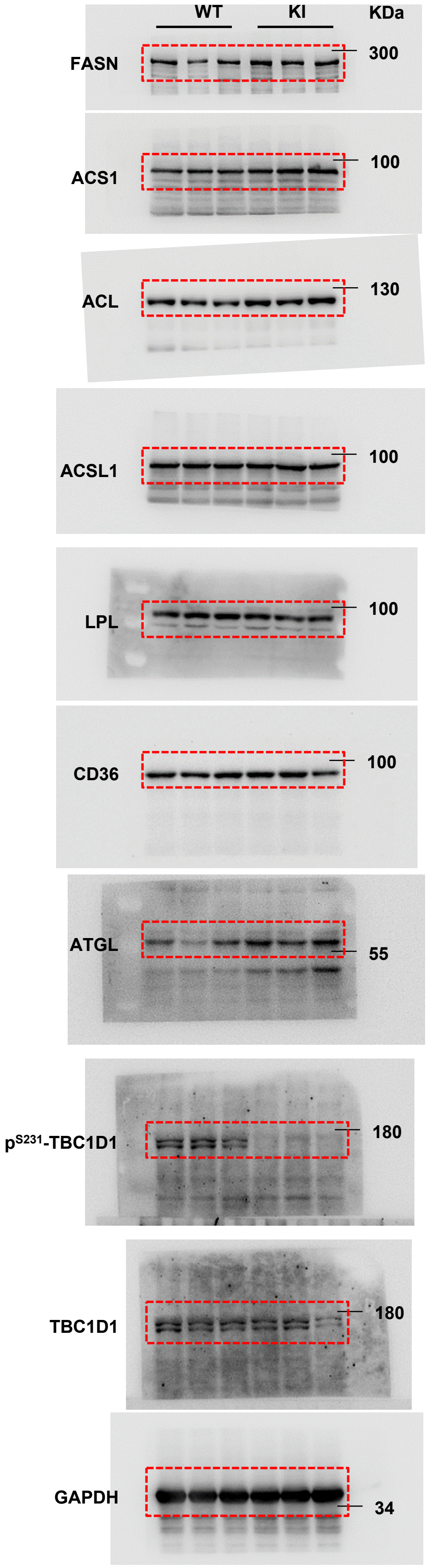

Chemiluminescence Western Blotting,
Autoradiography machine (Tanon-5200) detection

Figure S2C

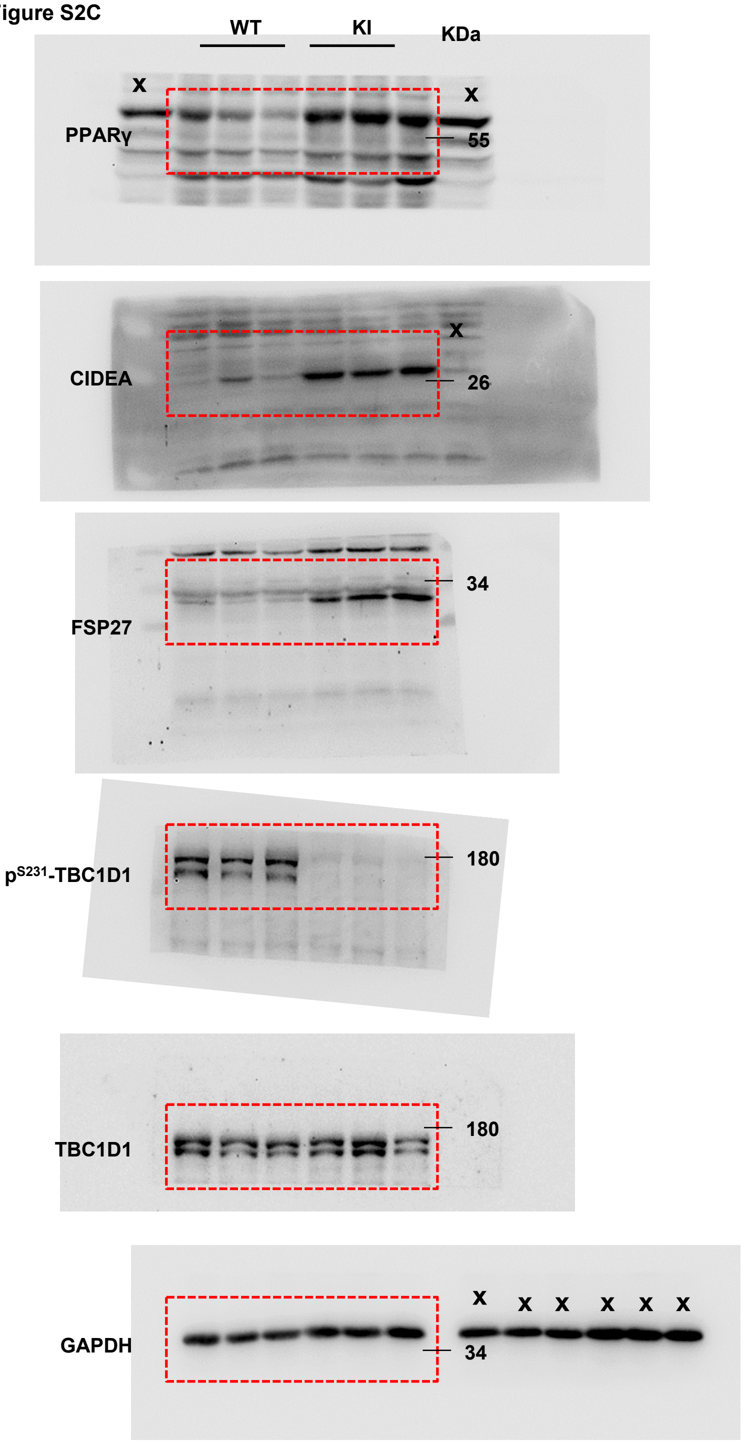

Chemiluminescence Western Blotting, Autoradiography machine (Tanon-5200) detection

Figure S3A

HA-Vector	+	-
HA-TBC1D1	-	+

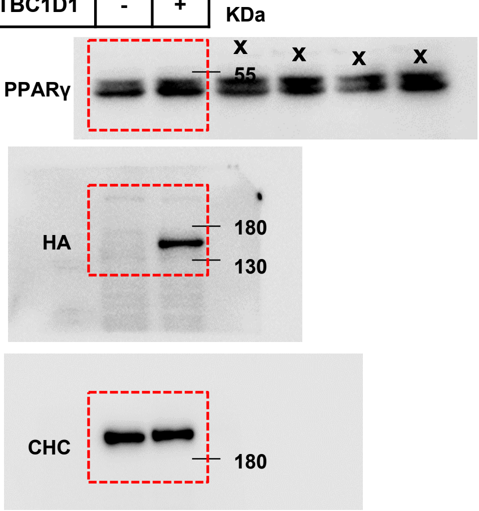

Figure S3B

HA-Vector	+	-
HA-TBC1D1	-	+
PPARγ1-MYC	+	+

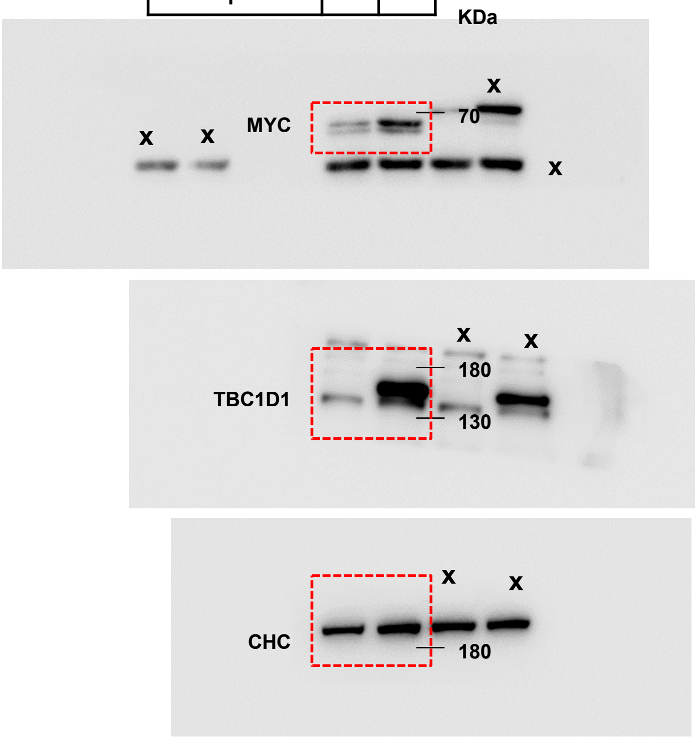

Figure S3C

HA-Vector	+	-
HA-TBC1D1	-	+
PPARγ1-MYC	+	+

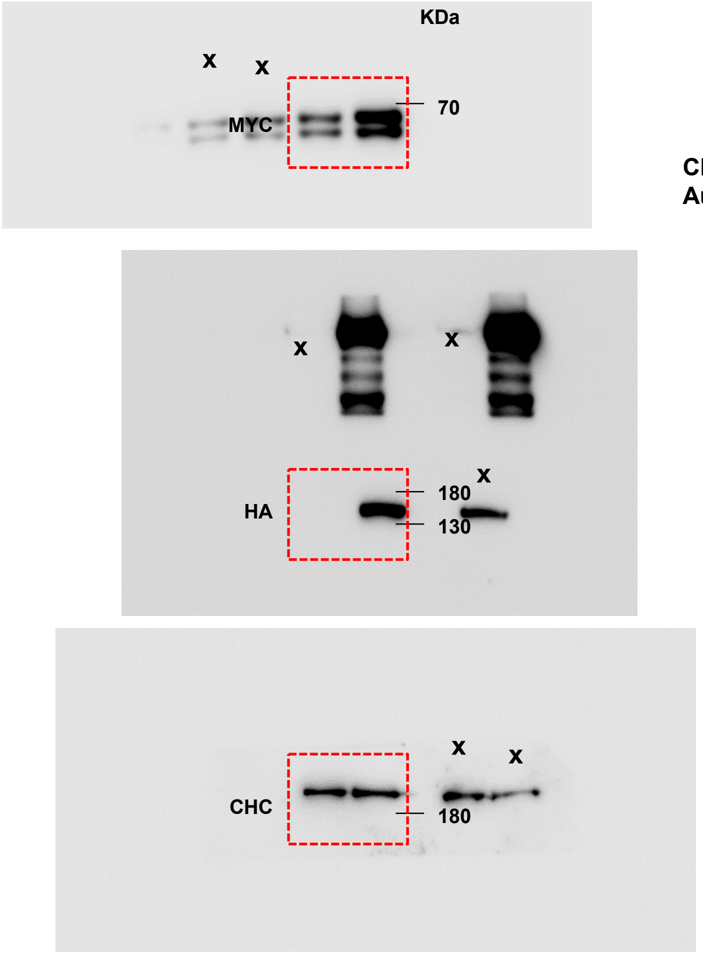

Chemiluminescence Western Blotting,
Autoradiography machine (Tanon-5200) detection

Figure S3D

HA-Vector (µg)	1.5	1.4	1.2	0.5	-
HA-TBC1D1 (µg)	-	0.1	0.3	1.0	1.5
PPARγ1-MYC	+	+	+	+	+

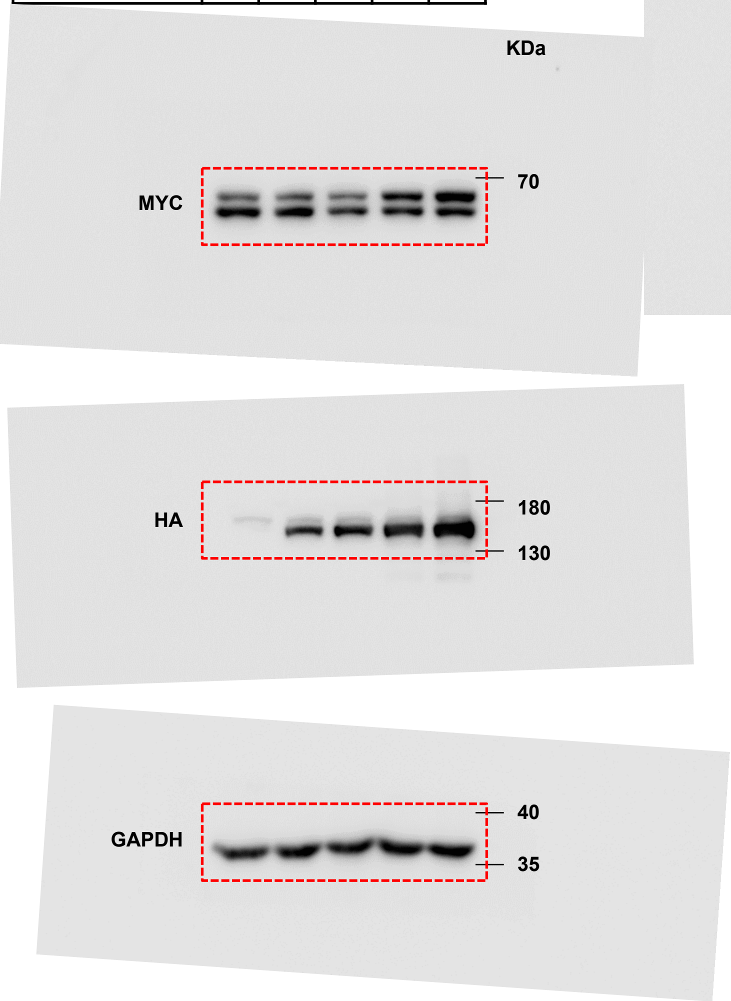

Figure S3E

HA-Vector	+	-
HA-TBC1D1	-	+
PPARγ2-MYC	+	+

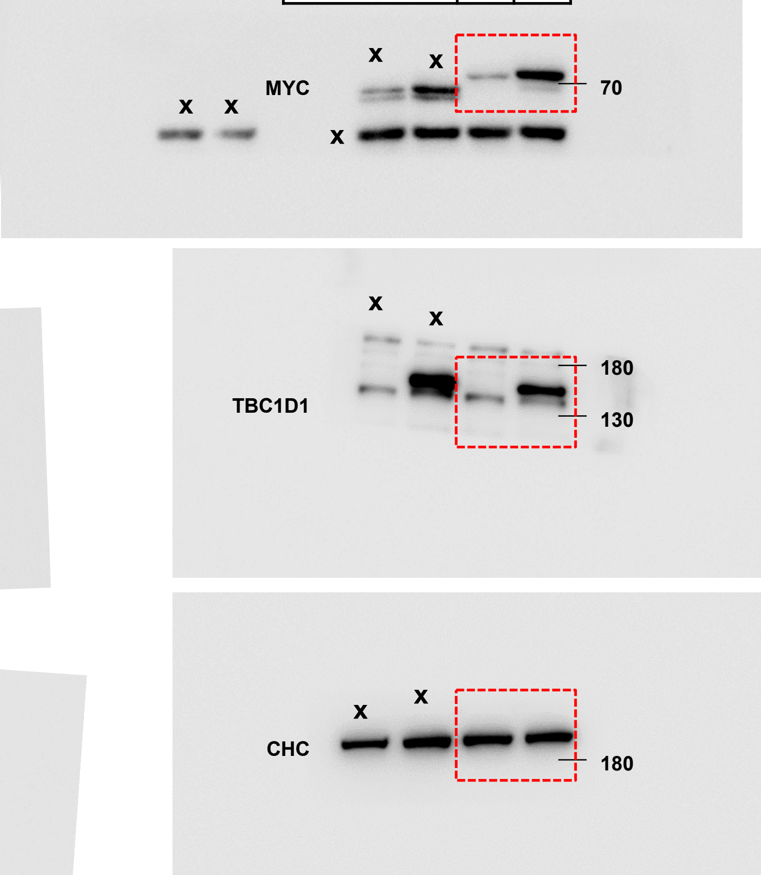

Chemiluminescence Western Blotting,
Autoradiography machine (Tanon-5200) detection

Figure S3F

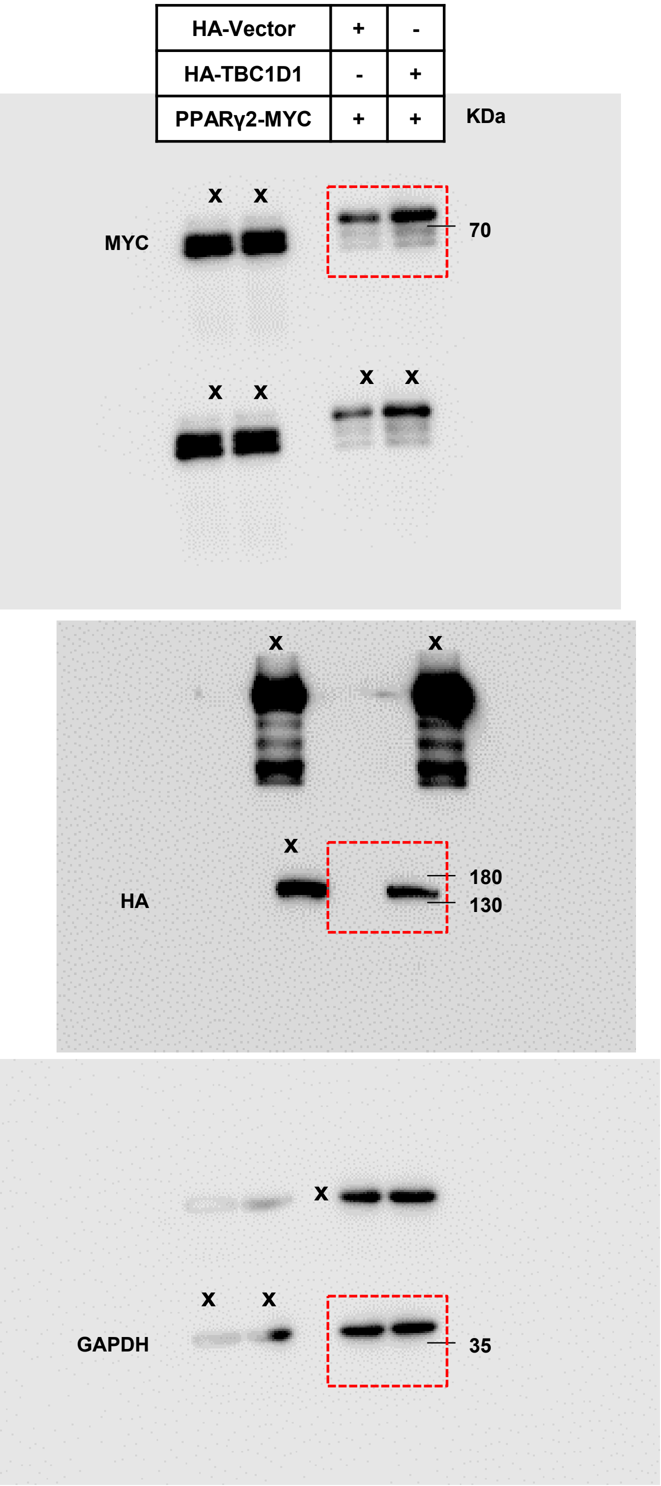

Figure S3G

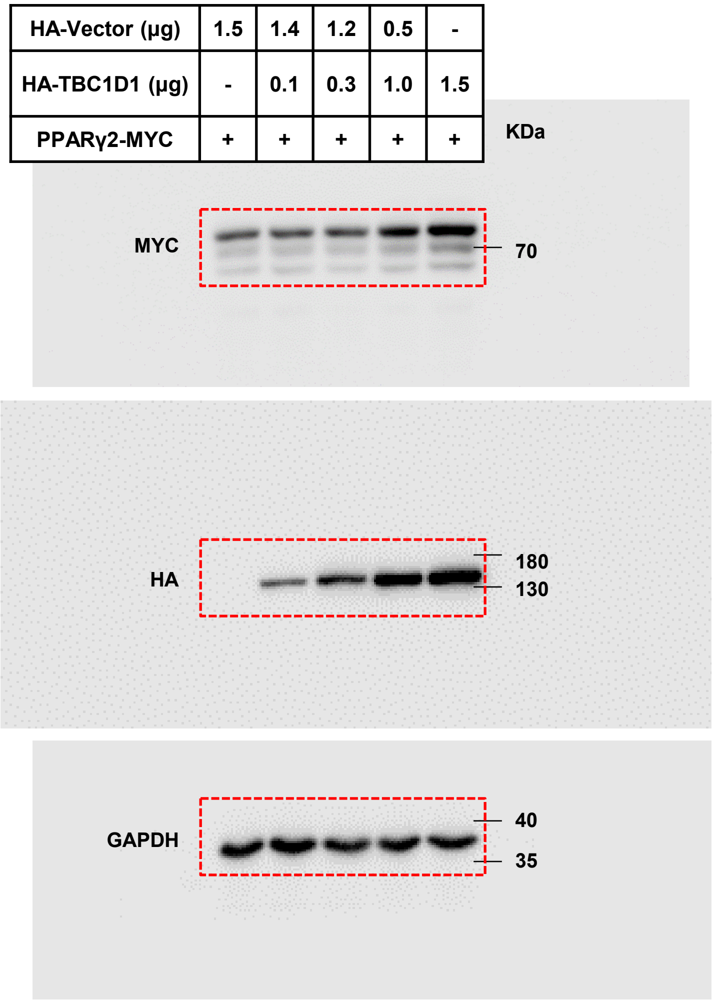

Chemiluminescence Western Blotting,
Autoradiography machine (Tanon-5200) detection

Figure S3H

HA-Vector	+	-	-	-	-	-	-
HA-TBC1D1(μg)	-	0.1	0.3	1.0	-	-	-
HA-TBC1D1^{S237A}(μg)	-	-	-	-	0.1	0.3	1.0
PPARγ2-MYC	+	+	+	+	+	+	+

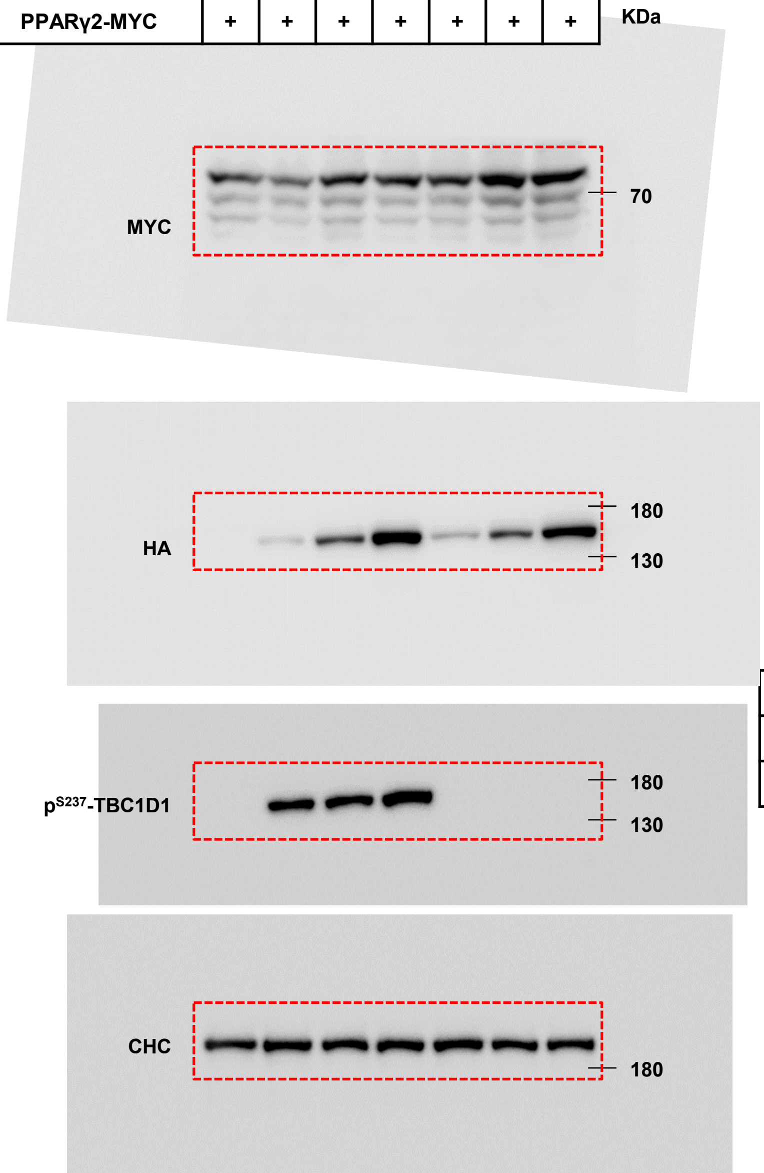

Figure S3I

Flag-Vector	+	-	-
Flag-TBC1D1^{WT}	-	+	-
Flag-TBC1D1^{S237A}	-	-	+

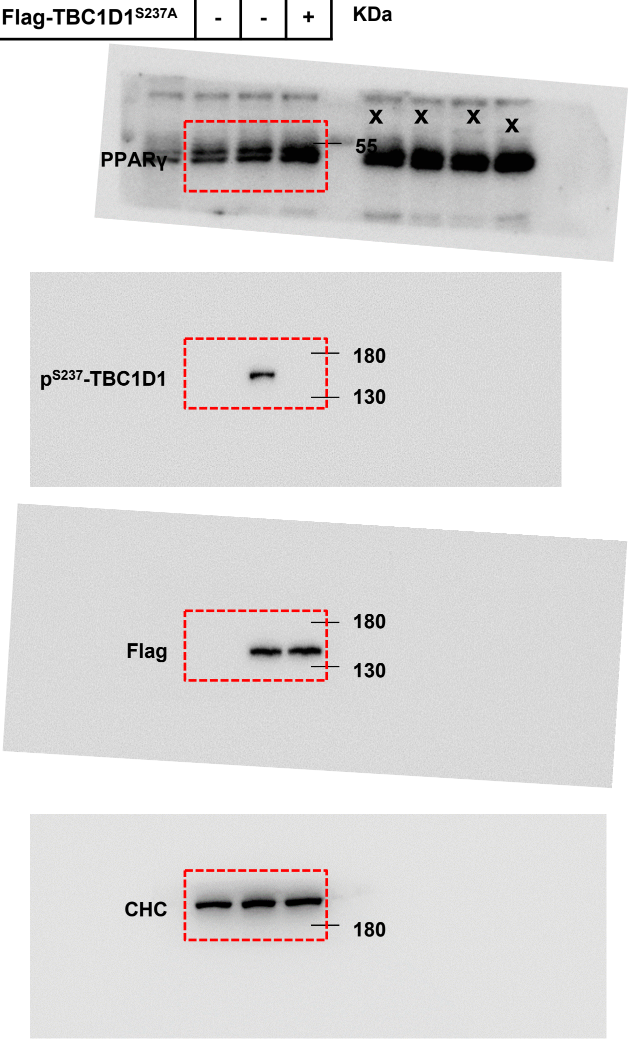

Chemiluminescence Western Blotting,
Autoradiography machine (Tanon-5200) detection

Figure S3K

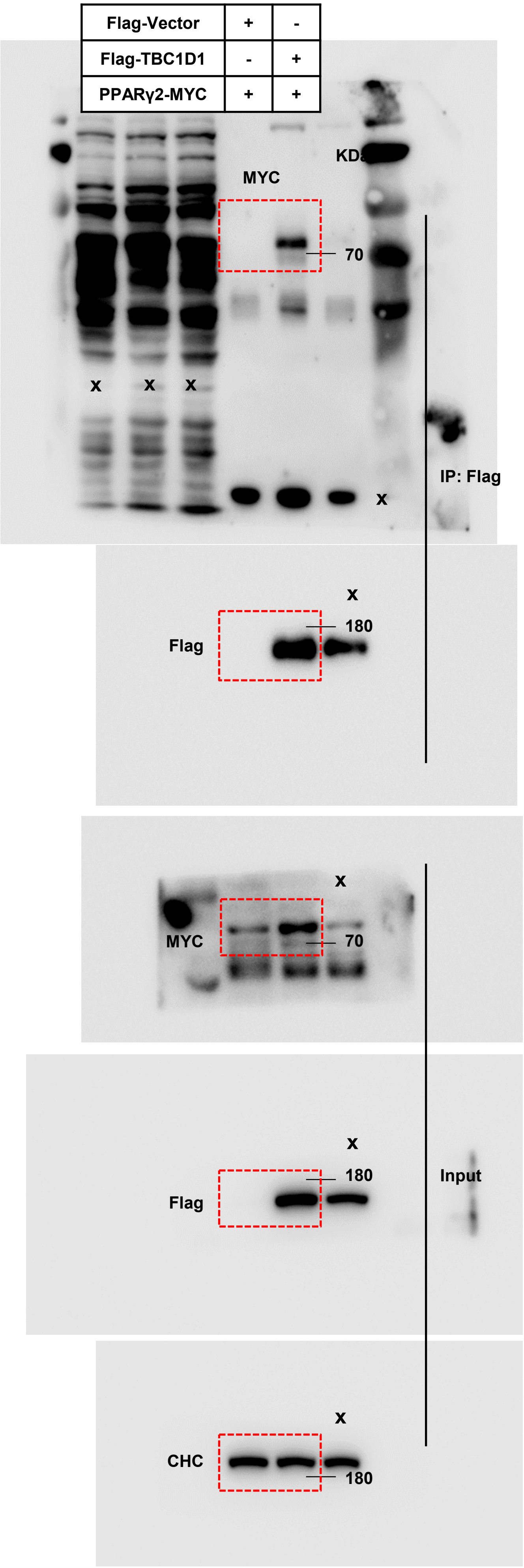

Figure S3L

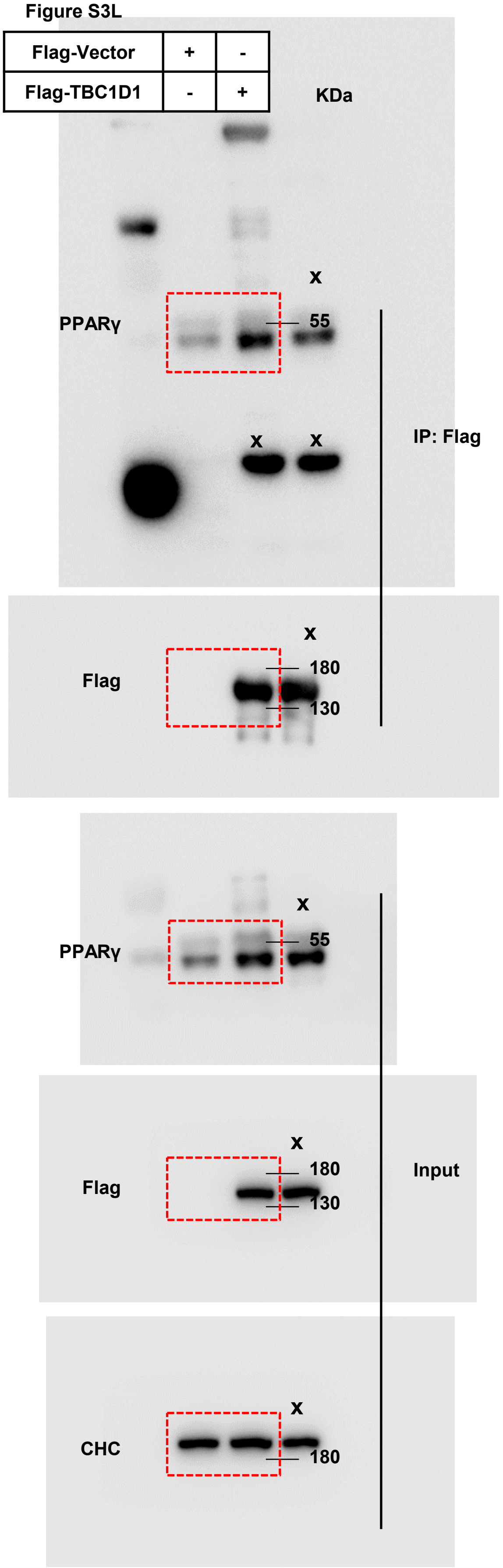

Chemiluminescence Western Blotting,
Autoradiography machine (Tanon-5200) detection

Figure S3M

HA-Vector	+						-					
HA-TBC1D1	-						+					
PPARγ2-MYC	+											
CHX (Hrs)	-	1	2	4	6	8	-	1	2	4	6	8

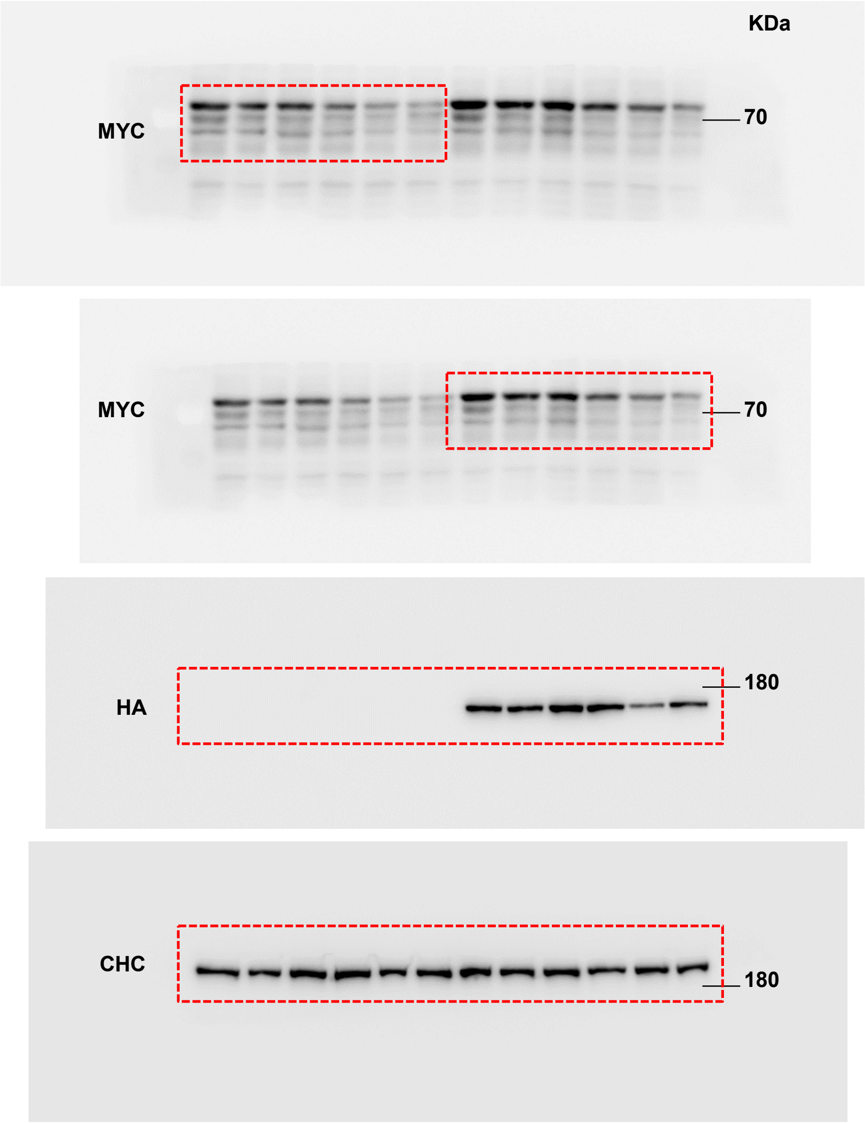

Chemiluminescence Western Blotting,
Autoradiography machine (Tanon-5200) detection

Figure S4A

FLAG-Vector	+	-	-	-	-
FLAG-Rab2A(μg)	-	0.1	0.3	1.0	1.5
PPARγ2-MYC	+	+	+	+	+

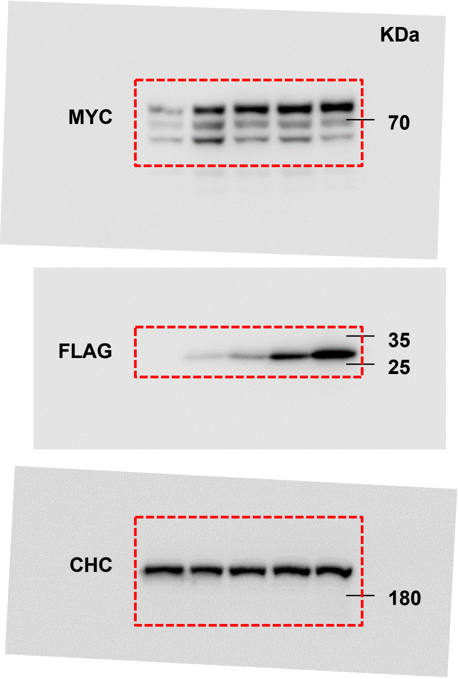

Figure S4B

FLAG-Vector	+	-	-	-	-
FLAG-Rab2B(μg)	-	0.1	0.3	1.0	1.5
PPARγ2-MYC	+	+	+	+	+

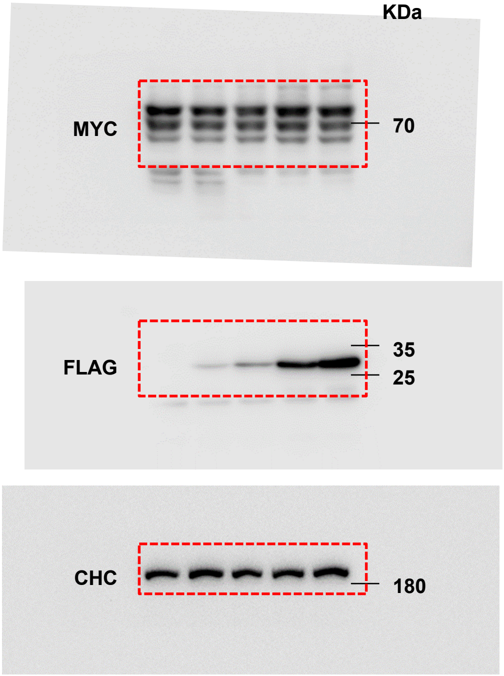

Figure S4C

FLAG-Vector	+	-	-	-	-
FLAG-Rab8A(μg)	-	0.1	0.3	1.0	1.5
PPARγ2-MYC	+	+	+	+	+

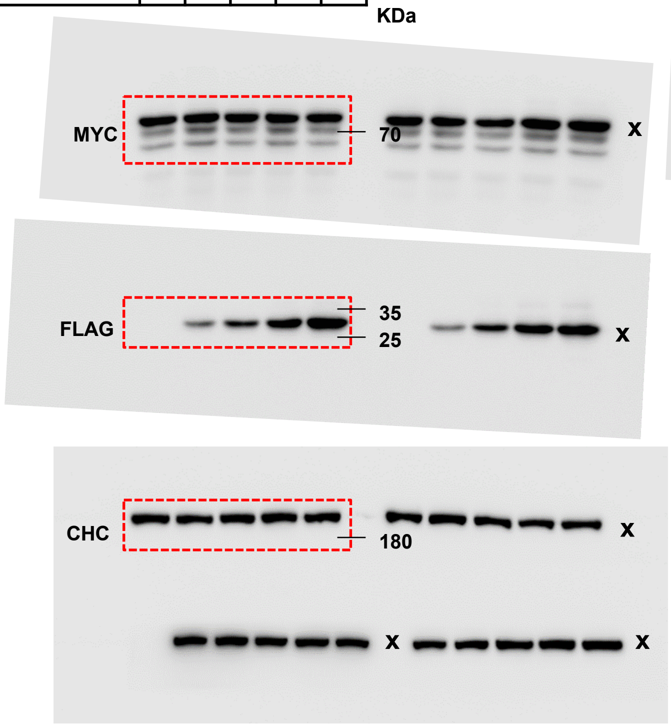

Figure S4D

FLAG-Vector	+	-	-	-	-
FLAG-Rab8B(μg)	-	0.1	0.3	1.0	1.5
PPARγ2-MYC	+	+	+	+	+

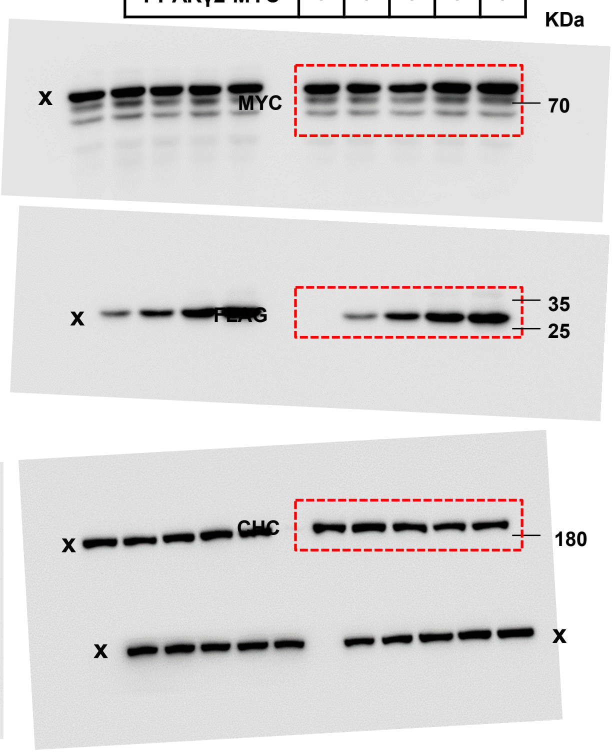

Chemiluminescence Western Blotting,
Autoradiography machine (Tanon-5200) detection

Figure S4E

FLAG-Vector	+	-	-	-	-
FLAG-Rab10(μg)	-	0.1	0.3	1.0	1.5
PPARγ2-MYC	+	+	+	+	+

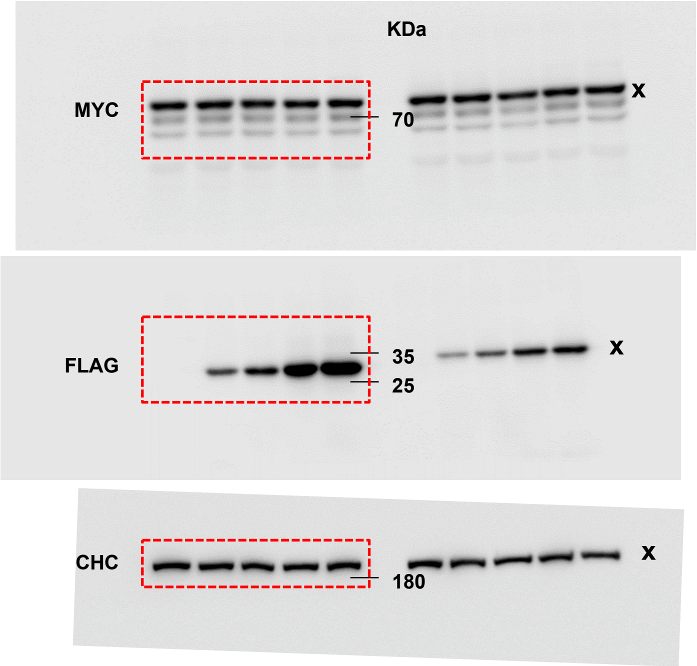

Figure S4F

Chemiluminescence Western Blotting, Autoradiography machine (Tanon-5200) detection

Flag-Vector	+	-	-	-	-
Flag-Rab14(μg)	-	0.1	0.3	1.0	1.5
PPARγ2-MYC	+	+	+	+	+

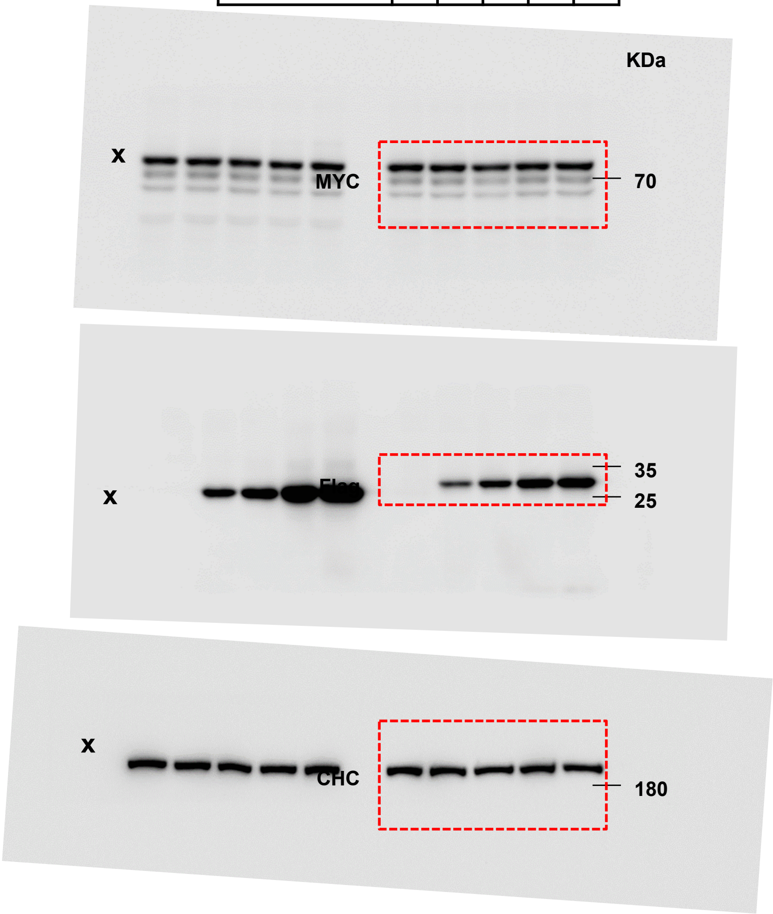

Figure S4G

Flag-Vector	+	-	-	-	-
Flag-Rab1A(μg)	-	0.1	0.3	1.0	1.5
PPARγ2-MYC	+	+	+	+	+

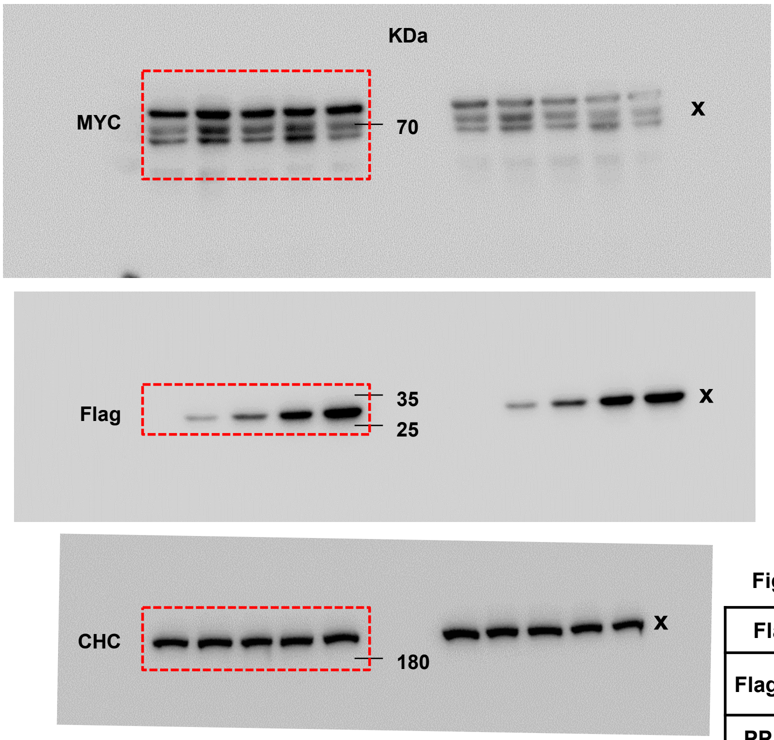

Chemiluminescence Western Blotting, Autoradiography machine (Tanon-5200) detection

Figure S4H

Flag-Vector	+	-	-	-	-
Flag-Rab24(μg)	-	0.1	0.3	1.0	1.5
PPARγ2-MYC	+	+	+	+	+

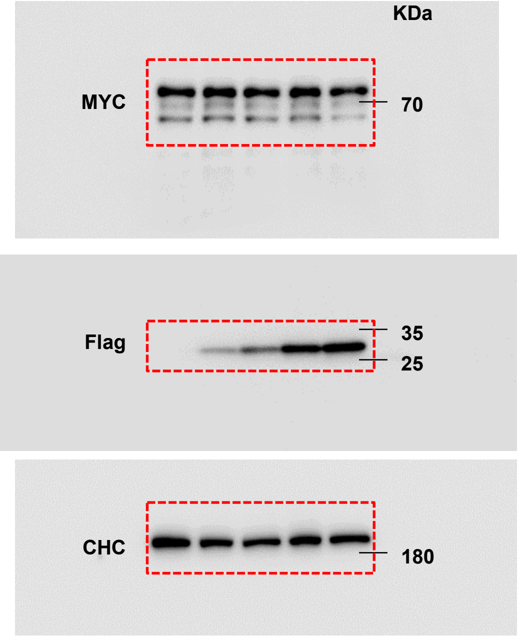

Figure S4I

Flag-Vector	+	-	-	-	-
Flag-Rab35(μg)	-	0.1	0.3	1.0	1.5
PPARγ2-MYC	+	+	+	+	+

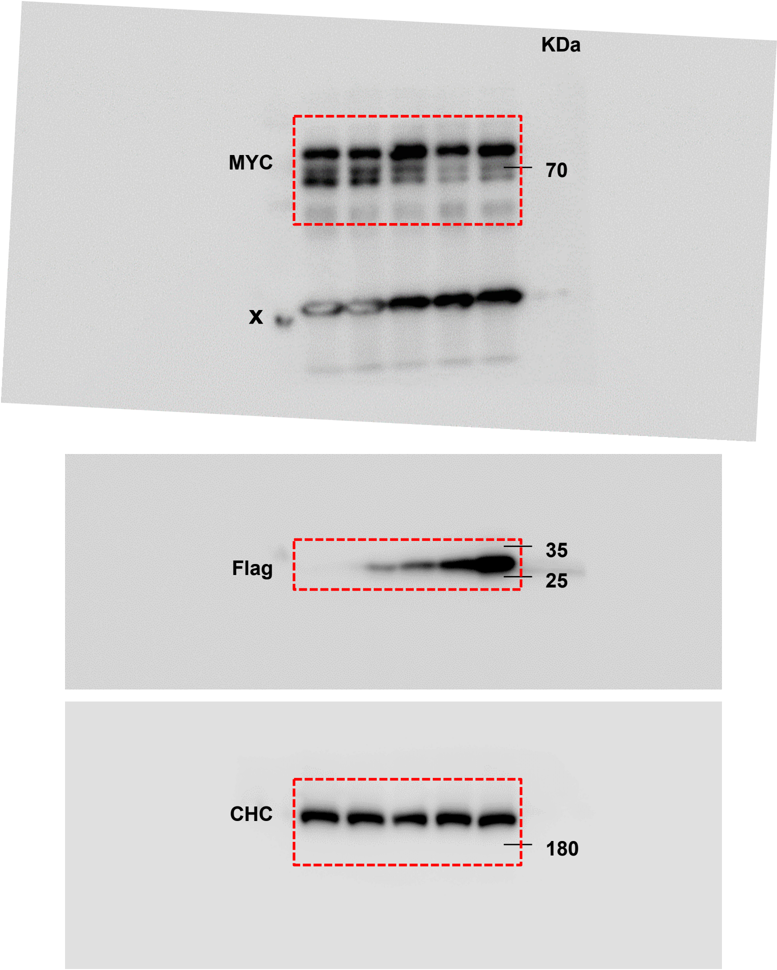

Figure S4J

Flag-Vector	+	-	-	-	-
Flag-Rab7A(μg)	-	0.1	0.3	1.0	1.5
PPARγ2-MYC	+	+	+	+	+

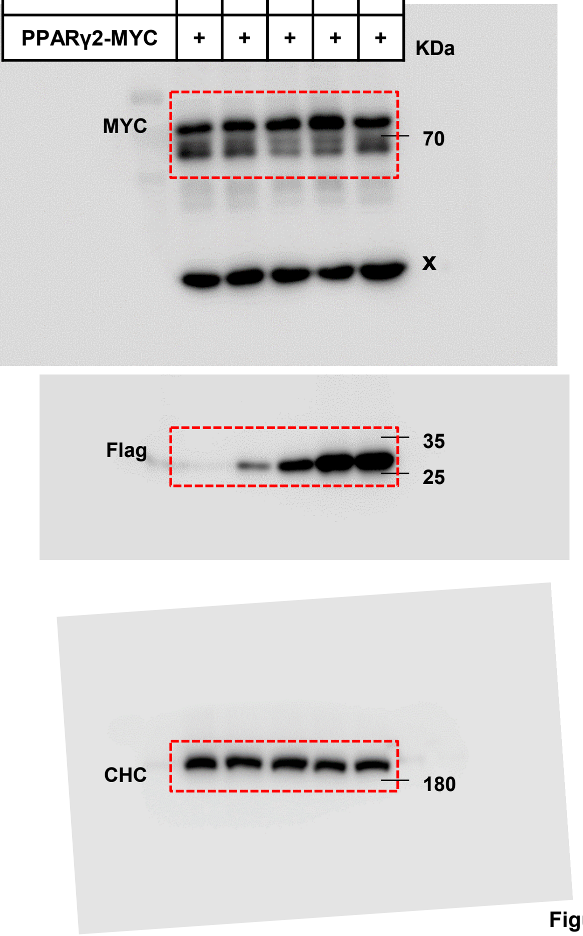

Figure S4K

Flag-Vector	+	-	-	-	-
Flag-Rab15(μg)	-	0.1	0.3	1.0	1.5
PPARγ2-MYC	+	+	+	+	+

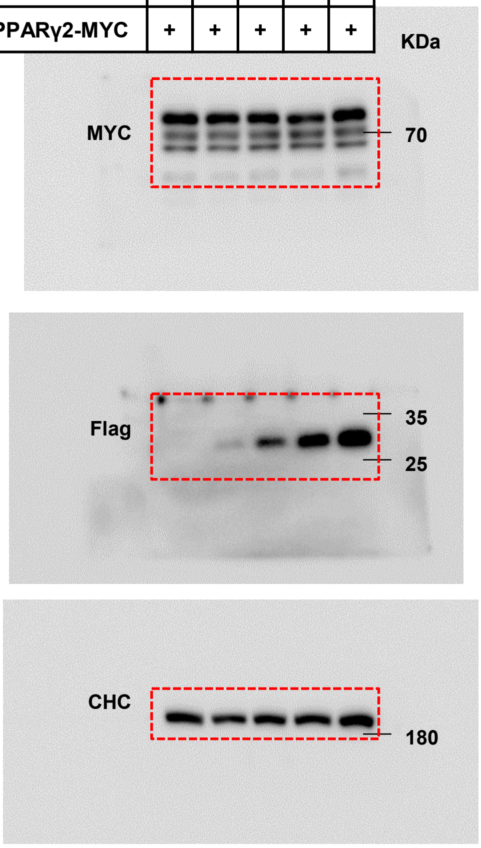

Figure S4L

Flag-Vector	+	-	-	-	-
Flag-Rab40A(μg)	-	0.1	0.3	1.0	1.5
PPARγ2-MYC	+	+	+	+	+

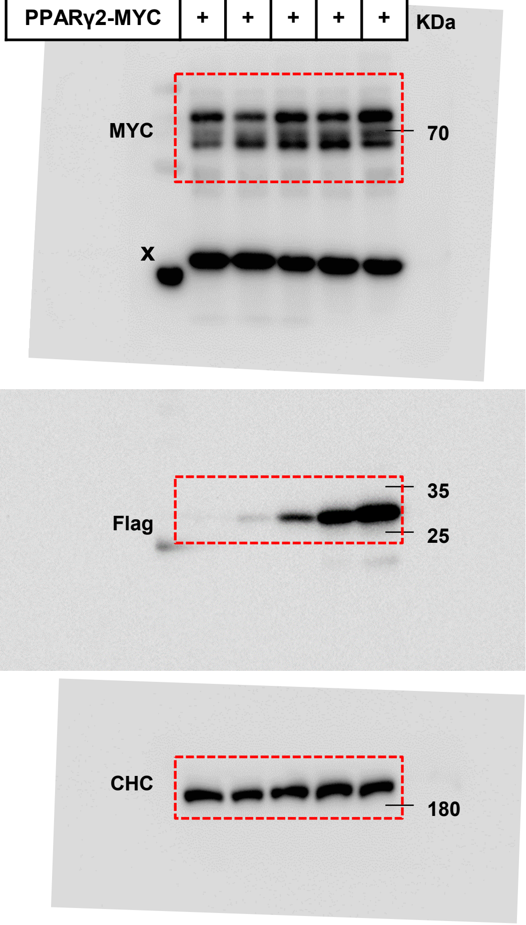

Chemiluminescence Western Blotting, Autoradiography machine (Tanon-5200) detection

Figure S5A

Flag-Vector	+	-	-	-	-
Flag-Rab1B(μg)	-	0.1	0.3	1.0	1.5
PPARγ2-MYC	+	+	+	+	+

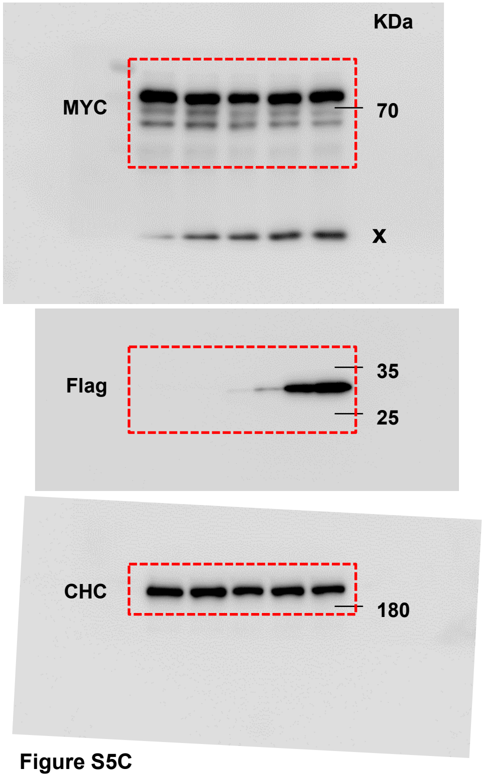

Figure S5B

Flag-Vector	+	-	-	-	-
Flag-Rab5A(μg)	-	0.1	0.3	1.0	1.5
PPARγ2-MYC	+	+	+	+	+

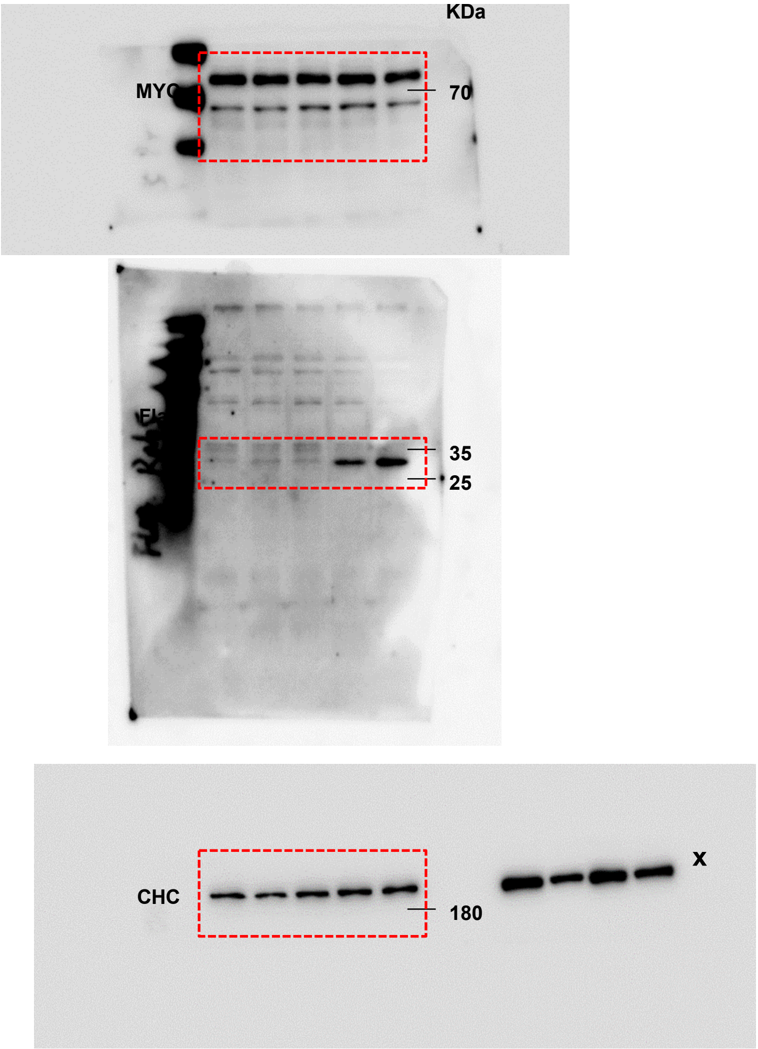

Figure S5C

Flag-Vector	+	-	-	-	-
Flag-Rab9A(μg)	-	0.1	0.3	1.0	1.5
PPARγ2-MYC	+	+	+	+	+

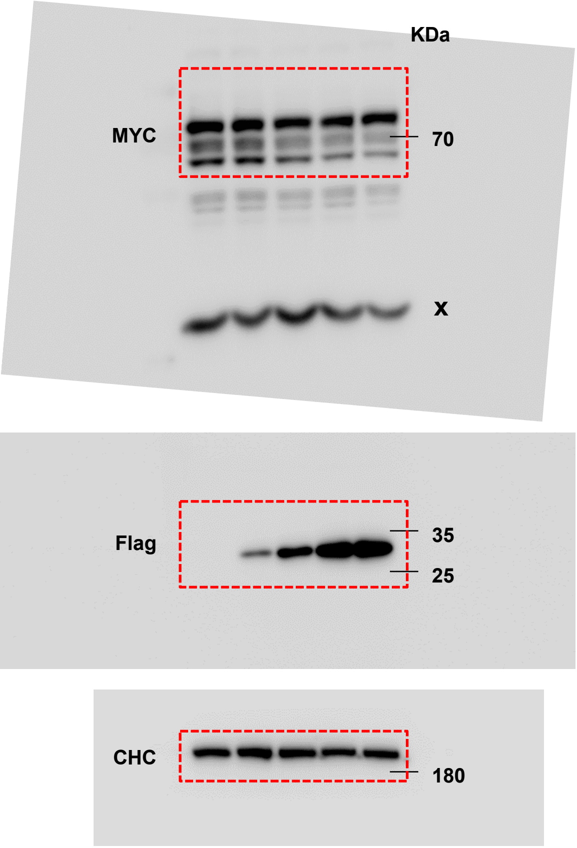

Chemiluminescence Western Blotting, Autoradiography machine (Tanon-5200) detection

Figure S5A

Flag-Vector	+	-	-	-	-
Flag-Rab1B(μg)	-	0.1	0.3	1.0	1.5
PPARγ2-MYC	+	+	+	+	+

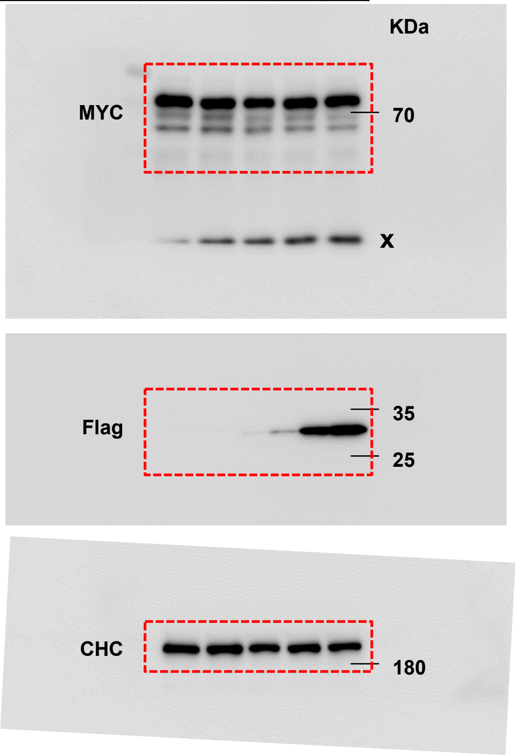

Figure S5B

Flag-Vector	+	-	-	-	-
Flag-Rab5A(μg)	-	0.1	0.3	1.0	1.5
PPARγ2-MYC	+	+	+	+	+

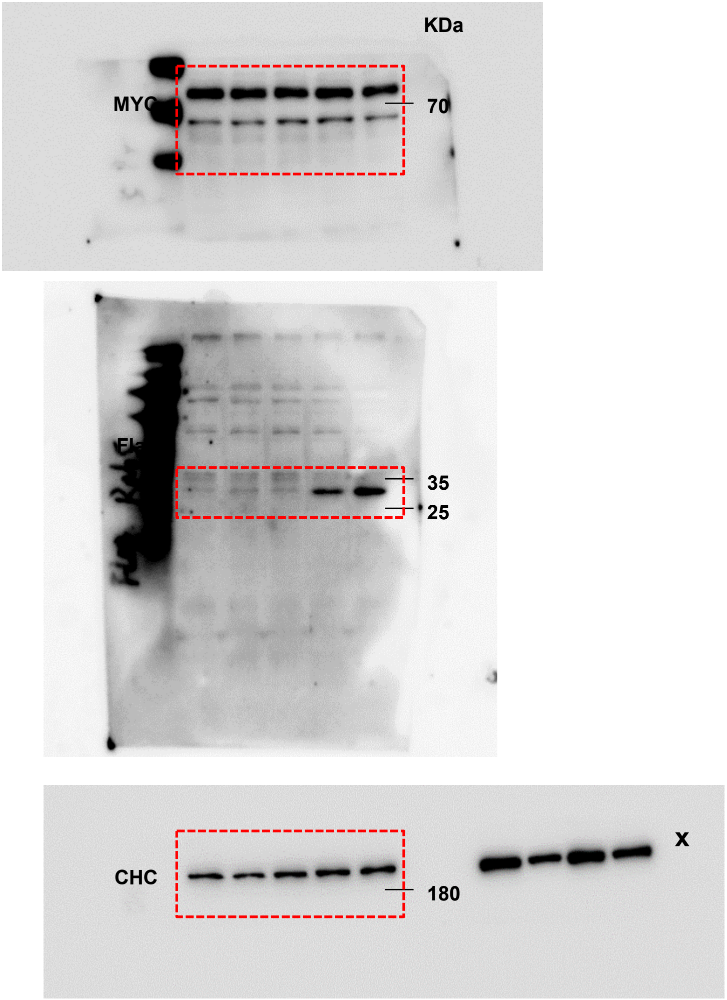

Chemiluminescence Western Blotting,
Autoradiography machine (Tanon-5200) detection

Figure S5C

Flag-Vector	+	-	-	-	-
Flag-Rab9A(μg)	-	0.1	0.3	1.0	1.5
PPARγ2-MYC	+	+	+	+	+

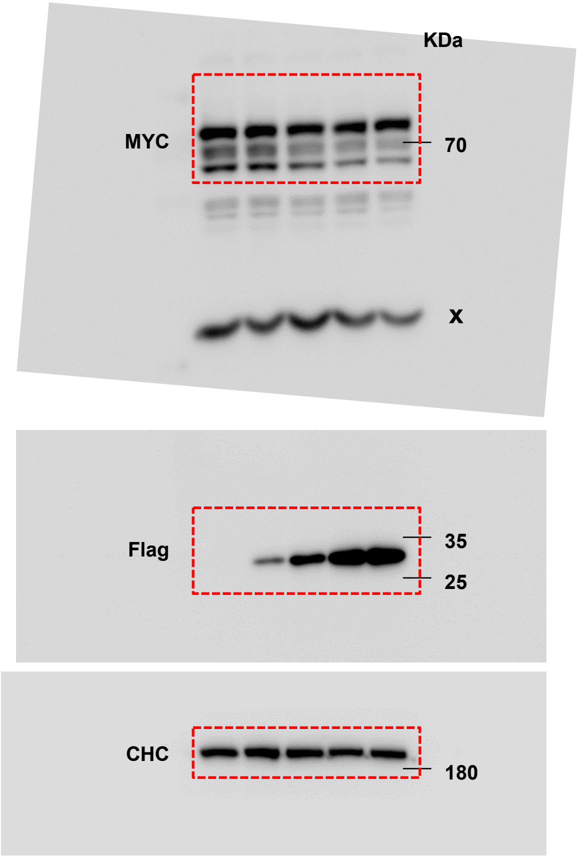

Figure S5D

Flag-Vector	+	-	-	-	-
Flag-Rab9B(μg)	-	0.1	0.3	1.0	1.5
PPARγ2-MYC	+	+	+	+	+

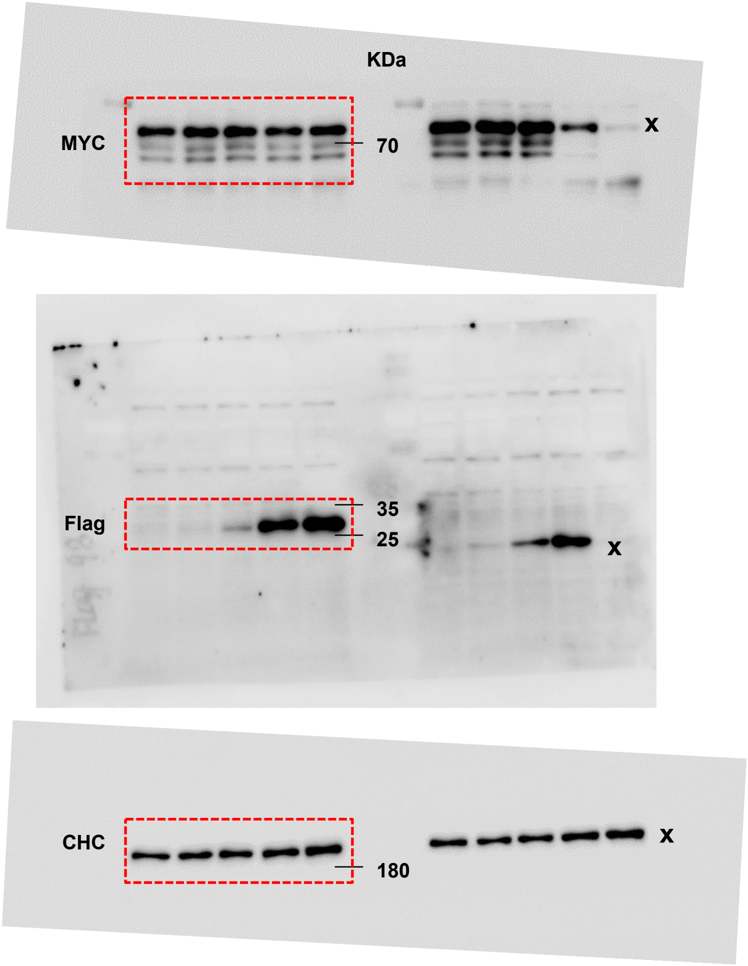

Chemiluminescence Western Blotting, Autoradiography machine (Tanon-5200) detection

Figure S5E

Flag-Vector	+	-	-	-	-
Flag-Rab11B(μg)	-	0.1	0.3	1.0	1.5
PPARγ2-MYC	+	+	+	+	+

KDa

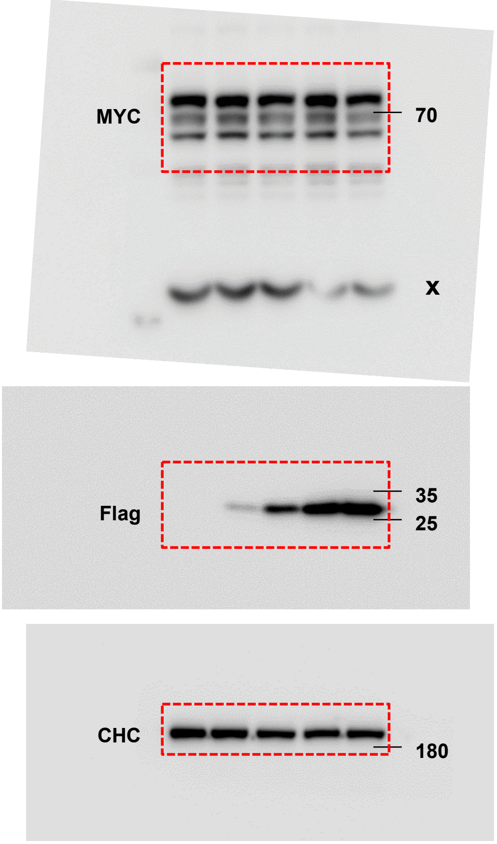

Figure S5F

Flag-Vector	+	-	-	-	-
Flag-Rab22B(μg)	-	0.1	0.3	1.0	1.5
PPARγ2-MYC	+	+	+	+	+

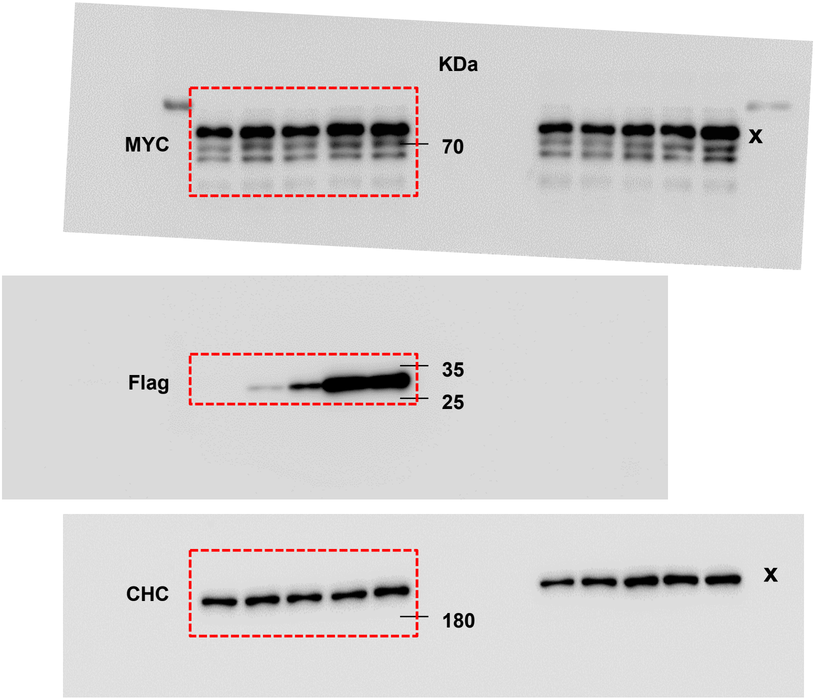

Chemiluminescence Western Blotting,
Autoradiography machine (Tanon-5200) detection

Figure S5G

Flag-Vector	+	-	-	-	-
Flag-Rab32(μg)	-	0.1	0.3	1.0	1.5
PPARγ2-MYC	+	+	+	+	+

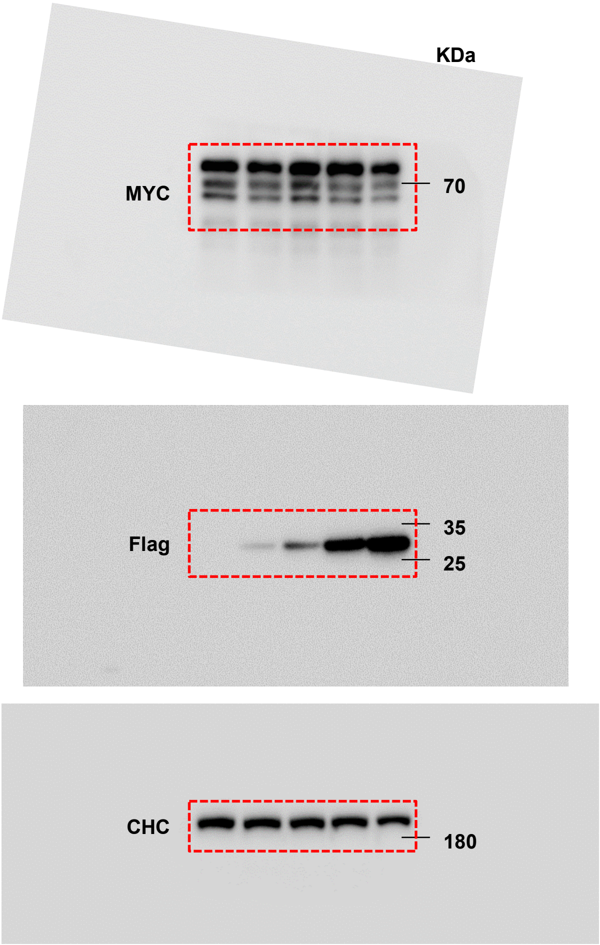

Figure S5H

Flag-Vector	+	-	-	-	-
Flag-Rab4B(μg)	-	0.1	0.3	1.0	1.5
PPARγ2-MYC	+	+	+	+	+

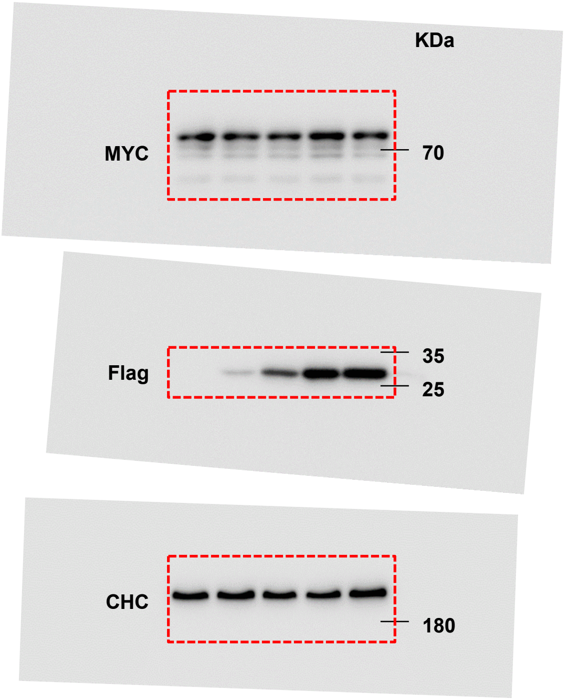

Figure S5I

Flag-Vector	+	-	-	-	-
Flag-Rab39A(μg)	-	0.1	0.3	1.0	1.5
PPARγ2-MYC	+	+	+	+	+

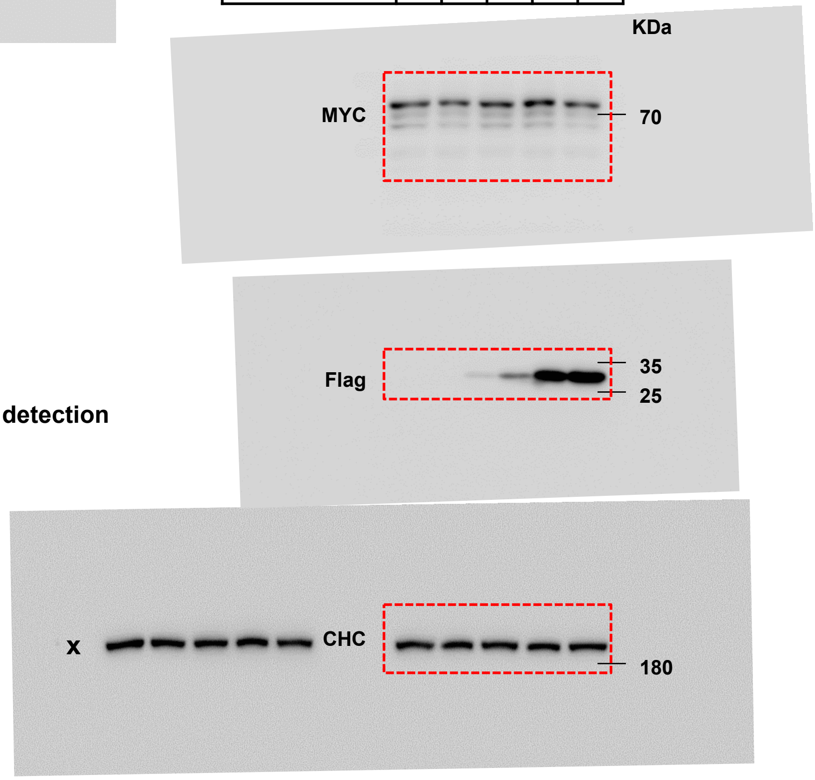

Chemiluminescence Western Blotting, Autoradiography machine (Tanon-5200) detection

Figure S5J

Flag-Vector	+	-	-	-	-
Flag-Rab13(μg)	-	0.1	0.3	1.0	1.5
PPARγ2-MYC	+	+	+	+	+

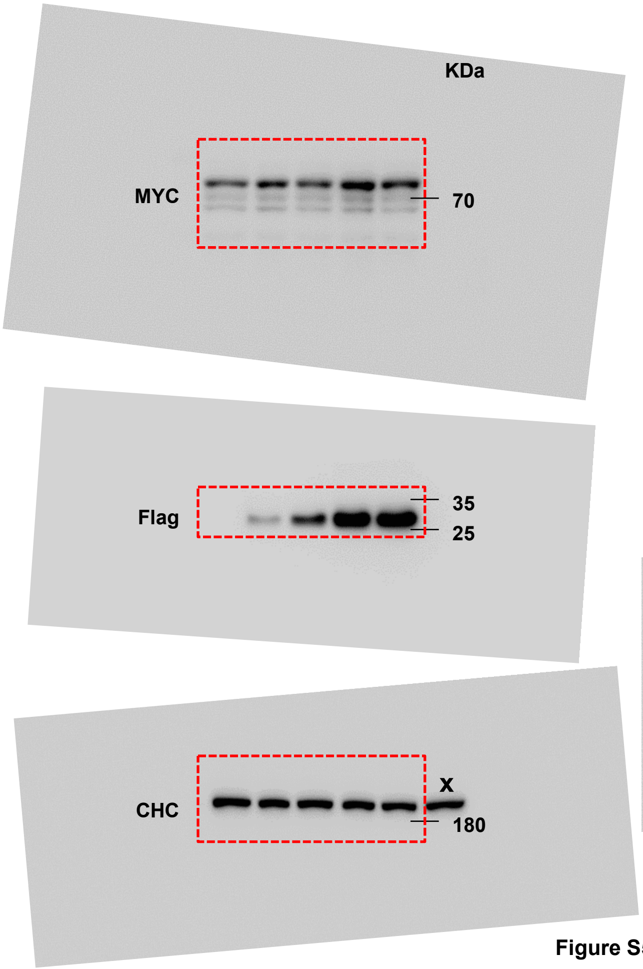

Figure S5K

Flag-Vector	+	-	-	-	-
Flag-Rab23(μg)	-	0.1	0.3	1.0	1.5
PPARγ2-MYC	+	+	+	+	+

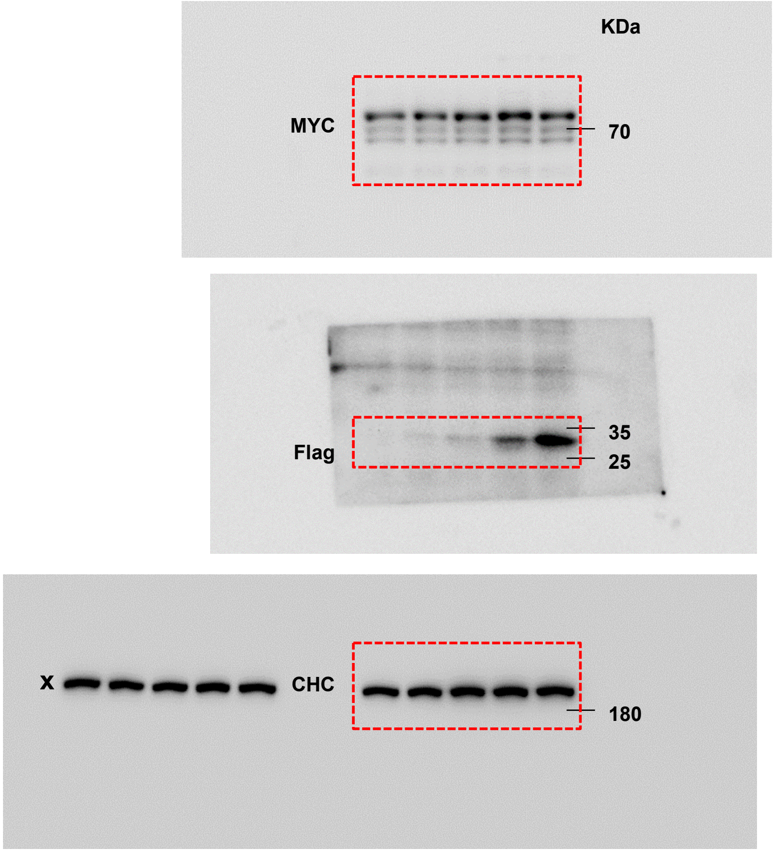

Figure S5L

	Cyto.		Nucl.	
Flag-Vector	+	-	+	-
Flag-Rab2A	-	+	-	+
PPARγ2-MYC	+	+	+	+

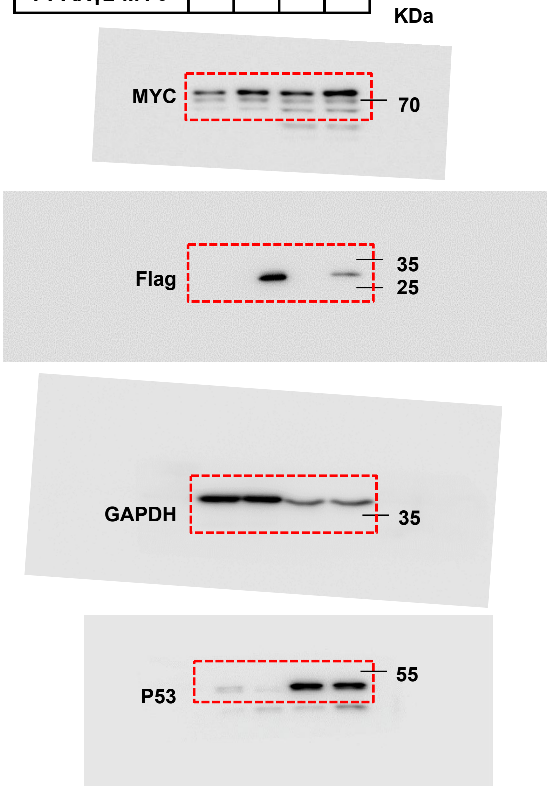

Chemiluminescence Western Blotting,
Autoradiography machine (Tanon-5200) detection

Figure S6A

Flag-Vector	+	-	-	-
Flag-Rab2A^{WT}	-	+	-	-
Flag-Rab2A^{Q65L}	-	-	+	-
Flag-Rab2A^{S20N}	-	-	-	+

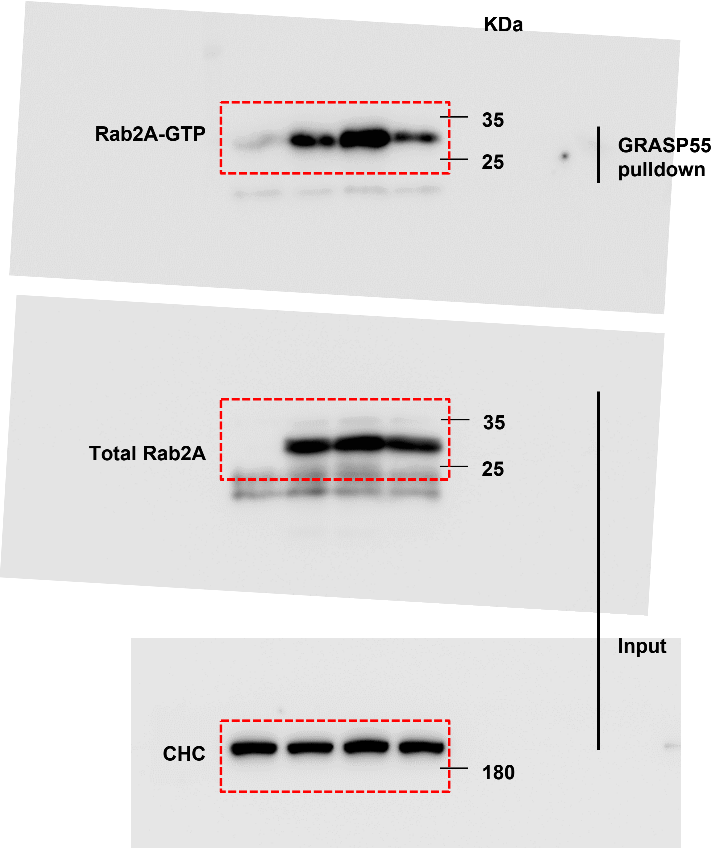

Chemiluminescence Western Blotting,
Autoradiography machine (Tanon-5200) detection

Figure S6B

Flag-Vector	+	+	+	+	-	-	-	-
Flag-Rab2A	-	-	-	-	+	+	+	+
HA-TBC1D1^{WT}(μg)	-	0.1	0.3	1.0	-	0.1	0.3	1.0
PPARγ2-MYC	+	+	+	+	+	+	+	+

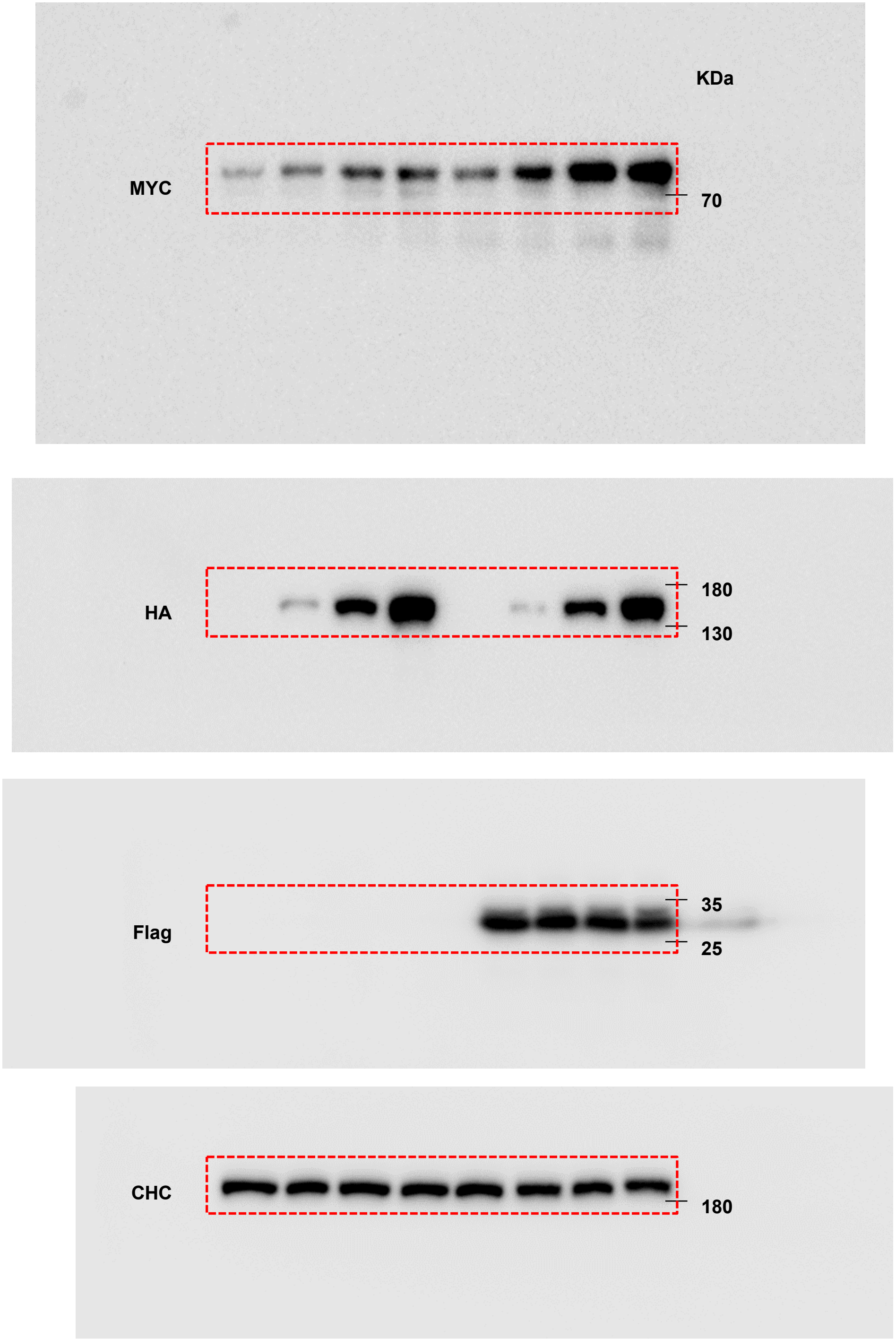

Chemiluminescence Western Blotting,
Autoradiography machine (Tanon-5200) detection

Figure S6C

Flag-Vector	+	-	-	-	-
Flag-TBC1D1^{WT}	-	+	+	-	-
Flag-TBC1D1^{S237A}	-	-	-	+	+
shRNA-NC	+	+	-	+	-
shRNA-Rab2A	-	-	+	-	+

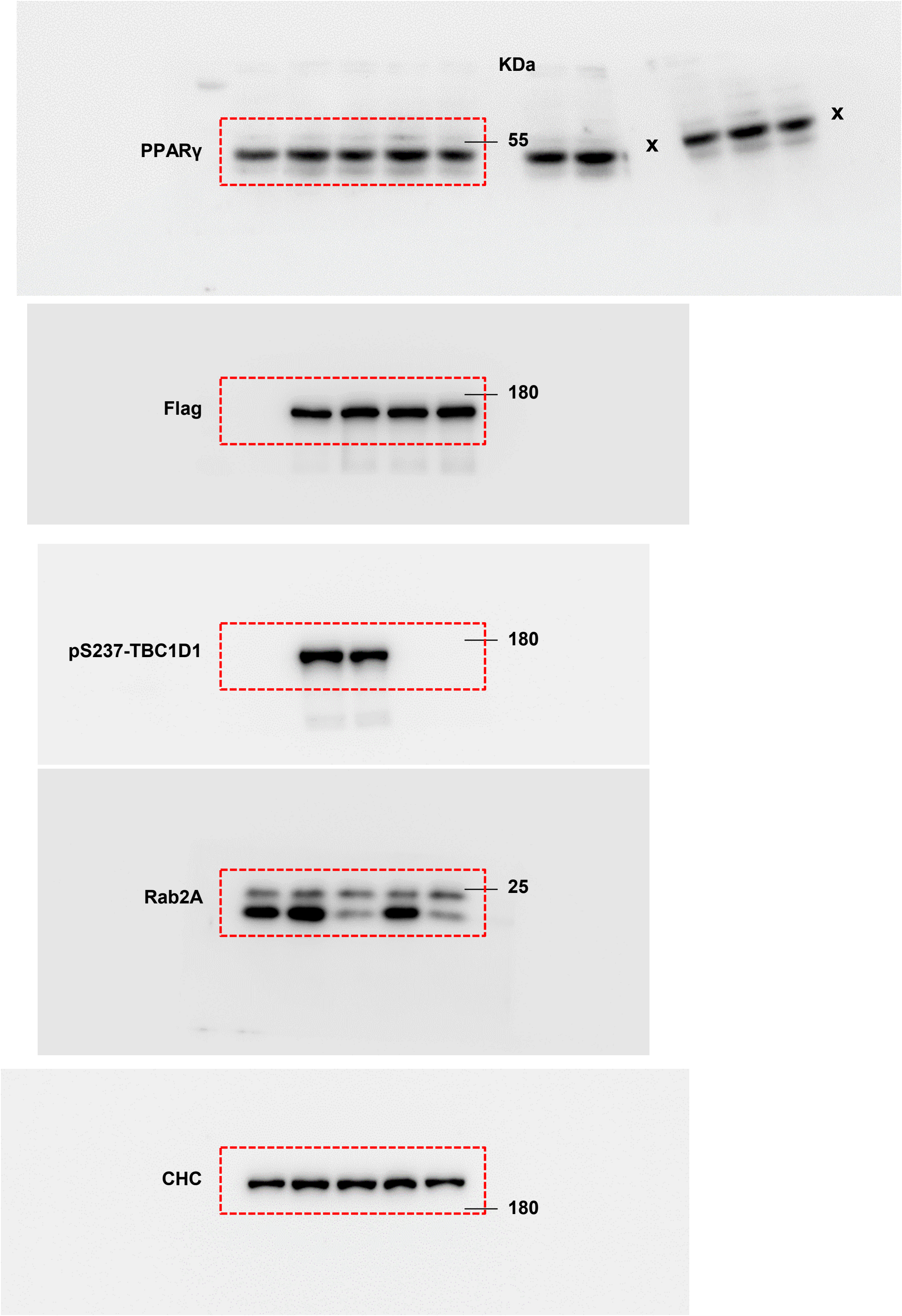

Chemiluminescence Western Blotting,
Autoradiography machine (Tanon-5200) detection

Figure S6D

SiRNA-NC	+	+	+	+	-	-	-	-
SiRNA-Rab2A	-	-	-	-	+	+	+	+
HA-TBC1D1^{WT} (μg)	-	0.1	0.3	1.0	-	0.1	0.3	1.0
PPARγ2-MYC	+	+	+	+	+	+	+	+

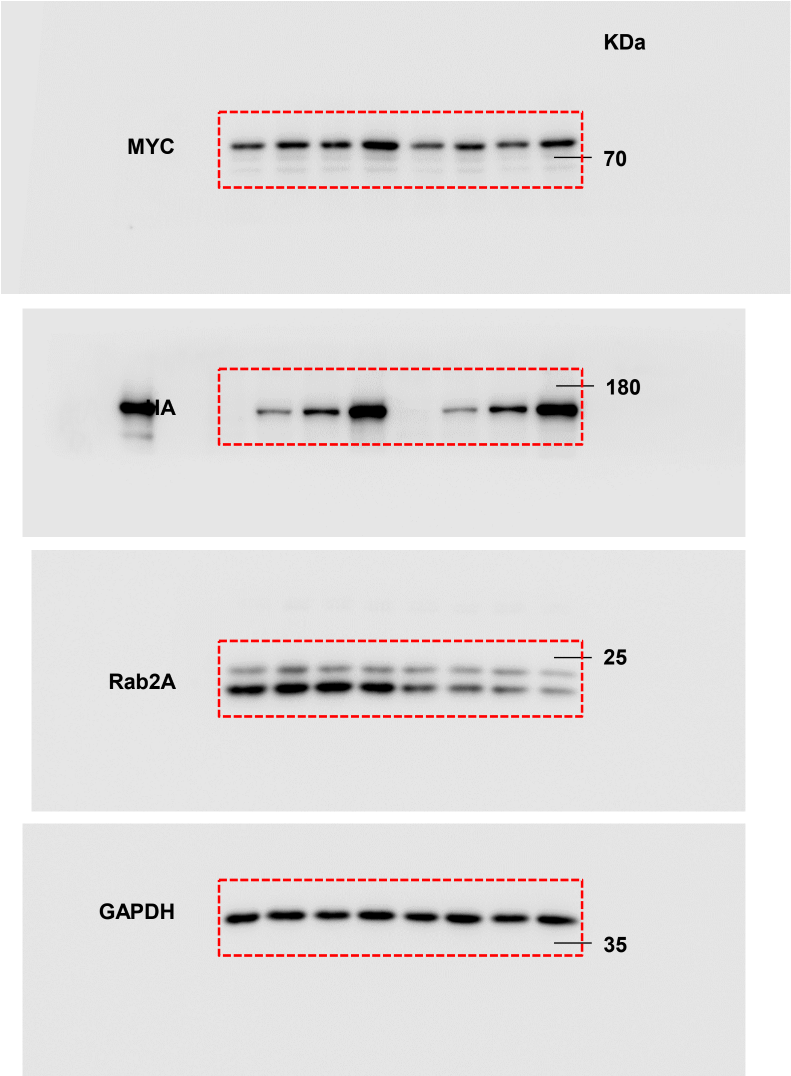

Chemiluminescence Western Blotting,
Autoradiography machine (Tanon-5200) detection

Figure S6E

SiRNA-NC	+	+	+	+	-	-	-	-
SiRNA-Rab2A	-	-	-	-	+	+	+	+
HA-TBC1D1^{S237A} (μg)	-	0.1	0.3	1.0	-	0.1	0.3	1.0
PPARγ2-MYC	+	+	+	+	+	+	+	+

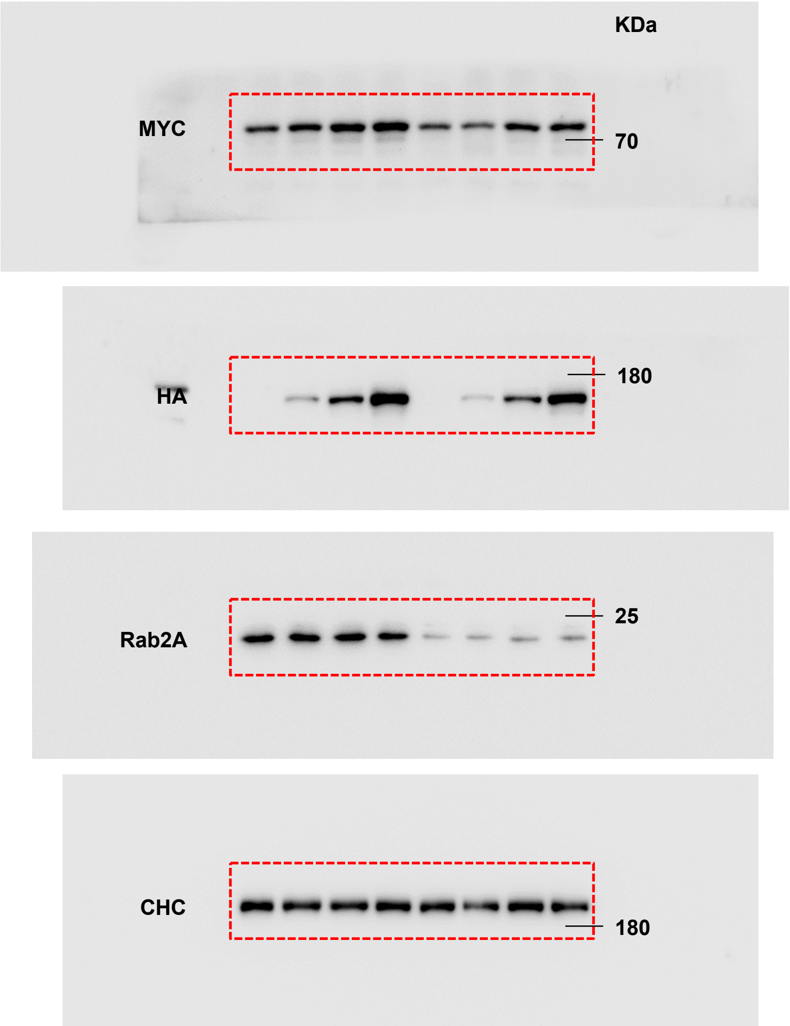

Chemiluminescence Western Blotting,
Autoradiography machine (Tanon-5200) detection

Figure S7A

Flag-Vector	+	-	-	-
Flag-Rab2A^{WT}	-	+	-	-
Flag-Rab2A^{Q65L}	-	-	+	-
Flag-Rab2A^{S20N}	-	-	-	+
PPARγ2-MYC	+	+	+	+

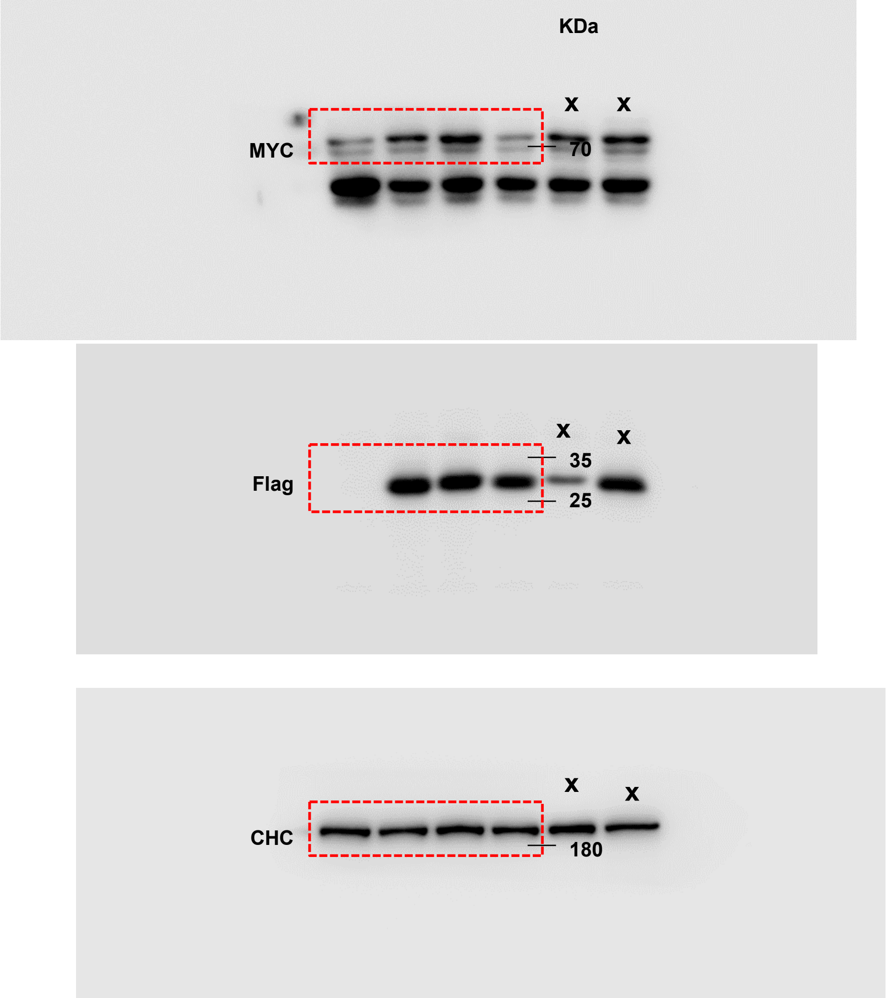

Chemiluminescence Western Blotting,
Autoradiography machine (Tanon-5200) detection

Figure S7C

Flag-Vector	+	-	+	-	+	-	+	-	+	-
Flag-Rab2A^{Q65L}	-	+	-	+	-	+	-	+	-	+
PPARγ2-MYC:FL	+	+	-	-	-	-	-	-	-	-
PPARγ2-MYC:T01	-	-	+	+	-	-	-	-	-	-
PPARγ2-MYC:T02	-	-	-	-	+	+	-	-	-	-
PPARγ2-MYC:T03	-	-	-	-	-	-	+	+	-	-
PPARγ2-MYC:T04	-	-	-	-	-	-	-	-	+	+

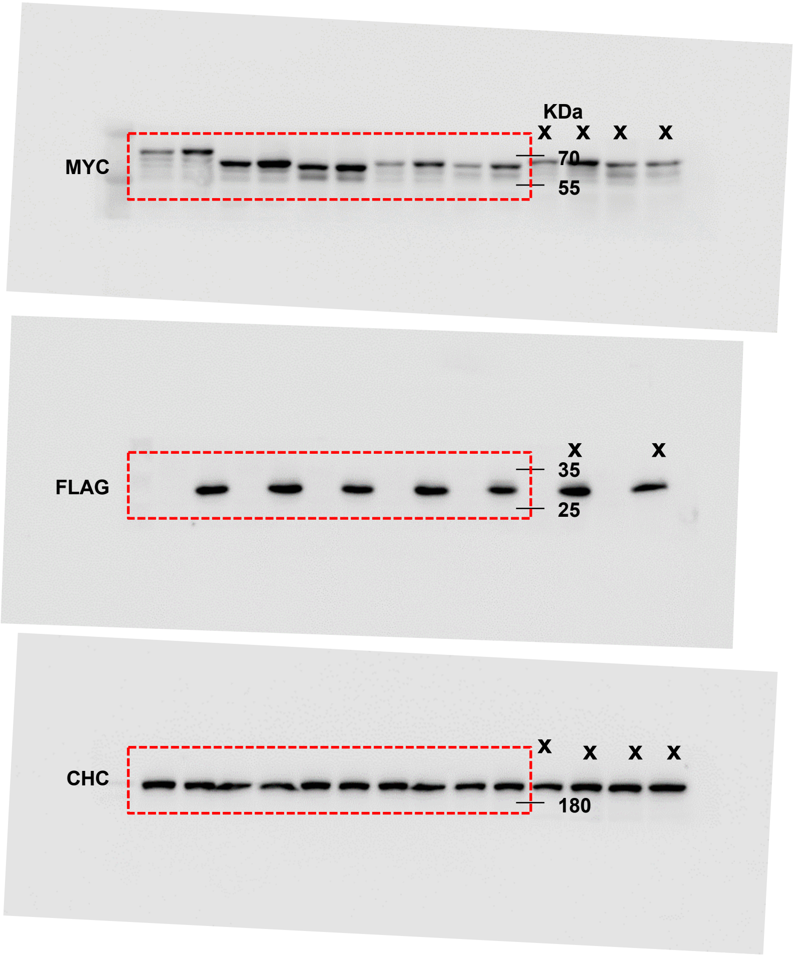

Chemiluminescence Western Blotting,
Autoradiography machine (Tanon-5200) detection

Figure S7D

Flag-Vector	+						-					
Flag-Rab2A	-						+					
PPARγ2-MYC	+											
CHX (Hrs)	-	1	2	4	6	8	-	1	2	4	6	8

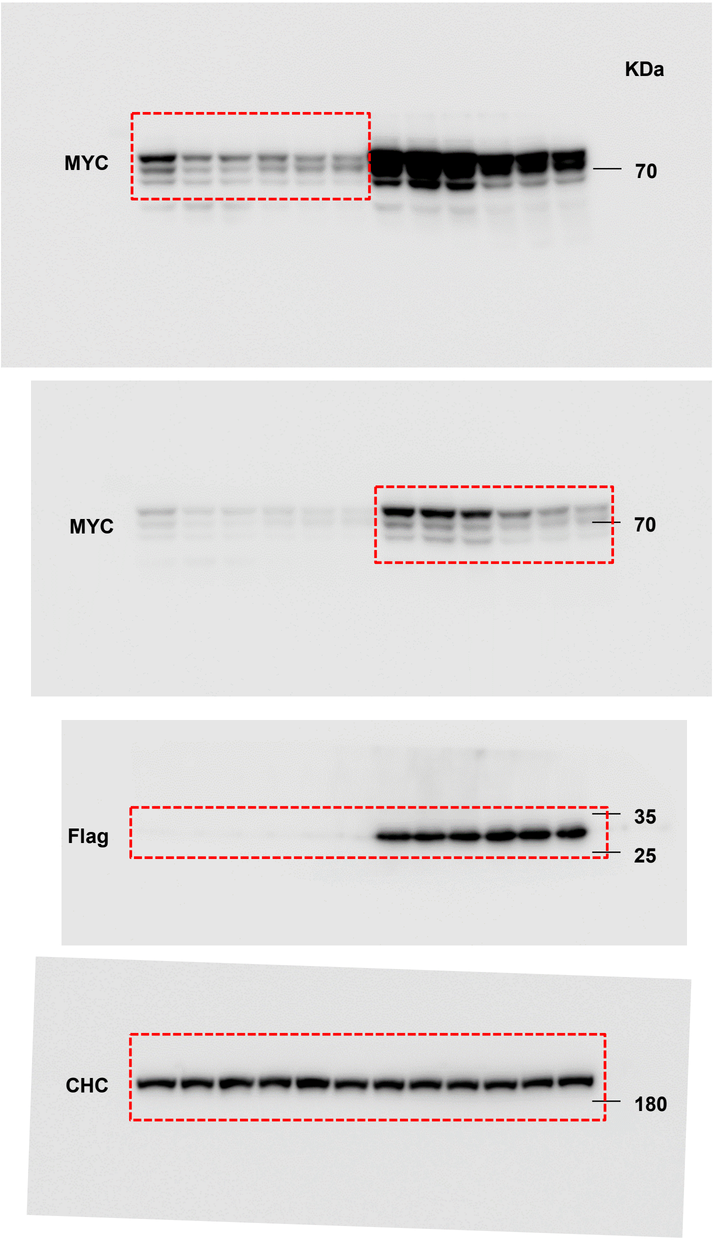

Chemiluminescence Western Blotting,
Autoradiography machine (Tanon-5200) detection

Figure S7F

Flag-Vector	+				-			
Flag-Rab2A	-				+			
PPARγ2-MYC	+							
NH_4Cl (mM)	-	10	30	50	-	10	30	50

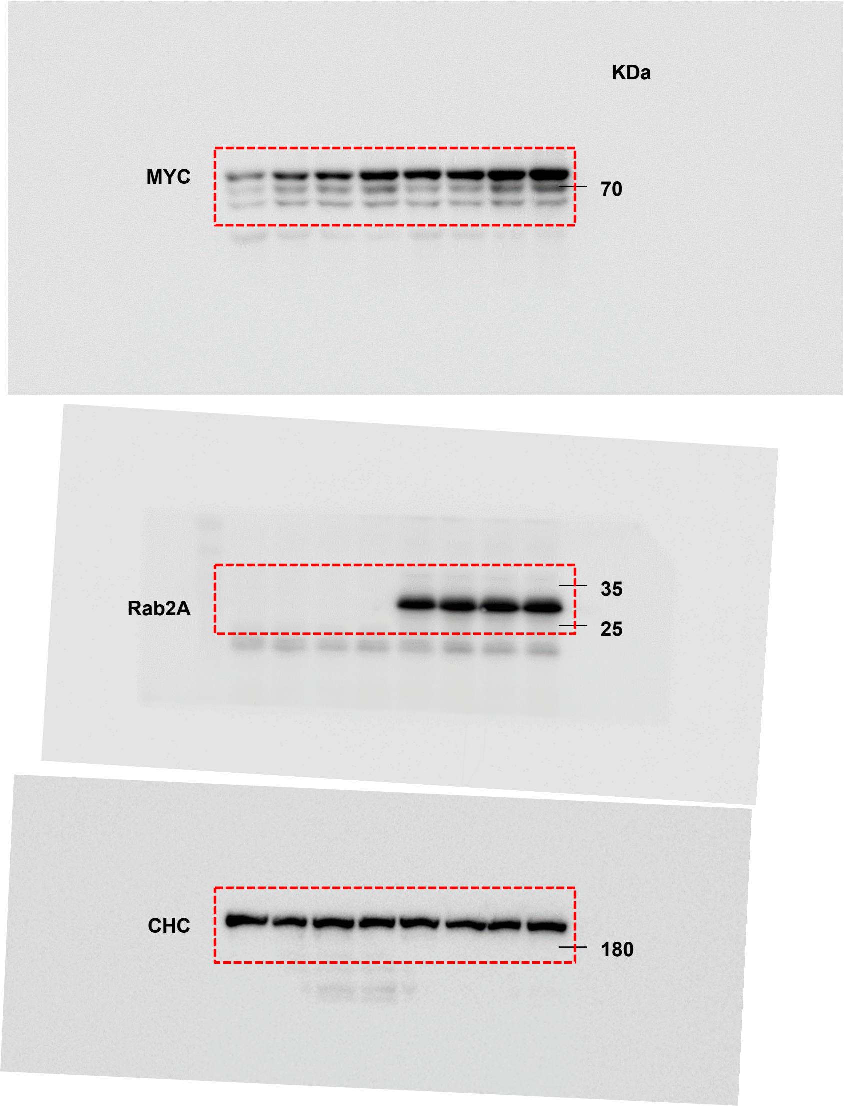

Chemiluminescence Western Blotting,
Autoradiography machine (Tanon-5200) detection

Figure S7H

Flag-Vector	+				-			
Flag-Rab2A	-				+			
PPARγ2-MYC	+							
NH_4Cl (50 mM)	0	2	4	6	0	2	4	6

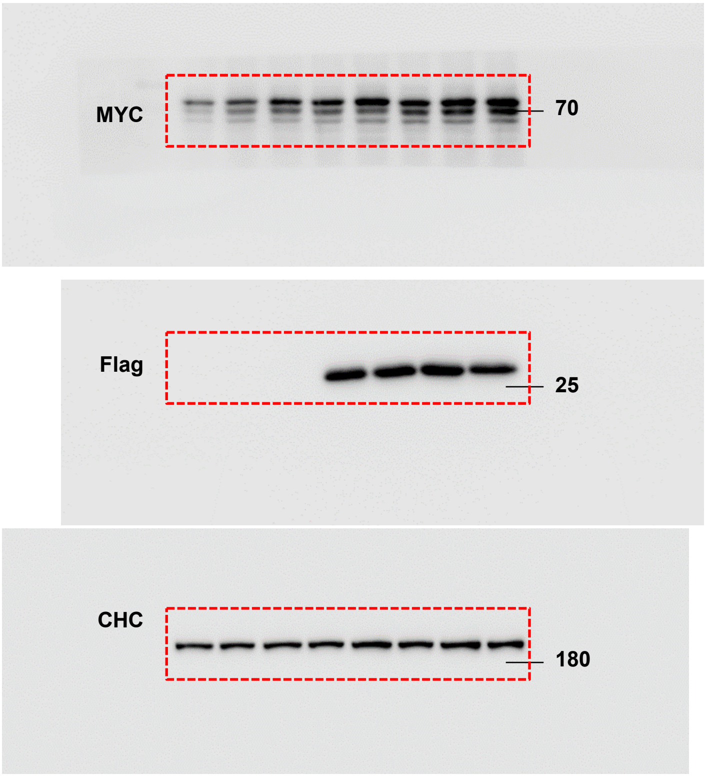

Chemiluminescence Western Blotting,
Autoradiography machine (Tanon-5200) detection

Figure S7J

Flag-Vector	+					-				
Flag-Rab2A	-					+				
PPARγ2-MYC	+									
Bafilomycin A1 (nM)	-	50	100	200	400	-	50	100	200	400

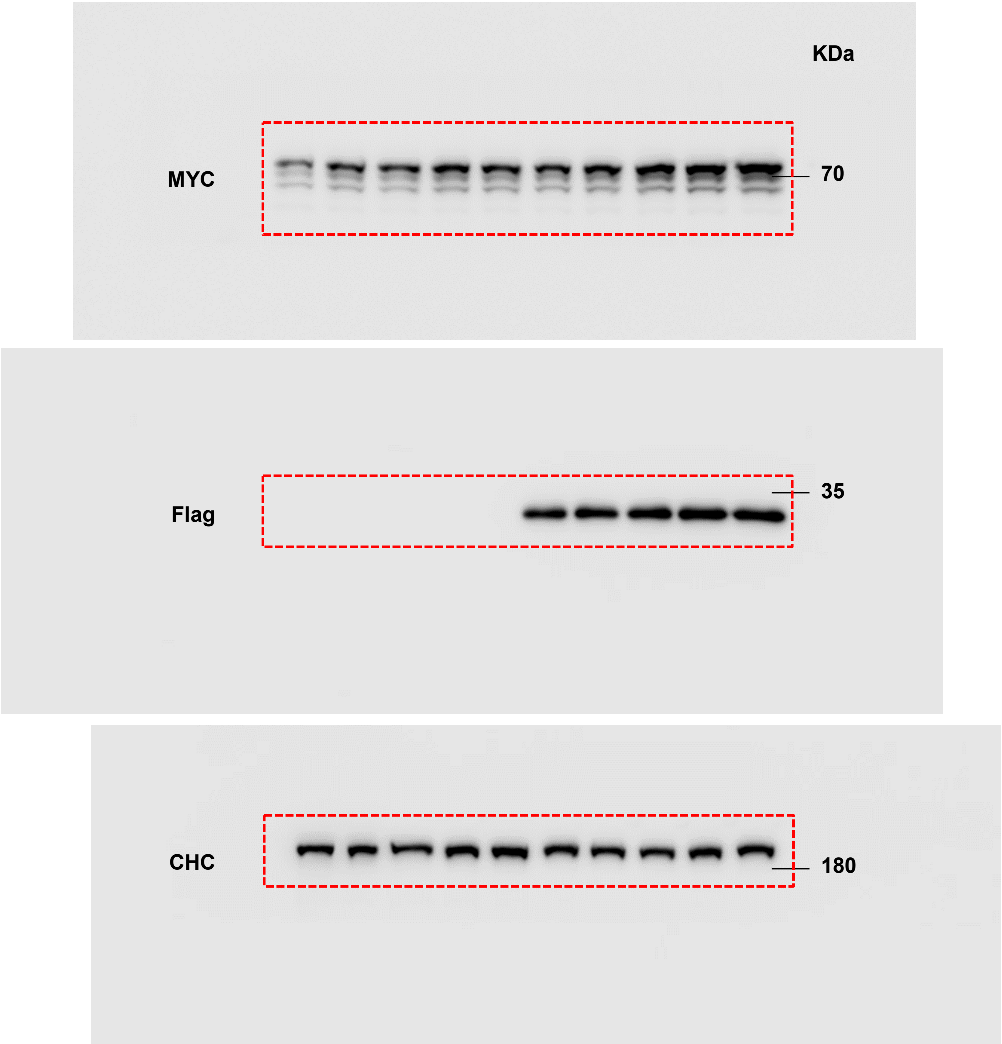

Chemiluminescence Western Blotting,
Autoradiography machine (Tanon-5200) detection

Figure S7L

Flag-Vector	+				-			
Flag-Rab2A	-				+			
PPARγ2-MYC	+							
Bafilomycin A1 (200nM)	-	2	4	6	-	2	4	6

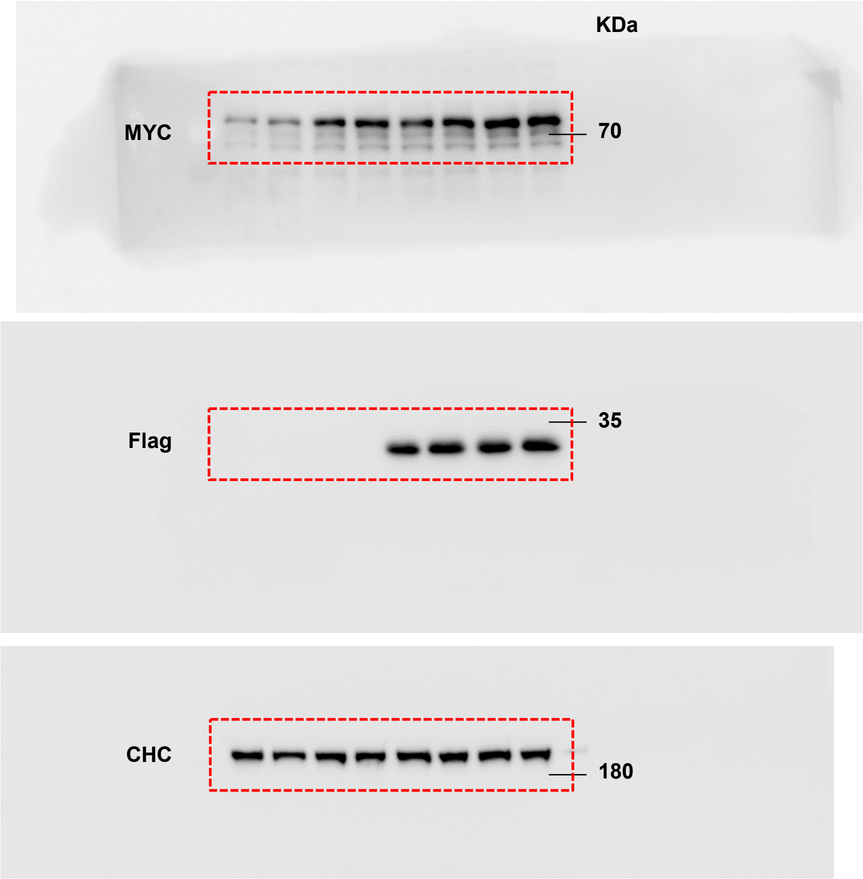

Chemiluminescence Western Blotting,
Autoradiography machine (Tanon-5200) detection
